# Supplementary figures and images for: Areas of endemism of land planarians (Platyhelminthes: Tricladida) in the Southern Atlantic Forest
Source: PLoS One. 2020 Jul 20;15(7):e0235949. doi: 10.1371/journal.pone.0235949 (PMC7371199; doi:10.1371/journal.pone.0235949)

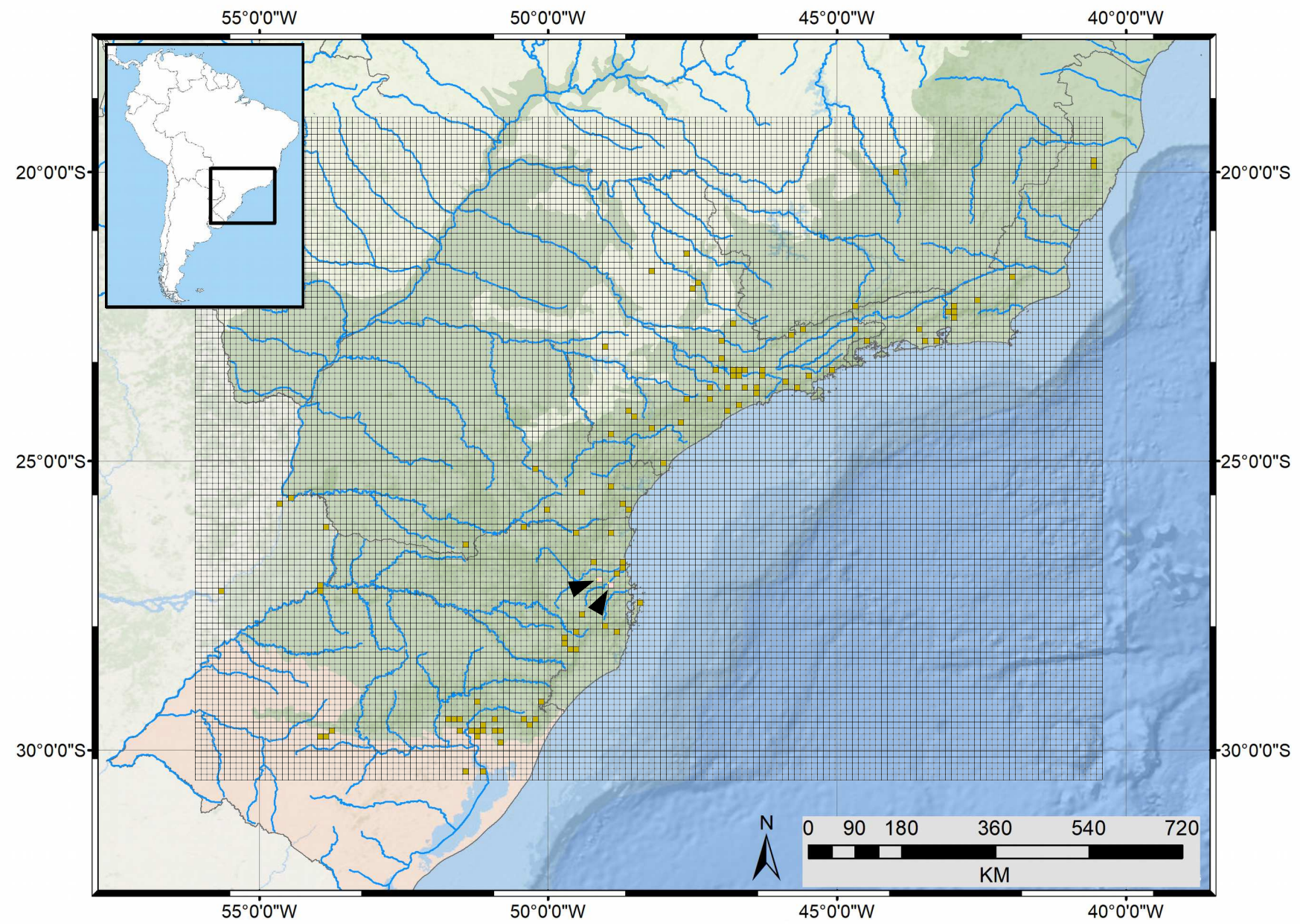

Supplement: S1 Fig — Brown cells represent areas with an Endemicity Score of 0–1.9999 (one or no endemic species found in the cell), pink cells (pointed with arrowheads) represent areas with an Endemicity Score of 2 or higher (two or more endemic species found in the cell), empty cells represent areas where no sampling was carried out. Species giving score in pink cells: Pasipha velina (Froehlich, 1959) (Endemicity Index of 1.000), Choeradoplana langi (Graff, 1894) (Endemicity Index of 1.000). (PDF) [file pone.0235949.s001.pdf]

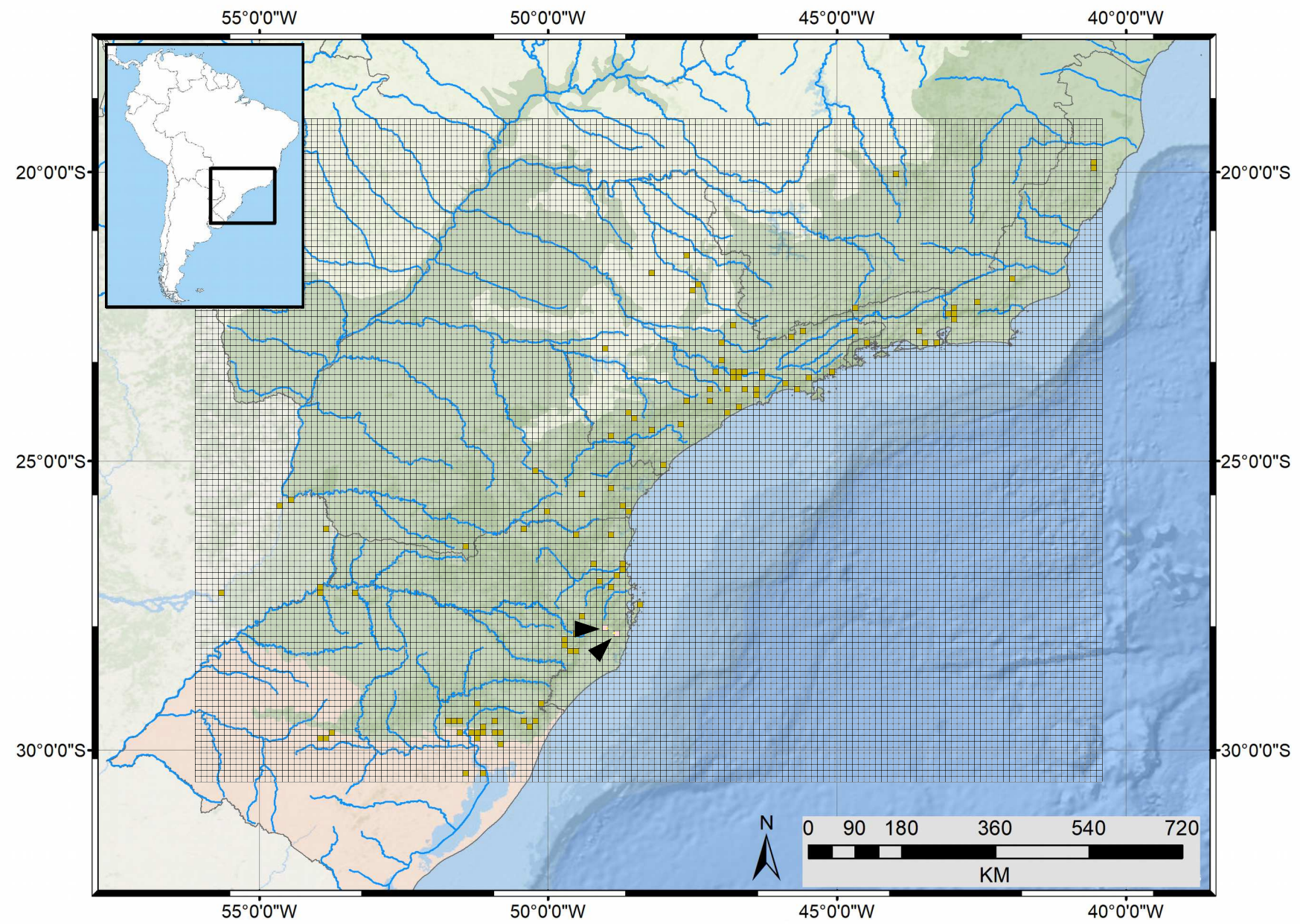

Supplement: S2 Fig — Brown cells represent areas with an Endemicity Score of 0–1.9999 (one or no endemic species found in the cell), pink cells (pointed with arrowheads) represent areas with an Endemicity Score of 2 or higher (two or more endemic species found in the cell), empty cells represent areas where no sampling was carried out. Species giving score in pink cells: Paraba tingauna (Kishimoto & Carbayo, 2012) (Endemicity Index of 1.000), Choeradoplana abaiba Carbayo et al., 2017 (Endemicity Index of 1.000). (PDF) [file pone.0235949.s002.pdf]

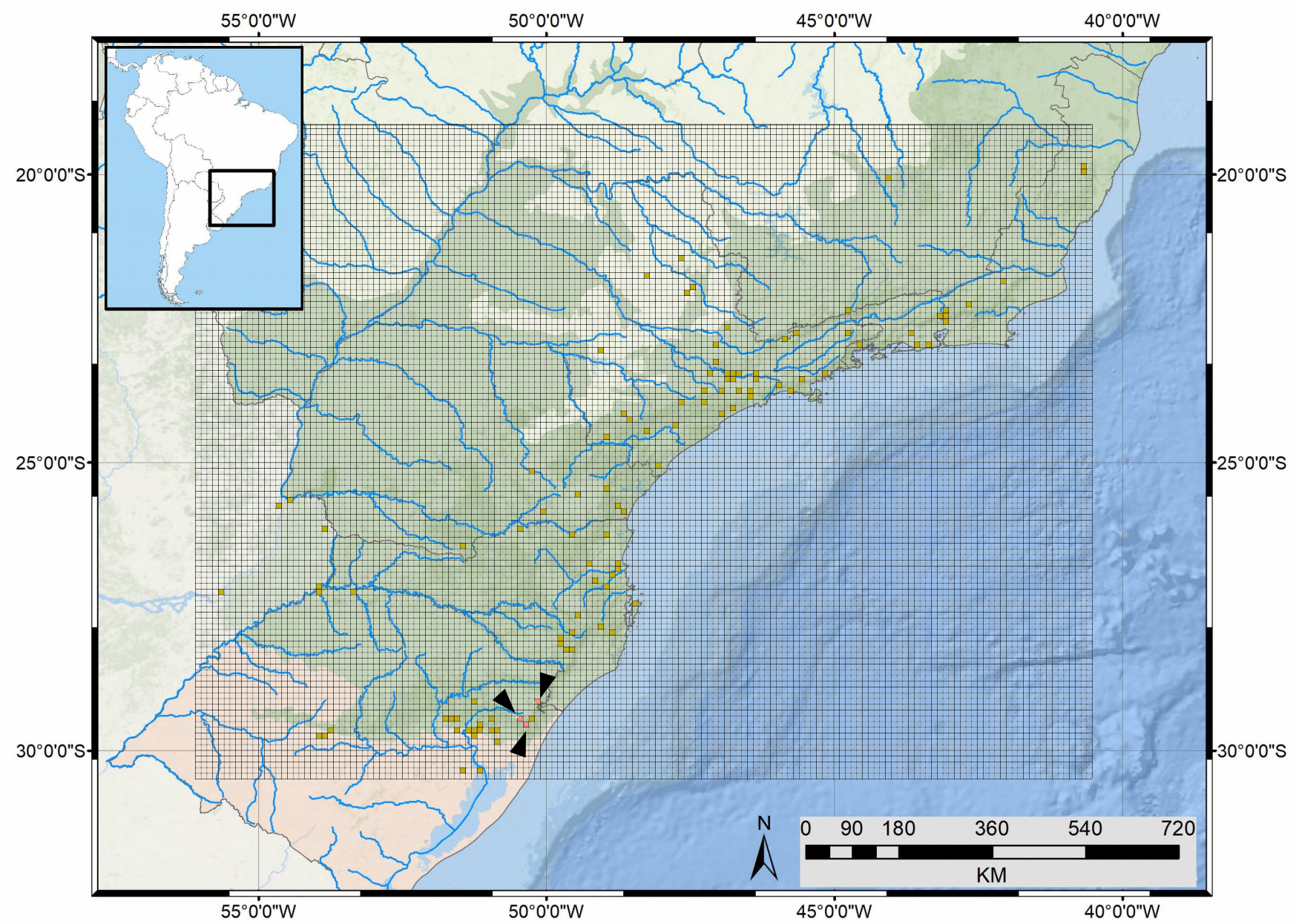

Supplement: S3 Fig — Brown cells represent areas with an Endemicity Score of 0–1.9999 (one or no endemic species found in the cell), pink cells (pointed with arrowheads) represent areas with an Endemicity Score of 2 or higher (two or more endemic species found in the cell), empty cells represent areas where no sampling was carried out. Species giving score in pink cells: Luteostriata ceciliae (Froehlich & Leal-Zanchet, 2003) (Endemicity Index of 0.833–1.000), Obama josefi (Carbayo & Leal-Zanchet, 2001) (Endemicity Index of 0.000–0.500), Paraba franciscana (Leal-Zanchet & Carbayo, 2001) (Endemicity Index of 0.000–0.500), Luteostriata arturi (Lemos & Leal-Zanchet, 2008) (Endemicity Index of 0.833–1.000), Obama maculipunctata Rossi et al., 2016 (Endemicity Index of 0.833–1.000). (PDF) [file pone.0235949.s003.pdf]

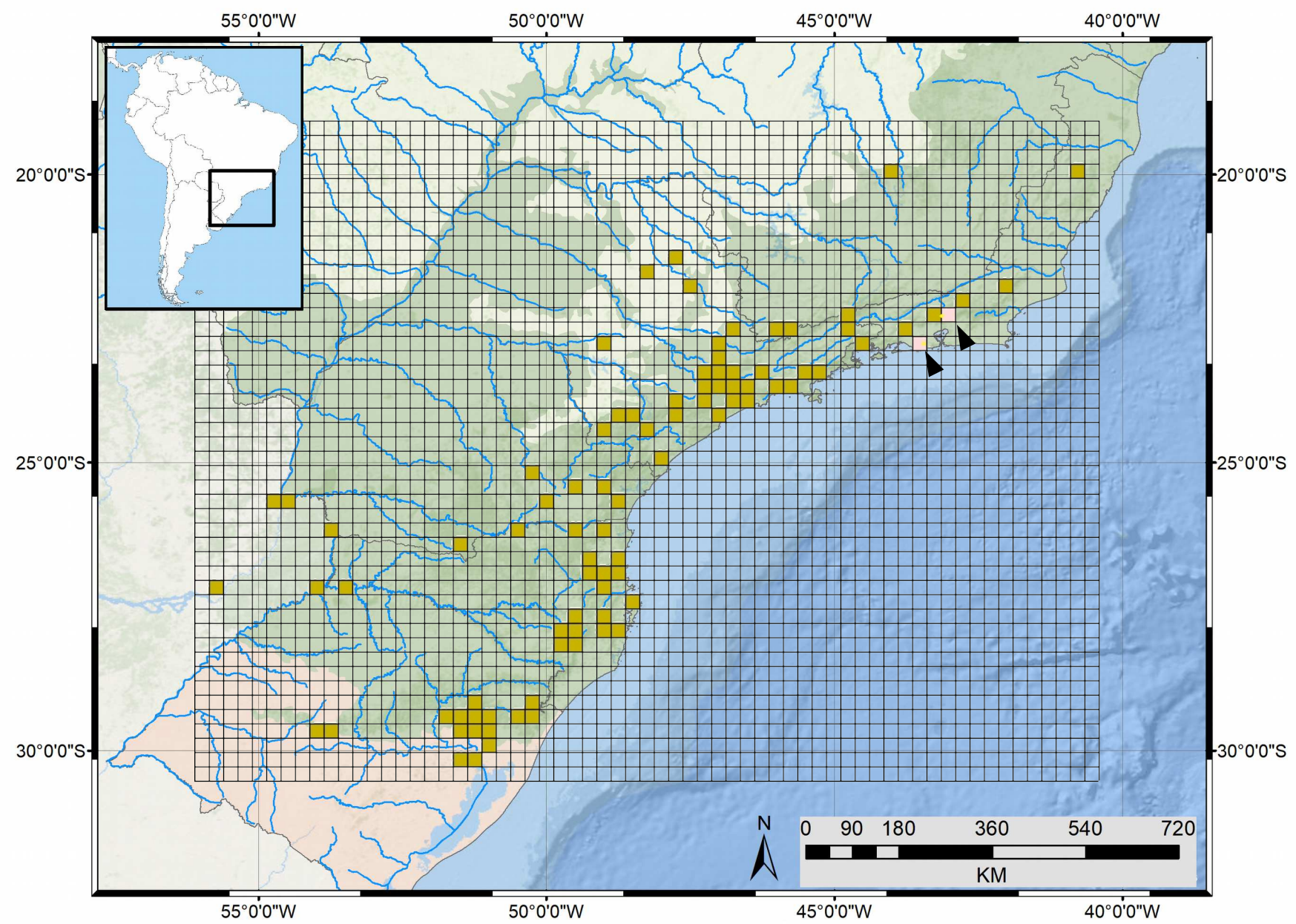

Supplement: S4 Fig — Brown cells represent areas with an Endemicity Score of 0–1.9999 (one or no endemic species found in the cell), pink cells (pointed with arrowheads) represent areas with an Endemicity Score of 2 or higher (two or more endemic species found in the cell), empty cells represent areas where no sampling was carried out. Species giving score in pink cells: Obama fryi (Graff, 1899) (Endemicity Index of 1.000), Geoplana vaginuloides (Darwin, 1844) (Endemicity Index of 1.000). (PDF) [file pone.0235949.s004.pdf]

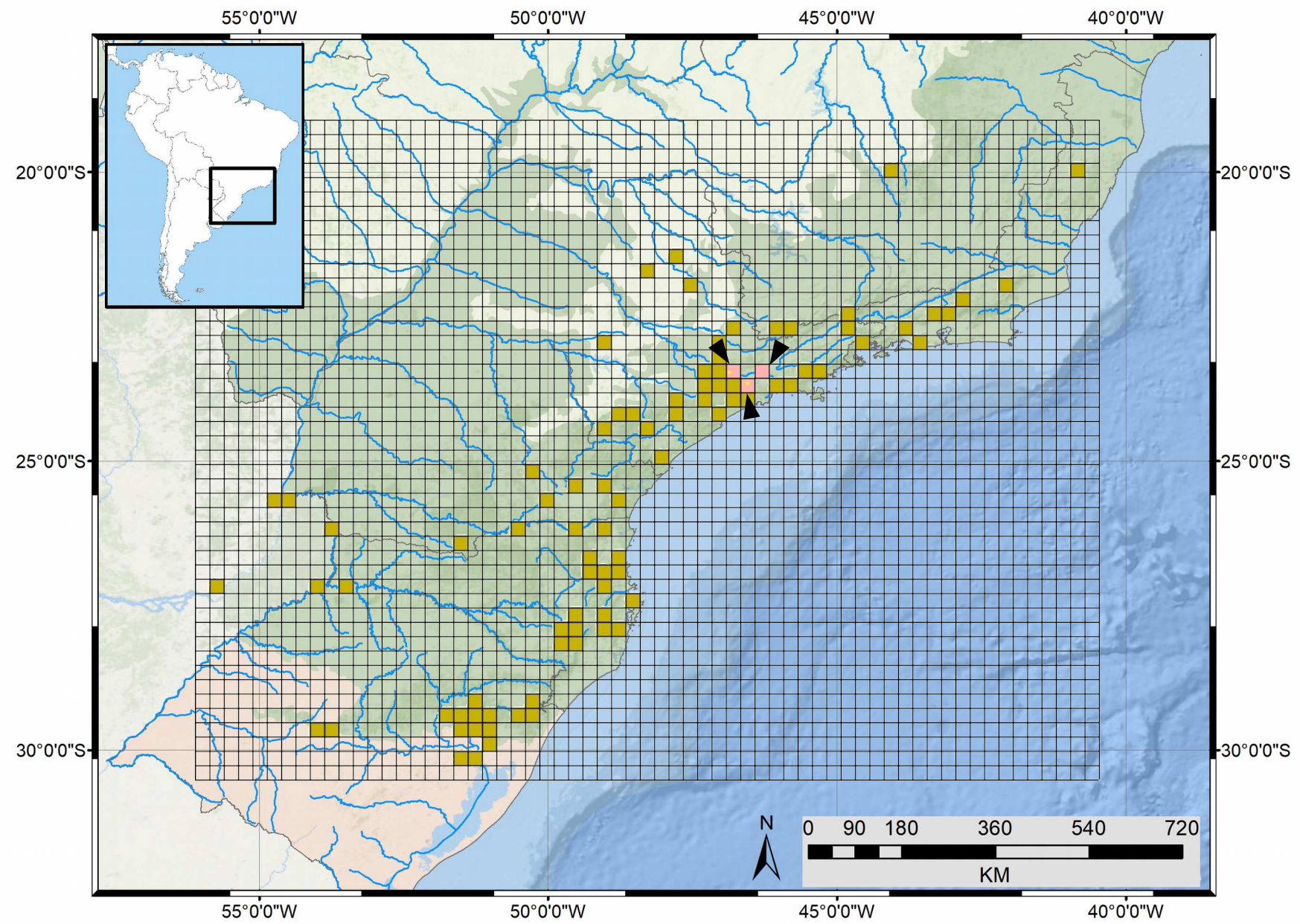

Supplement: S5 Fig — Brown cells represent areas with an Endemicity Score of 0–1.9999 (one or no endemic species found in the cell), pink cells (pointed with arrowheads) represent areas with an Endemicity Score of 2 or higher (two or more endemic species found in the cell), empty cells represent areas where no sampling was carried out. Species giving score in pink cells: Obama metzi (Graff, 1899) (Endemicity Index of 0.000–0.833), Geoplana duca Marcus, 1951 (Endemicity Index of 0.833–1.000), Geoplana mogi Almeida & Carbayo, 2019 (Endemicity Index of 0.833–1.000). (PDF) [file pone.0235949.s005.pdf]

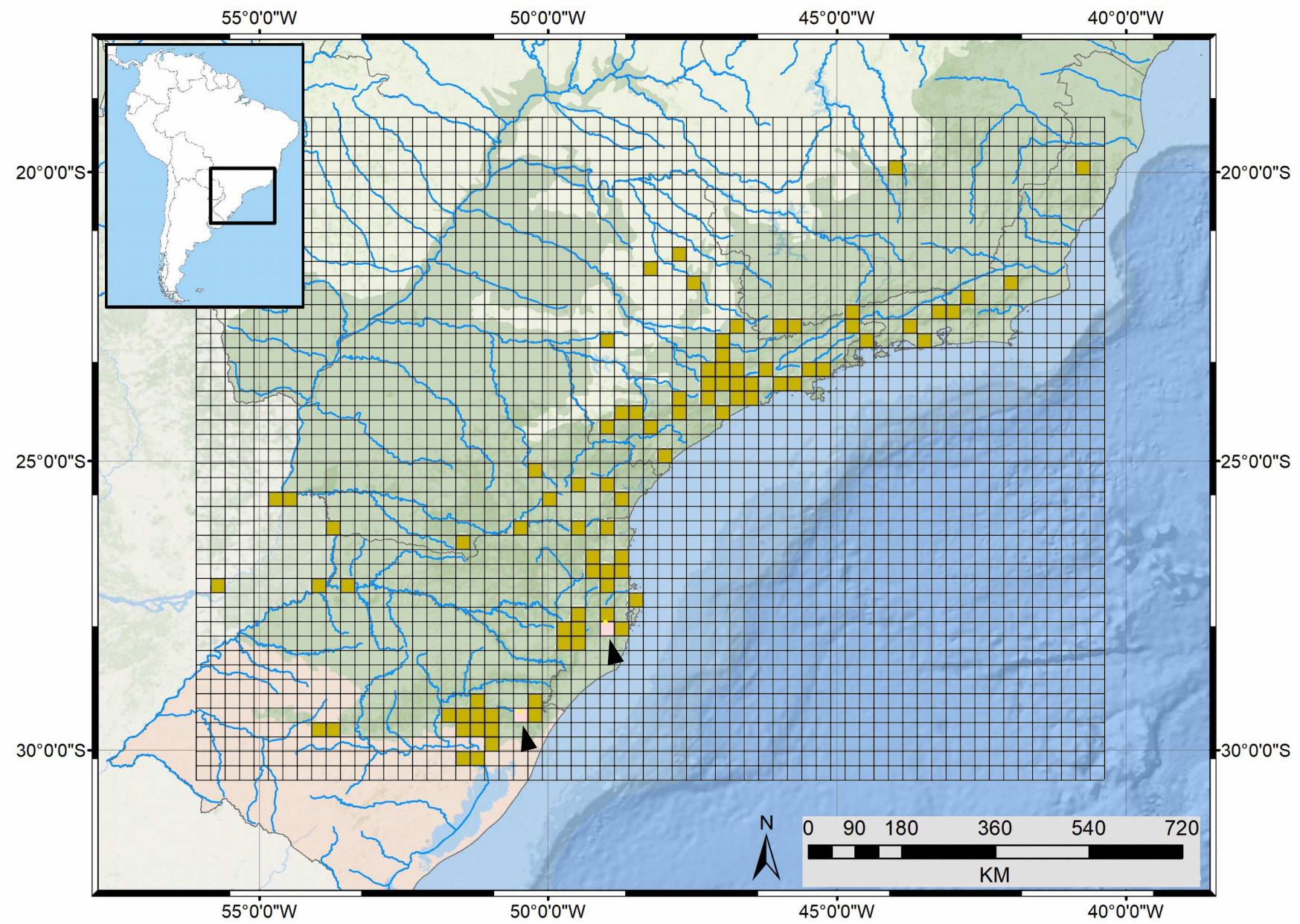

Supplement: S6 Fig — Brown cells represent areas with an Endemicity Score of 0–1.9999 (one or no endemic species found in the cell), pink cells (pointed with arrowheads) represent areas with an Endemicity Score of 2 or higher (two or more endemic species found in the cell), empty cells represent areas where no sampling was carried out. Species giving score in pink cells: Choeradoplana benyai Lemos & Leal-Zanchet, 2014 (Endemicity Index of 1.000), Imbira sp. 1 (Endemicity Index of 1.000). (PDF) [file pone.0235949.s006.pdf]

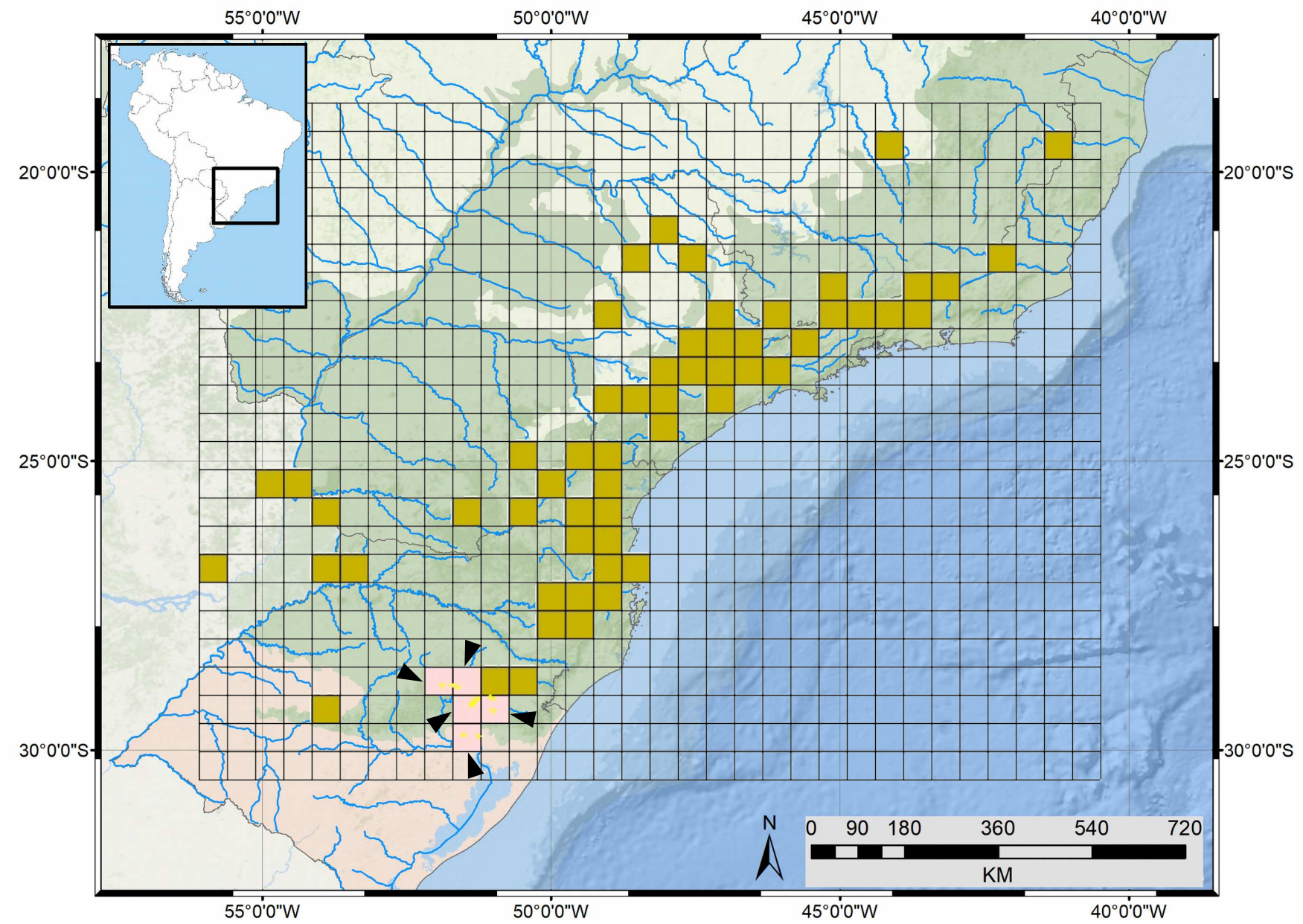

Supplement: S7 Fig — Brown cells represent areas with an Endemicity Score of 0–1.9999 (one or no endemic species found in the cell), pink cells (pointed with arrowheads) represent areas with an Endemicity Score of 2 or higher (two or more endemic species found in the cell), empty cells represent areas where no sampling was carried out. Species giving score in pink cells: Luteostriata abundans (Graff, 1899) (Endemicity Index of 0.429–1.000), Paraba gaucha (Froehlich, 1959) (Endemicity Index of 0.800–1.000), Pasipha hauseri (Froehlich, 1959) (Endemicity Index of 0.700–0.833). (PDF) [file pone.0235949.s007.pdf]

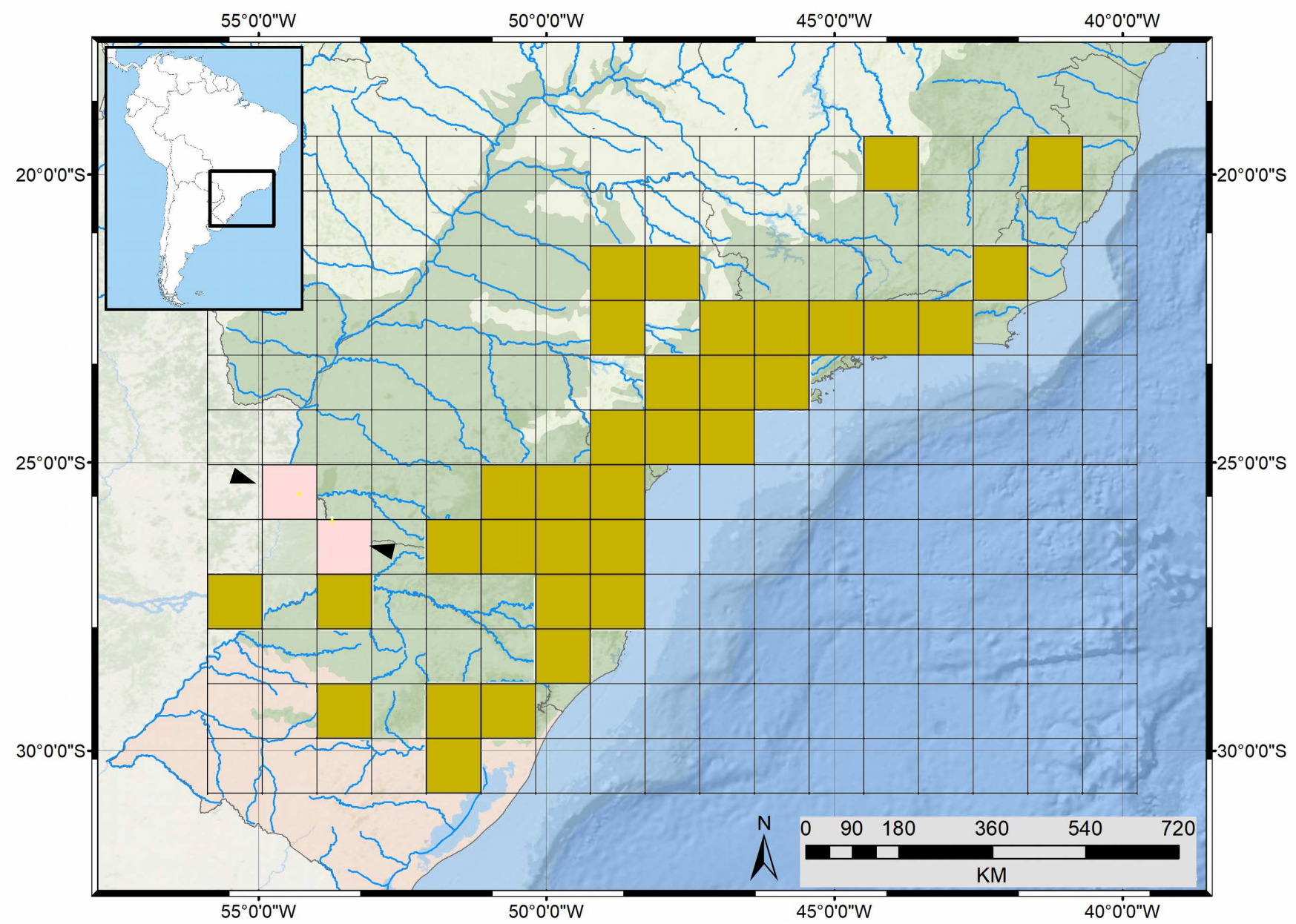

Supplement: S8 Fig — Brown cells represent areas with an Endemicity Score of 0–1.9999 (one or no endemic species found in the cell), pink cells (pointed with arrowheads) represent areas with an Endemicity Score of 2 or higher (two or more endemic species found in the cell), empty cells represent areas where no sampling was carried out. Species giving score in pink cells: Supramontana argentina Negrete et al., 2012 (Endemicity Index of 1.000), Pasipha mbya Negrete & Brusa, 2016 (Endemicity Index of 1.000). (PDF) [file pone.0235949.s008.pdf]
