# Supplementary material for: Areas of endemism of land planarians (Platyhelminthes: Tricladida) in the Southern Atlantic Forest
Source: PLoS One. 2020 Jul 20;15(7):e0235949. doi: 10.1371/journal.pone.0235949 (PMC7371199; doi:10.1371/journal.pone.0235949)
Supplement: S1 Table — Data comes from literature (reference given) and our own unpublished records (a voucher specimen is mentioned as well as the way the specimen was identified). ‘Name of sp. in EA’ reffers to the name of the species in the file S2 Material. Abbreviations: A1, anterior-most extremity of body; AC, copulatory apparatus; F, pharynx; Morph., morphology. (DOCX) [file pone.0235949.s009.docx]

**Suppl. Tab. 1**. Geographic coordinates of the species used in the study. Data come from literature (reference given) and our own unpublished records (a voucher specimen is mentioned as well as the way the specimen was identified). ‘Name of sp. in EA’ refers to the name of the species in the file Supp. Mat. 1. Abbreviations: A1, anterior-most extremity of body; AC, copulatory apparatus; F, pharynx; Morph., morphology.

| **Reference or voucher specimen of the record** | **Morph-ological identifi-cation** | **Locality** | **Name of sp. in EA** | ***Scientific name*** | **Latitude** | **Longitude** | **Country** |
| --- | --- | --- | --- | --- | --- | --- | --- |
| Lago-Barcia & Carbayo (2018) | - | Ribeirão Grande/SP | sp 0 | *Cratera arucuia* | -24.2693 | -48.4054 | Brazil |
| Amaral et al 2018 | - | Maquiné/RS | sp 1 | *Imbira flavonigra* | -29.501444 | -50.221056 | Brazil |
| Negrete & Brusa (2017) | - | San Ignacio | sp 2 | *Pasipha quirogai* | -27.27944 | -55.57861 | Argentina |
| Negrete & Brusa (2017) | - | Moconá Provincial Park, Misiones province, Argentina | sp 3 | *Imbira negrita* | -27.14889 | -53.898333 | Argentina |
| Froehlich & Leal-Zanchet, 2003 | - | São Francisco de Paula/RS | sp 4 | *Luteostriata ceciliae* | -29.428944 | -50.392000 | Brazil |
| Leal-Zanchet et al (2011) | - | Cambará do Sul/RS | sp 4 | *Luteostriata ceciliae* | -29.179104 | -50.080631 | Brazil |
| Carbayo et al (2016) | - | Paulo Lopes/SC | sp 5 | *Obama marmorata* | -27.84278 | -48.92583 | Brazil |
| Carbayo et al (2016) | - | São Bonifácio/SC | sp 5 | *Obama marmorata* | -27.84278 | -48.92583 | Brazil |
| Carbayo et al (2016) | - | Paulo Lopes/SC | sp 5 | *Obama marmorata* | -27.95869 | -48.76164 | Brazil |
| **F4620** | External | PN Itajaí | sp 5 | *Obama marmorata* | -27.05386 | -49.08606 | Brazil |
| **F7401** | External | PN São Joaquim | sp 5 | *Obama marmorata* | -28.235882 | -49.498872 | Brazil |
| Froehlich (1959) | - | Rio do Testo/SC | sp 5 | *Obama marmorata* | -26.743720 | -49.175644 | Brazil |
| Schultze & Müller (1857) | - | Blumenau/SC | sp 5 | *Obama marmorata* | -27.05386 | -49.08606 | Brazil |
| Carbayo (2010) | - | Penha/SC | sp 6 | *Luteostriata muelleri* | -26.80 | -48.65 | Brazil |
| Carbayo (2010) | - | Navegantes/SC | sp 6 | *Luteostriata muelleri* | -26.85 | -48.69 | Brazil |
| Carbayo (2010) | - | Itajaí/SC | sp 6 | *Luteostriata muelleri* | -26.88 | -48.66 | Brazil |
| Carbayo et al (2013) | - | São Bonifácio/SC | sp 6 | *Luteostriata muelleri* | -27.84278 | -48.92583 | Brazil |
| **F1588** | External and AC | PN Itajaí | sp 6 | *Luteostriata muelleri* | -27.05306 | -49.08528 | Brazil |
| **F3284** | External | PE Tabuleiro | sp 6 | *Luteostriata muelleri* | -27.840735 | -48.924866 | Brazil |
| Froehlich (1959) | - | Rio do Testo/SC | sp 6 | *Luteostriata muelleri* | -26.743720 | -49.175644 | Brazil |
| Froehlich (1959) | - | Brusque/SC | sp 6 | *Luteostriata muelleri* | -27.111325 | -48.895427 | Brazil |
| Schultze & Müller (1857) | - | Blumenau/SC | sp 6 | *Luteostriata muelleri* | -27.05386 | -49.08606 | Brazil |
| Amaral et al (2014) | - | Poço das Antas/RS | sp 7 | *Luteostriata abundans* | -29.44 | -51.68 | Brazil |
| Amaral et al (2014) | - | Salvador do Sul/RS | sp 7 | *Luteostriata abundans* | -29.442428 | -51.502151 | Brazil |
| Amaral et al (2014) | - | Tupandi/RS | sp 7 | *Luteostriata abundans* | -29.47 | -51.42 | Brazil |
| Amaral et al (2014) | - | Taquara/RS | sp 7 | *Luteostriata abundans* | -29.659801 | -50.779542 | Brazil |
| Amaral et al (2014) | - | Campo Bom/RS | sp 7 | *Luteostriata abundans* | -29.67 | -51.06 | Brazil |
| Amaral et al (2014) | - | Novo Hamburgo/RS | sp 7 | *Luteostriata abundans* | -29.72 | -51.12 | Brazil |
| Amaral et al (2014) | - | Glorinha/RS | sp 7 | *Luteostriata abundans* | -29.88 | -50.79 | Brazil |
| Antunes et al (2008) | - | Viamão/RS | sp 7 | *Luteostriata abundans* | -30.345738 | -51.027817 | Brazil |
| Carbayo (2010) | - | Barra do Ribeiro/RS | sp 7 | *Luteostriata abundans* | -30.31 | -51.32 | Brazil |
| Carbayo et al (2013) | - | Parobé/RS | sp 7 | *Luteostriata abundans* | -29.62940 | -50.83110 | Brazil |
| Carbayo et al (2013) | - | São Leopoldo/RS | sp 7 | *Luteostriata abundans* | -29.78828 | -51.15084 | Brazil |
| Froehlich (1959) | - | São Leopoldo/RS | sp 7 | *Luteostriata abundans* | -29.764171 | -51.155840 | Brazil |
| Froehlich (1959) | - | São Leopoldo/RS | sp 8 | *Geoplana carrierei sensu Marcus, 1951* | -29.764171 | -51.155840 | Brazil |
| Marcus (1951) | - | São Paulo/SP | sp 8 | *Geoplana carrierei sensu Marcus, 1951* | -23.561966 | -46.656958 | Brazil |
| Marcus (1951) | - | Santo André/SP | sp 8 | *Geoplana carrierei sensu Marcus, 1951* | -23.773397 | -46.333248 | Brazil |
| Álvarez-Presas et al (2015) | - | São Bonifácio/SC | sp 9 | *Obama ladislavii* | -27.84278 | -48.92583 | Brazil |
| Álvarez-Presas et al (2015) | - | Montenegro/RS | sp 9 | *Obama ladislavii* | -29.688889 | -51.466944 | Brazil |
| Amaral et al (2014) | - | Salvador do Sul/RS | sp 9 | *Obama ladislavii* | -29.442428 | -51.502151 | Brazil |
| Antunes et al (2008) | - | Viamão/RS | sp 9 | *Obama ladislavii* | -30.345738 | -51.027817 | Brazil |
| Antunes et al (2012) | - | São Francisco de Paula/RS | sp 9 | *Obama ladislavii* | -29.428944 | -50.392000 | Brazil |
| Baptista et al (2006) | - | Cambará do Sul/RS | sp 9 | *Obama ladislavii* | -29.179104 | -50.080631 | Brazil |
| Castro & Leal_Zanchet (2005) | - | Arroio Grande/RS | sp 9 | *Obama ladislavii* | -29.672918 | -53.668858 | Brazil |
| **F3262** | External | PE Tabuleiro | sp 9 | *Obama ladislavii* | -27.840735 | -48.924866 | Brazil |
| **F6545** | External | Alfredo Wagner | sp 9 | *Obama ladislavii* | -24.61695 | -49.35075 | Brazil |
| **F7357** | External | PN São Joaquim | sp 9 | *Obama ladislavii* | -28.038956 | -49.614243 | Brazil |
| Froehlich (1959) | - | São Leopoldo/RS | sp 9 | *Obama ladislavii* | -29.764171 | -51.155840 | Brazil |
| Graff (1899) | - | Blumenau/SC | sp 9 | *Obama ladislavii* | -27.05386 | -49.08606 | Brazil |
| Graff (1899) | - | Taquara/RS | sp 9 | *Obama ladislavii* | -29.659801 | -50.779542 | Brazil |
| **F1465** | External, F and AC | PNItajai | sp 10 | *Xerapoa pseudorhynchodemus* | -27.05306 | -49.08528 | Brazil |
| **F1708** | External | Saint-Hilaire | sp 10 | *Xerapoa pseudorhynchodemus* | -25.469604 | -48.813739 | Brazil |
| **F3350** | External and AC | PE Tabuleiro | sp 10 | *Xerapoa pseudorhynchodemus* | -27.840735 | -48.924866 | Brazil |
| Froehlich (1959) | - | Blumenau/SC | sp 10 | *Xerapoa pseudorhynchodemus* | -27.05386 | -49.08606 | Brazil |
| Marcus (1951) | - | São Paulo/SP | sp 10 | *Xerapoa pseudorhynchodemus* | -23.500212 | -46.702310 | Brazil |
| Riester (1938) | - | Teresópolis/RJ | sp 10 | *Xerapoa pseudorhynchodemus* | -22.439089 | -42.994351 | Brazil |
| CG Froehlich (1956a) | - | Teresópolis/RJ | sp 11 | *Geoplana quagga* | -22.439089 | -42.994351 | Brazil |
| Froehlich (1959) | - | Blumenau/SC | sp 11 | *Geoplana quagga* | -27.05386 | -49.08606 | Brazil |
| Marcus (1951) | - | São Paulo/SP | sp 11 | *Geoplana quagga* | -23.577633 | -46.701739 | Brazil |
| Carbayo et al (2013) | - | São Paulo/SP | sp 12 | *Pasipha tapetilla* | -23.46047 | -46.63672 | Brazil |
| CG Froehlich (1956a) | - | Teresópolis/RJ | sp 12 | *Pasipha tapetilla* | -22.500057 | -42.997365 | Brazil |
| CG Froehlich (1956a) | - | Rio de Janeiro/RJ | sp 12 | *Pasipha tapetilla* | -22.975852 | -43.292230 | Brazil |
| CG Froehlich (1956a) | - | Ubatuba/SP | sp 12 | *Pasipha tapetilla* | -23.423381 | -45.083399 | Brazil |
| **F1405** | External and AC | PNItajai | sp 12 | *Pasipha tapetilla* | -27.05306 | -49.08528 | Brazil |
| **F3201** | External | PE Tabuleiro | sp 12 | *Pasipha tapetilla* | -27.94 | -48.79 | Brazil |
| Froehlich (1959) | - | Itajaí/SC | sp 12 | *Pasipha tapetilla* | -26.957485 | -48.756133 | Brazil |
| Froehlich (1959) | - | Blumenau/SC | sp 12 | *Pasipha tapetilla* | -27.05386 | -49.08606 | Brazil |
| Marcus (1951) | - | Pirassununga/SP | sp 12 | *Pasipha tapetilla* | -22.007522 | -47.459448 | Brazil |
| Froehlich (1959) | - | Blumenau/SC | sp 13 | *Pasipha velina* | -27.05386 | -49.08606 | Brazil |
| Froehlich (1959) | - | Brusque/SC | sp 13 | *Pasipha velina* | -27.111325 | -48.895427 | Brazil |
| **F3287** | External, F and AC | PE Tabuleiro | sp 14 | *Obama apeva* | -27.840735 | -48.924866 | Brazil |
| **F6512** | External | Alfredo Wagner/SC | sp 14 | *Obama apeva* | -27.61727 | -49.35075 | Brazil |
| Froehlich (1959) | - | Blumenau/SC | sp 14 | *Obama apeva* | -27.05386 | -49.08606 | Brazil |
| Froehlich (1959) | - | Brusque/SC | sp 14 | *Obama apeva* | -27.111325 | -48.895427 | Brazil |
| Froehlich (1959) | - | Rio do Testo/SC | sp 15 | *Obama assu* | -26.743720 | -49.175644 | Brazil |
| Froehlich (1959) | - | Blumenau/SC | sp 15 | *Obama assu* | -27.05386 | -49.08606 | Brazil |
| Froehlich (1959) | - | Blumenau/SC | sp 16 | *Luteostriata fita* | -27.05386 | -49.08606 | Brazil |
| Antunes et al (2008) | - | São Leopoldo/RS | sp 17 | *Paraba gaucha* | -29.764171 | -51.155840 | Brazil |
| Antunes et al (2008) | - | Viamão/RS | sp 17 | *Paraba gaucha* | -30.345738 | -51.027817 | Brazil |
| Froehlich (1959) | - | Salvador do Sul/RS | sp 17 | *Paraba gaucha* | -29.436817 | -51.527611 | Brazil |
| froehlich (1959) | - | Iraí/RS | sp 18 | *Obama glieschi* | -27.242571 | -53.272863 | Brazil |
| Amaral et al (2014) | - | Salvador do Sul/RS | sp 19 | *Pasipha hauseri* | -29.436817 | -51.527611 | Brazil |
| Froehlich (1959) | - | São Leopoldo/RS | sp 19 | *Pasipha hauseri* | -29.764171 | -51.155840 | Brazil |
| Froehlich (1959) | - | Blumenau/SC | sp 20 | *Cephaloflexa nataliae* | -27.05386 | -49.08606 | Brazil |
| Froehlich (1959) | - | Blumenau/SC | sp 21 | *Paraba suva* | -27.05386 | -49.08606 | Brazil |
| **F1634** | External, A1, F and AC | Saint-Hilaire | sp 22 | *Luteostriata sp 4* | -25.71 | -48.62 | Brazil |
| Froehlich (1959) | - | Lapa/PR | sp 23 | *Notogynaphallia atra* | -25.834333 | -49.904062 | Brazil |
| Froehlich (1959) | - | Rio do Testo/SC | sp 23 | *Notogynaphallia atra* | -26.743720 | -49.175644 | Brazil |
| Froehlich (1959) | - | Itajaí/SC | sp 23 | *Notogynaphallia atra* | -26.957485 | -48.756133 | Brazil |
| Froehlich (1959) | - | Brusque/SC | sp 23 | *Notogynaphallia atra* | -27.111325 | -48.895427 | Brazil |
| Graff (1899) | - | Taquara/RS | sp 23 | *Notogynaphallia atra* | -29.659801 | -50.779542 | Brazil |
| Schultze & Müller (1857) | - | Blumenau/SC | sp 23 | *Notogynaphallia atra* | -27.05386 | -49.08606 | Brazil |
| Graff (1899) | - | Taquara/RS | sp 24 | *Geoplana nigra* | -29.659801 | -50.779542 | Brazil |
| Álvarez-Presas et al (2011) | - | São José do Barreiro/SP | sp 25 | *Cephaloflexa bergi* | -22.72583 | -44.62611 | Brazil |
| Álvarez-Presas et al (2014) | - | Santa Teresa/ES | sp 25 | *Cephaloflexa bergi* | -19.8781 | -40.5385 | Brazil |
| Álvarez-Presas et al (2014) | - | Santa Teresa/ES | sp 25 | *Cephaloflexa bergi* | -19.8875 | -40.5362 | Brazil |
| Álvarez-Presas et al (2014) | - | Santa Teresa/ES | sp 25 | *Cephaloflexa bergi* | -19.9123 | -40.5362 | Brazil |
| Álvarez-Presas et al (2014) | - | Santa Maria Madalena/RJ | sp 25 | *Cephaloflexa bergi* | -21.8735 | -41.9142 | Brazil |
| Álvarez-Presas et al (2014) | - | Santa Maria Madalena/RJ | sp 25 | *Cephaloflexa bergi* | -21.8768 | -41.9228 | Brazil |
| Álvarez-Presas et al (2014) | - | Teresópolis/RJ | sp 25 | *Cephaloflexa bergi* | -22.4558 | -42.9963 | Brazil |
| Álvarez-Presas et al (2014) | - | Campos do Jordão/SP | sp 25 | *Cephaloflexa bergi* | -22.8039 | -45.7768 | Brazil |
| Álvarez-Presas et al (2014) | - | São Paulo/SP | sp 25 | *Cephaloflexa bergi* | -23.4300 | -46.6333 | Brazil |
| Álvarez-Presas et al (2014) | - | Salesópolis/SP | sp 25 | *Cephaloflexa bergi* | -23.6527 | -45.8909 | Brazil |
| Álvarez-Presas et al (2014) | - | Ribeirão Grande/SP | sp 25 | *Cephaloflexa bergi* | -24.2749 | -48.4162 | Brazil |
| Álvarez-Presas et al (2014) | - | São Bonifácio/SC | sp 25 | *Cephaloflexa bergi* | -27.8417 | -48.9254 | Brazil |
| Álvarez-Presas et al (2014) | - | Paulo Lopes/SC | sp 25 | *Cephaloflexa bergi* | -27.9568 | -48.7735 | Brazil |
| Baptista et al (2006) | - | Cambará do Sul/RS | sp 25 | *Cephaloflexa bergi* | -29.179104 | -50.080631 | Brazil |
| Carbayo et al (2013) | - | São Sebastião/SP | sp 25 | *Cephaloflexa bergi* | -23.75203 | -45.631027 | Brazil |
| **F1387** | External and AC | PN Itajaí | sp 25 | *Cephaloflexa bergi* | -27.04836 | -49.09206 | Brazil |
| **F1664** | External, F and AC | PN Itajai | sp 25 | *Cephaloflexa bergi* | -25.469604 | -48.813739 | Brazil |
| **F3088** | External | PE Intervales | sp 25 | *Cephaloflexa bergi* | -24.269387 | -48.405467 | Brazil |
| **F3236** | External and AC | PE Tabuleiro | sp 25 | *Cephaloflexa bergi* | -27.84 | -48.92 | Brazil |
| **F3257** | External | PE Tabuleiro | sp 25 | *Cephaloflexa bergi* | -27.9813889 | -48.748055555 | Brazil |
| **F3611** | External, F and AC | PN Saint Hilaire | sp 25 | *Cephaloflexa bergi* | -25.469604 | -48.813739 | Brazil |
| **F7275** | External, A1 and AC | PN São Joaquim | sp 25 | *Cephaloflexa bergi* | -28.041154 | -49.615693 | Brazil |
| **F7442** | External | PN São Joaquim | sp 25 | *Cephaloflexa bergi* | -27.959870 | -49.482576 | Brazil |
| Froehlich (1956a) | - | Ubatuba/SP | sp 25 | *Cephaloflexa bergi* | -23.423381 | -45.083399 | Brazil |
| Froehlich (1956b) | - | Morretes/PR | sp 25 | *Cephaloflexa bergi* | -25.483460 | -48.832020 | Brazil |
| Froehlich (1956b) | - | Curitiba/PR | sp 25 | *Cephaloflexa bergi* | -25.548339 | -49.313845 | Brazil |
| Froehlich (1956b) | - | Blumenau/SC | sp 25 | *Cephaloflexa bergi* | -27.05386 | -49.08606 | Brazil |
| Carbayo et al (2016) | - | Teresópolis/RJ | sp 26 | *Obama burmeisteri* | -22.439089 | -42.994351 | Brazil |
| Carbayo et al (2016) | - | Jundiaí/SP | sp 26 | *Obama burmeisteri* | -23.21 | -46.95 | Brazil |
| CG Froehlich (1956a) | - | Rio de Janeiro/RJ | sp 26 | *Obama burmeisteri* | -22.975852 | -43.292230 | Brazil |
| Froehlich (1956a) | - | Ubatuba/SP | sp 26 | *Obama burmeisteri* | -23.423381 | -45.083399 | Brazil |
| Froehlich (1956b) | - | Matinhos/PR | sp 26 | *Obama burmeisteri* | -25.817820 | -48.549932 | Brazil |
| Froehlich (1956b) | - | Itajaí/SC | sp 26 | *Obama burmeisteri* | -26.957485 | -48.756133 | Brazil |
| Froehlich (1956b) | - | Blumenau/SC | sp 26 | *Obama burmeisteri* | -27.05386 | -49.08606 | Brazil |
| Froehlich (1956b) | - | Brusque/SC | sp 26 | *Obama burmeisteri* | -27.111325 | -48.895427 | Brazil |
| Graff (1899) | - | Morro Reuter/RS | sp 26 | *Obama burmeisteri* | -29.53 | -51.07 | Brazil |
| Marcus (1951) | - | São Paulo/SP | sp 26 | *Obama burmeisteri* | -23.500212 | -46.702310 | Brazil |
| Carbayo et al (2013) | - | São José do Barreiro/SP | sp 27 | *Paraba multicolor* | -22.72583 | -44.62611 | Brazil |
| CG Froehlich (1956a) | - | Teresópolis/RJ | sp 27 | *Paraba multicolor* | -22.439089 | -42.994351 | Brazil |
| **F2646** | External and AC | PE Intervales | sp 27 | *Paraba multicolor* | -24.269387 | -48.405467 | Brazil |
| **F3301** | External, F and AC | PE Tabuleiro | sp 27 | *Paraba multicolor* | -27.840735 | -48.924866 | Brazil |
| **F7317** | External | PN São Joaquim | sp 27 | *Paraba multicolor* | -28.040421 | -49.615316 | Brazil |
| Froehlich (1956b) | - | Curitiba/PR | sp 27 | *Paraba multicolor* | -25.548339 | -49.313845 | Brazil |
| Froehlich (1956b) | - | Lapa/PR | sp 27 | *Paraba multicolor* | -25.834333 | -49.904062 | Brazil |
| Froehlich (1957) | - | Guapiara/SP | sp 27 | *Paraba multicolor* | -24.173828 | -48.542533 | Brazil |
| Froehlich (1957) | - | Ponta Grossa/PR | sp 27 | *Paraba multicolor* | -25.101490 | -50.174131 | Brazil |
| Leal-Zanchet & Matos (2011) | - | Caxias do Sul/RS | sp 27 | *Paraba multicolor* | -29.194212 | -51.165792 | Brazil |
| Leal-Zanchet & Matos (2011) | - | Ibarama/RS | sp 27 | *Paraba multicolor* | -29.722778 | -53.714177 | Brazil |
| Leal-Zanchet & Matos (2011) | - | Santa Maria/RS | sp 27 | *Paraba multicolor* | -29.722778 | -53.714177 | Brazil |
| Marcus (1951) | - | Avaré/SP | sp 27 | *Paraba multicolor* | -23.096206 | -48.914060 | Brazil |
| Marcus (1951) | - | São Paulo/SP | sp 27 | *Paraba multicolor* | -23.453148 | -46.634488 | Brazil |
| Marcus (1951) | - | Mogi das Cruzes/SP | sp 27 | *Paraba multicolor* | -23.505856 | -46.211903 | Brazil |
| Marcus (1951) | - | Santo André/SP | sp 27 | *Paraba multicolor* | -23.773397 | -46.333248 | Brazil |
| **F1619** | External, F and AC | Saint-Hilaire | sp 28 | *Obama polyophthalma* | -25.71 | -48.62 | Brazil |
| Froehlich (1956b) | - | Caiobá/PR | sp 28 | *Obama polyophthalma* | -25.817820 | -48.549932 | Brazil |
| Froehlich (1956b) | - | Rio do Testo/SC | sp 28 | *Obama polyophthalma* | -26.743720 | -49.175644 | Brazil |
| Graff (1899) | - | Joinville/SC | sp 28 | *Obama polyophthalma* | -26.28 | -48.86 | Brazil |
| Graff (1899) | - | Blumenau/SC | sp 28 | *Obama polyophthalma* | -27.05386 | -49.08606 | Brazil |
| Graff (1899) | - | Taquara/RS | sp 28 | *Obama polyophthalma* | -29.659801 | -50.779542 | Brazil |
| Carbayo et al (2013) | - | Ribeirão Grande/SP | sp 29 | *Obama carinata* | -24.26 | -48.40 | Brazil |
| **F3122** | External, A2, A3, F and AC | PE Intervales | sp 29 | *Obama carinata* | -24.269387 | -48.405467 | Brazil |
| Froehlich (1956b) | - | Morretes/PR | sp 29 | *Obama carinata* | -25.483460 | -48.832020 | Brazil |
| Froehlich (1956b) | - | Curitiba/PR | sp 29 | *Obama carinata* | -25.548339 | -49.313845 | Brazil |
| Froehlich (1957) | - | Pirassununga/SP | sp 29 | *Obama carinata* | -21.934693 | -47.372714 | Brazil |
| Froehlich (1957) | - | Itapecerica da Serra/SP | sp 29 | *Obama carinata* | -23.741677 | -46.886010 | Brazil |
| Froehlich (1957) | - | Juquiá/SP | sp 29 | *Obama carinata* | -24.317073 | -47.639867 | Brazil |
| Marcus (1951) | - | São Paulo/SP | sp 29 | *Obama carinata* | -23.453148 | -46.634488 | Brazil |
| Marcus (1951) | - | Mogi das Cruzes/SP | sp 29 | *Obama carinata* | -23.505856 | -46.211903 | Brazil |
| Marcus (1951) | - | Mongaguá/SP | sp 29 | *Obama carinata* | -24.085174 | -46.640241 | Brazil |
| Marcus (1951) | - | Eldorado/SP | sp 29 | *Obama carinata* | -24.498352 | -48.103028 | Brazil |
| Riester (1938) | - | Ribeirão Pires/SP | sp 29 | *Obama carinata* | -23.701329 | -46.39 | Brazil |
| Carbayo et al (2013) | - | São Bernardo/SP | sp 30 | *Pasipha rosea* | -23.77368 | -46.51469 | Brazil |
| EM Froehlich (1955a) | - | Pirassununga/SP | sp 30 | *Pasipha rosea* | -22.007522 | -47.459448 | Brazil |
| EM Froehlich (1955a) | - | São Paulo/SP | sp 30 | *Pasipha rosea* | -23.453148 | -46.634488 | Brazil |
| Froehlich (1956b) | - | Lapa/PR | sp 30 | *Pasipha rosea* | -25.834333 | -49.904062 | Brazil |
| Froehlich (1957) | - | São Simão | sp 30 | *Pasipha rosea* | -21.471847 | -47.573321 | Brazil |
| **F2032** | External and AC | São José do Barreiro/SP | Sp 30 | *Pasipha rosea* | -22.78222 | -44.60583 | Brazil |
| Froehlich (1956b) | - | Morretes/PR | sp 31 | *Cratera joia* | -25.483460 | -48.832020 | Brazil |
| Froehlich (1956b) | - | Morretes/PR | sp 32 | *Notogynaphallia mourei* | -25.483460 | -48.832020 | Brazil |
| Froehlich (1956b) | - | Curitiba/PR | sp 32 | *Notogynaphallia mourei* | -25.548339 | -49.313845 | Brazil |
| Carbayo et al (2013) | - | Ribeirão Grande/SP | sp 33 | *Notogynaphallia plumbea* | -24.27638 | -48.41556 | Brazil |
| **F1627** | External and F | PN Saint Hilaire | sp 33 | *Notogynaphallia plumbea* | -25.469604 | -48.813739 | Brazil |
| **F3085** | External, F and AC | PE Intervales | sp 33 | *Notogynaphallia plumbea* | -24.269387 | -48.405467 | Brazil |
| **F3229** | External, A1, F and AC | PE Tabuleiro | sp 33 | *Notogynaphallia plumbea* | -27.9813889 | -48.748055555 | Brazil |
| Froehlich (1956b) | - | São Paulo/SP | sp 33 | *Notogynaphallia plumbea* | -23.459300 | -46.755164 | Brazil |
| Froehlich (1956b) | - | Morretes/PR | sp 33 | *Notogynaphallia plumbea* | -25.483460 | -48.832020 | Brazil |
| Almeida et al (2018) | - | Alfredo Wagner/SC | sp 34 | *Geoplana pulchella* | -27.61695 | -49.34778 | Brazil |
| Almeida et al (2018) | - | Paulo Lopes/SC | sp 34 | *Geoplana pulchella* | -27.98139 | -48.74806 | Brazil |
| Schultze & Müller (1857) | - | Blumenau/SC | sp 34 | *Geoplana pulchella* | -27.05386 | -49.08606 | Brazil |
| Froehlich (1955b) | - | Teresópolis/RJ | sp 35 | *Pasipha splendida* | -22.439089 | -42.994351 | Brazil |
| Froehlich (1955b) | - | Teresópolis/RJ | sp 36 | *Pasipha oliverioi* | -22.439089 | -42.994351 | Brazil |
| Froehlich (1955b) | - | Teresópolis/RJ | sp 37 | *Geoplana fragai* | -22.439089 | -42.994351 | Brazil |
| Froehlich (1955b) | - | Teresópolis/RJ | sp 38 | *Geoplana jandira* | -22.439089 | -42.994351 | Brazil |
| Froehlich (1957) | - | Amparo/SP | sp 39 | *Obama braunsi* | -22.682420 | -46.732935 | Brazil |
| Graff (1899) | - | São Paulo/SP | sp 39 | *Obama braunsi* | -23.459300 | -46.755164 | Brazil |
| Graff (1899) | - | Santos/SP | sp 39 | *Obama braunsi* | -23.87 | -46.32 | Brazil |
| Marcus (1951) | - | Mogi das Cruzes/SP | sp 39 | *Obama braunsi* | -23.505856 | -46.211903 | Brazil |
| **F4624** | External, F and AC | PN Itajaí | sp 40 | *Paraba caapora* | -27.05386 | -49.08606 | Brazil |
| Froehlich (1957) | - | Apiaí/SP | sp 40 | *Paraba caapora* | -24.518261 | -48.844379 | Brazil |
| Froehlich (1957) | - | Itanhaém/SP | sp 41 | *Obama poca* | -24.148182 | -46.830256 | Brazil |
| Froehlich (1957) | - | São Paulo/SP | sp 42 | *Obama schubarti* | -23.459300 | -46.755164 | Brazil |
| Froehlich (1957) | - | Itanhaém/SP | sp 42 | *Obama schubarti* | -24.148182 | -46.830256 | Brazil |
| **F3797** | External and AC | PE Intervales | sp 43 | *Paraba tapira* | -24.269387 | -48.405467 | Brazil |
| Froehlich (1957) | - | Tapiraí/SP | sp 43 | *Paraba tapira* | -23.964151 | -47.512885 | Brazil |
| Froehlich (1957) | - | Juquiá/SP | sp 44 | *Geoplana toriba* | -24.317073 | -47.639867 | Brazil |
| **F3136** | External and all internal | PE Intervales | sp 45 | *Issoca potyra* | -24.269387 | -48.405467 | Brazil |
| Froehlich (1957) | - | Eldorado/SP | sp 45 | *Issoca potyra* | -24.498352 | -48.103028 | Brazil |
| Amaral et al (2012) | - | Praia Grande/SC | sp 46 | *Obama ficki* | -29.198086 | -50.026672 | Brazil |
| Amaral et al (2012) | - | São Francisco de Paula/RS | sp 46 | *Obama ficki* | -29.428944 | -50.392000 | Brazil |
| Amaral et al (2014) | - | Salvador do Sul/RS | sp 46 | *Obama ficki* | -29.442428 | -51.502151 | Brazil |
| **F7380** | External | PN São Joaquim | sp 46 | *Obama ficki* | -28.235930 | -49.498947 | Brazil |
| **F7258** | External and AC | PN São Joaquim | sp 47 | *Obama* sp. 14 | -28.156986 | -49.637516 | Brazil |
| **F7372** | External and AC | PN São Joaquim | sp 48 | *Cratera* sp. 5 | -28.162100 | -49.626466 | Brazil |
| Riester (1938) | - | Teresópolis/RJ | sp 49 | *Paraba preta* | -22.439089 | -42.994351 | Brazil |
| Carbayo et al (2013) | - | São Leopoldo/RS | sp 50 | *Geobia subterranea* | -29.764171 | -51.155840 | Brazil |
| CG Froehlich (1955) | - | São Paulo/SP | sp 50 | *Geobia subterranea* | -23.448030 | -46.666767 | Brazil |
| **F3608** | External | Saint-Hilaire | sp 50 | *Geobia subterranea* | -25.71 | -48.62 | Brazil |
| Marcus (1951) | - | Avaré/SP | sp 50 | *Geobia subterranea* | -23.096206 | -48.914060 | Brazil |
| Marcus (1951) | - | Mogi das Cruzes/SP | sp 50 | *Geobia subterranea* | -23.505856 | -46.211903 | Brazil |
| Riester (1938) | - | Teresópolis/RJ | sp 50 | *Geobia subterranea* | -22.439089 | -42.994351 | Brazil |
| Schultze & Müller (1857) | - | Blumenau/SC | sp 50 | *Geobia subterranea* | -27.05386 | -49.08606 | Brazil |
| Carbayo et al (2017) | - | Salesópolis/SP | sp 51 | *Choeradoplana marthae* | -23.63 | -45.86 | Brazil |
| CG Froehlich (1955) | - | Mongaguá/SP | sp 51 | *Choeradoplana marthae* | -24.085174 | -46.640241 | Brazil |
| CG Froehlich (1955) | - | Teresópolis/RJ | sp 52 | *Choeradoplana catua* | -22.439089 | -42.994351 | Brazil |
| CG Froehlich (1955) | - | Ubatuba/SP | sp 53 | *Issoca rezendei* | -23.423381 | -45.083399 | Brazil |
| CG Froehlich (1955) | - | Mongaguá/SP | sp 53 | *Issoca rezendei* | -24.085174 | -46.640241 | Brazil |
| CG Froehlich (1955) | - | Itanhaém/SP | sp 53 | *Issoca rezendei* | -24.148182 | -46.830256 | Brazil |
| CG Froehlich (1955) | - | Itajaí/SC | sp 53 | *Issoca rezendei* | -26.957485 | -48.756133 | Brazil |
| CG Froehlich (1955) | - | Blumenau/SC | sp 53 | *Issoca rezendei* | -27.05386 | -49.08606 | Brazil |
| **F3595** | External and AC | Saint-Hilaire | sp 53 | *Issoca rezendei* | -25.469604 | -48.813739 | Brazil |
| Marcus (1951) | - | São Paulo/SP | sp 53 | *Issoca rezendei* | -23.578104 | -46.689043 | Brazil |
| Schirch (1929) | - | Teresópolis/RJ | sp 53 | *Issoca rezendei* | -22.500057 | -42.997365 | Brazil |
| Carbayo et al (2013) | - | São José do Barreiro/SP | sp 54 | *Issoca jandaia* | -22.78222 | -44.60583 | Brazil |
| CG Froehlich (1955) | - | Monte Jaraguá | sp 54 | *Issoca jandaia* | -23.459736 | -46.761403 | Brazil |
| **F2689** | External, f and AC | PE Intervales | sp 54 | *Issoca jandaia* | -24.269387 | -48.405467 | Brazil |
| CG Froehlich (1955) | - | Teresópolis/RJ | sp 55 | *Issoca piranga* | -22.439089 | -42.994351 | Brazil |
| CG Froehlich (1955) | - | São Paulo/SP | sp 56 | *Xerapoa hystrix* | -23.453148 | -46.634488 | Brazil |
| CG Froehlich (1955) | - | Mogi das Cruzes/SP | sp 56 | *Xerapoa hystrix* | -23.505856 | -46.211903 | Brazil |
| CG Froehlich (1955) | - | Curitiba/PR | sp 56 | *Xerapoa hystrix* | -25.548339 | -49.313845 | Brazil |
| CG Froehlich (1955) | - | Brusque/SC | sp 57 | *Xerapoa una* | -27.111325 | -48.895427 | Brazil |
| CG Froehlich (1956a) | - | Teresópolis/RJ | sp 58 | *Obama applanata* | -22.439089 | -42.994351 | Brazil |
| Carbayo et al (2013) | - | São Paulo/SP | sp 59 | *Notogynaphallia sexstriata* | -23.56671 | -46.73017 | Brazil |
| Carbayo et al (2013) | - | Blumenau/SC | sp 59 | *Notogynaphallia sexstriata* | -27.04836 | -49.09200 | Brazil |
| CG Froehlich (1956a) | - | Teresópolis/RJ | sp 59 | *Notogynaphallia sexstriata* | -22.439089 | -42.994351 | Brazil |
| du Bois Reymond-Marcus (1951) | - | Brusque/SC | sp 59 | *Notogynaphallia sexstriata* | -27.111325 | -48.895427 | Brazil |
| du Bois Reymond-Marcus (1951) | - | Taquara/RS | sp 59 | *Notogynaphallia sexstriata* | -29.659801 | -50.779542 | Brazil |
| **F1413** | External, F and AC | PNItajai | sp 59 | *Notogynaphallia sexstriata* | -27.05306 | -49.08528 | Brazil |
| **F1741** | External, A1 and AC | Saint-Hilaire | sp 59 | *Notogynaphallia sexstriata* | -25.469604 | -48.813739 | Brazil |
| Schirch (1929) | - | Teresópolis/RJ | sp 60 | *Paraba goettei* | -22.439089 | -42.994351 | Brazil |
| Schirch (1929) | - | Teresópolis/RJ | sp 61 | *Pasipha plana* | -22.439089 | -42.994351 | Brazil |
| Riester (1938) | - | Teresópolis/RJ | sp 62 | *Cratera pseudovaginuloides* | -22.439089 | -42.994351 | Brazil |
| Carbayo (2010) | - | Ribeirão Pires/SP | sp 63 | *Luteostriata caissara* | -23.701329 | -46.39 | Brazil |
| Carbayo (2010) | - | Itanhaém/SP | sp 63 | *Luteostriata caissara* | -24.148182 | -46.830256 | Brazil |
| CG Froehlich (1956a) | - | Teresópolis/RJ | sp 63 | *Luteostriata caissara* | -22.439089 | -42.994351 | Brazil |
| CG Froehlich (1956a) | - | Teresópolis/RJ | sp 63 | *Luteostriata caissara* | -22.500057 | -42.997365 | Brazil |
| CG Froehlich (1956a) | - | Rio de Janeiro/RJ | sp 63 | *Luteostriata caissara* | -22.975852 | -43.292230 | Brazil |
| CG Froehlich (1956a) | - | Ubatuba/SP | sp 63 | *Luteostriata caissara* | -23.423381 | -45.083399 | Brazil |
| CG Froehlich (1956a) | - | Teresópolis/RJ | sp 64 | *Paraba cassula* | -22.439089 | -42.994351 | Brazil |
| Carbayo et al (2017) | - | Santa Maria Madalena/RJ | sp 65 | *Matuxia matuta* | -21.87 | -41.91 | Brazil |
| CG Froehlich (1956a) | - | Teresópolis/RJ | sp 65 | *Matuxia matuta* | -22.439089 | -42.994351 | Brazil |
| Carbayo et al (2016) | - | Teresópolis/RJ | sp 66 | *Cratera tamoia* | -22.46447 | -43.00278 | Brazil |
| CG Froehlich (1956a) | - | Teresópolis/RJ | sp 66 | *Cratera tamoia* | -22.439089 | -42.994351 | Brazil |
| CG Froehlich (1956a) | - | Teresópolis/RJ | sp 66 | *Cratera tamoia* | -22.500057 | -42.997365 | Brazil |
| CG Froehlich (1956a) | - | Teresópolis/RJ | sp 67 | *Obama trigueira* | -22.439089 | -42.994351 | Brazil |
| CG Froehlich (1956a) | - | Teresópolis/RJ | sp 67 | *Obama trigueira* | -22.500057 | -42.997365 | Brazil |
| CG Froehlich (1956a) | - | Teresópolis/RJ | sp 68 | *Cratera yara* | -22.439089 | -42.994351 | Brazil |
| **F6897** | External and all internal | Saint-Hilaire | sp 69 | *Pasipha* sp. 7 | -25.469604 | -48.813739 | Brazil |
| CG Froehlich (1956a) | - | Teresópolis/RJ | sp 70 | *Rhynchodemus hectori* | -22.439089 | -42.994351 | Brazil |
| CG Froehlich (1956a) | - | Rio de Janeiro/RJ | sp 71 | *Barreira barreirana* | -22.975852 | -43.292230 | Brazil |
| CG Froehlich (1956a) | - | Ubatuba/SP | sp 71 | *Barreira barreirana* | -23.423381 | -45.083399 | Brazil |
| Riester (1938) | - | Teresópolis/RJ | sp 71 | *Barreira barreirana* | -22.500057 | -42.997365 | Brazil |
| Riester (1938) | - | Teresópolis/RJ | sp 72 | *Barreira zebroides* | -22.500057 | -42.997365 | Brazil |
| Marcus (1951) | - | São Paulo/SP | sp 73 | *Cratera taxiarcha* | -23.453148 | -46.634488 | Brazil |
| CG Froehlich (1956a) | - | Ubatuba/SP | sp 74 | *Pasipha cafusa* | -23.423381 | -45.083399 | Brazil |
| CG Froehlich (1956a) | - | Ubatuba/SP | sp 75 | *Geoplana picta* | -23.423381 | -45.083399 | Brazil |
| CG Froehlich (1956a) | - | Ubatuba/SP | sp 76 | *Rhynchodemus scius* | -23.423381 | -45.083399 | Brazil |
| Carbayo et al (2013) | - | São José do Barreiro/SP | sp 272 | *Pasipha* sp. 15 | -22.73389 | -44.61639 | Brazil |
| EM Froehlich (1955a) | - | São Paulo/SP | sp 77 | *Pasipha chimbeva* | -23.459736 | -46.761403 | Brazil |
| EM Froehlich (1955a) | - | Pirassununga/SP | sp 78 | *Geoplana chiuna* | -22.007522 | -47.459448 | Brazil |
| EM Froehlich (1955a) | - | São Paulo/SP | sp 79 | *Cratera crioula* | -23.453148 | -46.634488 | Brazil |
| EM Froehlich (1955a) | - | São Paulo/SP | sp 80 | *Obama livia* | -23.453148 | -46.634488 | Brazil |
| EM Froehlich (1955a) | - | São Paulo/SP | sp 80 | *Obama livia* | -23.459736 | -46.761403 | Brazil |
| EM Froehlich (1955a) | - | São Paulo/SP | sp 81 | *Notogynaphallia parca* | -23.453148 | -46.634488 | Brazil |
| Carbayo et al (2013) | - | Teresópolis/RJ | sp 82 | *Pasipha pinima* | -22.45528 | -42.99750 | Brazil |
| Carbayo et al (2013) | - | Morretes/PR | sp 82 | *Pasipha pinima* | -25.46972 | -48.81222 | Brazil |
| EM Froehlich (1955a) | - | São Paulo/SP | sp 82 | *Pasipha pinima* | -23.453148 | -46.634488 | Brazil |
| **F2752** | External and AC | PE Intervales | sp 82 | *Pasipha pinima* | -24.269387 | -48.405467 | Brazil |
| **F7296** | External | PN São Joaquim | sp 82 | *Pasipha pinima* | -28.041154 | -49.615693 | Brazil |
| EM Froehlich (1955a) | - | Araraquara/SP | sp 83 | *Geoplana regia* | -21.779764 | -48.161178 | Brazil |
| Carbayo et al (2013) | - | Teresópolis/RJ | sp 84 | *Matuxia tuxaua* | -22.45528 | -42.99750 | Brazil |
| EM Froehlich (1955a) | - | São Paulo/SP | sp 84 | *Matuxia tuxaua* | -23.453148 | -46.634488 | Brazil |
| EM Froehlich (1955a) | - | São Paulo/SP | sp 84 | *Matuxia tuxaua* | -23.459736 | -46.761403 | Brazil |
| Marcus (1951) | - | São Paulo/SP | sp 85 | *Geoplana ferussaci sensu Riester, 1938* | -23.500212 | -46.702310 | Brazil |
| Riester (1938) | - | Nova Lima/MG | sp 85 | *Geoplana ferussaci sensu Riester, 1938* | -20.02 | -43.91 | Brazil |
| Riester (1938) | - | Teresópolis/RJ | sp 85 | *Geoplana ferussaci sensu Riester, 1938* | -22.500057 | -42.997365 | Brazil |
| Marcus (1951) | - | São Paulo/SP | sp 86 | *Obama metzi* | -23.500212 | -46.702310 | Brazil |
| Riester (1938) | - | Ribeirão Pires/SP | sp 86 | *Obama metzi* | -23.701329 | -46.39 | Brazil |
| Álvarez-PResas et al (2011) | - | São José do Barreiro/SP | sp 87 | *Imbira marcusi* | -22.72583 | -44.62611 | Brazil |
| **F3161** | External, F and AC | PE Intervales | sp 87 | *Imbira marcusi* | -24.269387 | -48.405467 | Brazil |
| **F3568** | External and all internal | PN Saint Hilaire | sp 87 | *Imbira marcusi* | -25.469604 | -48.813739 | Brazil |
| Marcus (1951) | - | São Paulo/SP | sp 87 | *Imbira marcusi* | -23.500212 | -46.702310 | Brazil |
| Marcus (1951) | - | Mogi das Cruzes/SP | sp 87 | *Imbira marcusi* | -23.505856 | -46.211903 | Brazil |
| Marcus (1951) | - | Mongaguá/SP | sp 87 | *Imbira marcusi* | -24.085174 | -46.640241 | Brazil |
| Marcus (1951) | - | São Paulo/SP | sp 88 | *Pasipha astraea* | -23.500212 | -46.702310 | Brazil |
| **F3084** | External and AC | PE Intervales | sp 89 | *Obama divae* | -24.269387 | -48.405467 | Brazil |
| Marcus (1951) | - | Santo André/SP | sp 89 | *Obama divae* | -23.773397 | -46.333248 | Brazil |
| Marcus (1951) | - | São Paulo/SP | sp 90 | *Geoplana duca* | -23.453148 | -46.634488 | Brazil |
| Marcus (1951) | - | Mogi das Cruzes/SP | sp 90 | *Geoplana duca* | -23.505856 | -46.211903 | Brazil |
| Marcus (1951) | - | Santo André/SP | sp 91 | *Obama evelinae* | -23.773397 | -46.333248 | Brazil |
| Marcus (1951) | - | Mongaguá/SP | sp 91 | *Obama evelinae* | -24.085174 | -46.640241 | Brazil |
| Marcus (1951) | - | Santo André/SP | sp 92 | *Cratera hina* | -23.773397 | -46.333248 | Brazil |
| Marcus (1951) | - | São Paulo/SP | sp 93 | *Pasipha pasipha* | -23.453148 | -46.634488 | Brazil |
| Marcus (1951) | - | Mogi das Cruzes/SP | sp 94 | *Geoplana pavani* | -23.505856 | -46.211903 | Brazil |
| Carbayo et al (2013) | - | São José do Barreiro/SP | sp 271 | *Paraba* sp. 7 | -22.73389 | -44.61639 | Brazil |
| Marcus (1951) | - | São Paulo/SP | sp 95 | *Paraba phocaica* | -23.500212 | -46.702310 | Brazil |
| Carbayo et al (2013) | - | Teresópolis/RJ | sp 96 | *Xerapoa trina* | -22.38455 | -42.97681 | Brazil |
| Carbayo et al (2013) | - | Teresópolis/RJ | sp 96 | *Xerapoa trina* | -22.45528 | -42.99750 | Brazil |
| Marcus (1951) | - | Santo André/SP | sp 96 | *Xerapoa trina* | -23.773397 | -46.333248 | Brazil |
| Marcus (1951) | - | Angra dos Reis/RJ | sp 97 | *Choeradoplana bilix* | -22.946008 | -44.474647 | Brazil |
| Amaral et al (2012) | - | Derrubadas/RS | sp 98 | *Obama carbayoi* | -27.247305 | -53.893789 | Brazil |
| Amaral et al (2012) | - | Santa Maria/RS | sp 99 | *Obama baptistae* | -29.722778 | -53.714177 | Brazil |
| Amaral & Leal-Zanchet (2016) | - | Santa Maria/RS | sp 100 | *Pasipha mesoxantha* | -29.722778 | -53.714177 | Brazil |
| Amaral & Leal-Zanchet (2016) | - | Derrubadas/RS | sp 101 | *Pasipha turvensis* | -27.247305 | -53.893789 | Brazil |
| Carbayo (2010) | - | Pirassununga/SP | sp 102 | *Luteostriata ernesti* | -22.007522 | -47.459448 | Brazil |
| Carbayo (2010) | - | Mogi das Cruzes/SP | sp 102 | *Luteostriata ernesti* | -23.505856 | -46.211903 | Brazil |
| Carbayo (2010) | - | Ibiúna/SP | sp 102 | *Luteostriata ernesti* | -23.75 | -47.19 | Brazil |
| **F3222** | External | PE Tabuleiro | sp 102 | *Luteostriata ernesti* | -27.94 | -48.79 | Brazil |
| Leal-Zanchet & Froehlich (2006) | - | Valinhos/SP | sp 102 | *Luteostriata ernesti* | -22.96 | -46.98 | Brazil |
| Leal-Zanchet & Froehlich (2006) | - | Jundiaí/SP | sp 102 | *Luteostriata ernesti* | -23.21 | -46.95 | Brazil |
| Leal-Zanchet & Froehlich (2006) | - | São Paulo/SP | sp 102 | *Luteostriata ernesti* | -23.453148 | -46.634488 | Brazil |
| Leal-Zanchet & Froehlich (2006) | - | Ribeirão Pires/SP | sp 102 | *Luteostriata ernesti* | -23.701329 | -46.39 | Brazil |
| Leal-Zanchet & Froehlich (2006) | - | Curitiba/PR | sp 102 | *Luteostriata ernesti* | -25.548339 | -49.313845 | Brazil |
| Leal-Zanchet & Froehlich (2006) | - | São Francisco de Paula/RS | sp 102 | *Luteostriata ernesti* | -29.428944 | -50.392000 | Brazil |
| Baptista et al (2006) | - | Cambará do Sul/RS | sp 103 | *Obama josefi* | -29.179104 | -50.080631 | Brazil |
| Carbayo & Leal-Zanchet (2001) | - | São Francisco de Paula/RS | sp 103 | *Obama josefi* | -29.428944 | -50.392000 | Brazil |
| Leal-Zanchet et al (2011) | - | São Francisco de Paula/RS | sp 103 | *Obama josefi* | -29.179104 | -50.080631 | Brazil |
| Leal-Zanchet et al (2011) | - | São Francisco de Paula/RS | sp 103 | *Obama josefi* | -29.480590 | -50.174679 | Brazil |
| Almeida et al (2018) | - | Florianópolis/SC | sp 104 | *Luteostriata graffi* | -27.468485 | -48.377724 | Brazil |
| Antunes et al (2012) | - | São Francisco de Paula/RS | sp 104 | *Luteostriata graffi* | -29.428944 | -50.392000 | Brazil |
| Baptista et al (2006) | - | Cambará do Sul/RS | sp 104 | *Luteostriata graffi* | -29.179104 | -50.080631 | Brazil |
| Carbayo (2010) | - | Morro Reuter/RS | sp 104 | *Luteostriata graffi* | -29.53 | -51.07 | Brazil |
| Carbayo (2010) | - | Parobé/RS | sp 104 | *Luteostriata graffi* | -29.62940 | -50.83110 | Brazil |
| Leal-Zanchet & Froehlich (2006) | - | Salvador do Sul/RS | sp 104 | *Luteostriata graffi* | -29.442428 | -51.502151 | Brazil |
| Leal-Zanchet & Froehlich (2006) | - | Três Coroas/RS | sp 104 | *Luteostriata graffi* | -29.49 | -50.82 | Brazil |
| Leal-Zanchet & Froehlich (2006) | - | Taquara/RS | sp 104 | *Luteostriata graffi* | -29.659801 | -50.779542 | Brazil |
| Leal-Zanchet et al (2011) | - | São Francisco de Paula/RS | sp 104 | *Luteostriata graffi* | -29.480590 | -50.174679 | Brazil |
| Carbayo & Leal-Zanchet (2003) | - | São Francisco de Paula/RS | sp 105 | *Supramontana irritata* | -29.428944 | -50.392000 | Brazil |
| Baptista & Leal-Zanchet (2005) | - | São Francisco de Paula/RS | sp 106 | *Paraba rubidolineata* | -29.428944 | -50.392000 | Brazil |
| Baptista et al (2010) | - | Derrubadas/RS | sp 106 | *Paraba rubidolineata* | -27.247305 | -53.893789 | Brazil |
| **F3271** | External | PE Tabuleiro | sp 106 | *Paraba rubidolineata* | -27.840735 | -48.924866 | Brazil |
| Baptista et al (2006) | - | Cambará do Sul/RS | sp 107 | *Paraba franciscana* | -29.179104 | -50.080631 | Brazil |
| Leal-Zanchet & Carbayo (2001) | - | São Francisco de Paula/RS | sp 107 | *Paraba franciscana* | -29.428944 | -50.392000 | Brazil |
| Leal-Zanchet et al (2011) | - | São Francisco de Paula/RS | sp 107 | *Paraba franciscana* | -29.480590 | -50.174679 | Brazil |
| Leal-Zanchet et al (2011) | - | Cambará do Sul/RS | sp 108 | *Luteostriata arturi* | -29.179104 | -50.080631 | Brazil |
| Lemos & Leal-Zanchet (2008) | - | São Francisco de Paula/RS | sp 108 | *Luteostriata arturi* | -29.428944 | -50.392000 | Brazil |
| Leal-Zanchet et al (2012) | - | São Francisco de Paula/RS | sp 109 | *Pasipha backesi* | -29.428944 | -50.392000 | Brazil |
| Leal-Zanchet et al (2012) | - | São Francisco de Paula/RS | sp 110 | *Pasipha brevilineata* | -29.428944 | -50.392000 | Brazil |
| Lemos & Leal-Zanchet (2008) | - | São Francisco de Paula/RS | sp 111 | *Luteostriata pseudoceciliae* | -29.428944 | -50.392000 | Brazil |
| du Bois Reymond-Marcus (1951) | - | Brusque/SC | sp 112 | *Choeradoplana langi* | -27.111325 | -48.895427 | Brazil |
| Graff (1899) | - | Blumenau/SC | sp 112 | *Choeradoplana langi* | -27.05386 | -49.08606 | Brazil |
| **F3590** | External, F and AC | Saint-Hilaire | sp 113 | *Cratera* sp. 3 | -25.469604 | -48.813739 | Brazil |
| Hyman (1955) | - | São Bento do Sul/SC | sp 114 | *Obama catharina* | -26.23 | -49.40 | Brazil |
| Rossi et al (2014) | - | Cambará do Sul/RS | sp 115 | *Cratera steffeni* | -29.179104 | -50.080631 | Brazil |
| **F7267** | External | PN São Joaquim | sp 116 | *Cratera ochra* | -28.157565 | -49.638002 | Brazil |
| Rossi et al (2016) | - | São Francisco de Paula/RS | sp 116 | *Cratera ochra* | -29.428944 | -50.392000 | Brazil |
| Rossi et al (2016) | - | Praia Grande/SC | sp 117 | *Obama maculipunctata* | -29.198086 | -50.026672 | Brazil |
| Rossi et al (2016) | - | São Francisco de Paula/RS | sp 117 | *Obama maculipunctata* | -29.428944 | -50.392000 | Brazil |
| Rossi & Leal-Zanchet (2017) | - | General Carneiro/PR | sp 118 | *Cratera nigrimarginata* | -26.44 | -51.31 | Brazil |
| Rossi & Leal-Zanchet (2017) | - | Três Barras/SC | sp 119 | *Cratera cryptolineata* | -26.19 | -50.33 | Brazil |
| Rossi & Leal-Zanchet (2017) | - | Três Barras/SC | sp 120 | *Cratera aureomaculata* | -26.19 | -50.33 | Brazil |
| Schirch (1929) | - | Resende/RJ | sp 121 | *Obama itatiayana* | -22.38 | -44.68 | Brazil |
| Graff (1899) | - | Morro Reuter/RS | sp 122 | *Notogynaphallia octostriata* | -29.53 | -51.07 | Brazil |
| Schultze & Müller (1857) | - | Blumenau/SC | sp 122 | *Notogynaphallia octostriata* | -27.05386 | -49.08606 | Brazil |
| Carbayo et al (2017) | - | Santa Teresa/ES | sp 123 | *Choeradoplana albonigra* | -19.90 | -40.54 | Brazil |
| Carbayo et al (2017) | - | Santa Maria Madalena/RJ | sp 123 | *Choeradoplana albonigra* | -21.90 | -41.95 | Brazil |
| Riester (1938) | - | Teresópolis/RJ | sp 123 | *Choeradoplana albonigra* | -22.439089 | -42.994351 | Brazil |
| Riester (1938) | - | Teresópolis/RJ | sp 124 | *Pasipha biseminalis* | -22.439089 | -42.994351 | Brazil |
| **F3582** | External and all internal | Saint-Hilaire | sp 125 | *Cratera* sp. 2 | -25.469604 | -48.813739 | Brazil |
| Riester (1938) | - | Teresópolis/RJ | sp 126 | *Pasipha caeruleonigra* | -22.439089 | -42.994351 | Brazil |
| Riester (1938) | - | Teresópolis/RJ | sp 127 | *Geoplana goetschi* | -22.439089 | -42.994351 | Brazil |
| **F1637** | External, F and AC | Saint-Hilaire | sp 128 | *Obama* sp. 5 | -25.469604 | -48.813739 | Brazil |
| Riester (1938) | - | Nova Lima/MG | sp 129 | *Pasipha velutina* | -20.02 | -43.91 | Brazil |
| Riester (1938) | - | Teresópolis/RJ | sp 130 | *Geoplana incognita* | -22.439089 | -42.994351 | Brazil |
| Riester (1938) | - | Teresópolis/RJ | sp 131 | *Obama eudoxiae* | -22.439089 | -42.994351 | Brazil |
| Riester (1938) | - | Teresópolis/RJ | sp 132 | *Obama dictyonota* | -22.439089 | -42.994351 | Brazil |
| Riester (1938) | - | Nova Lima/MG | sp 133 | *Geoplana notophthalma* | -20.02 | -43.91 | Brazil |
| Riester (1938) | - | Teresópolis/RJ | sp 133 | *Geoplana notophthalma* | -22.439089 | -42.994351 | Brazil |
| Riester (1938) | - | Teresópolis/RJ | sp 134 | *Obama eudoximariae* | -22.500057 | -42.997365 | Brazil |
| Riester (1938) | - | Teresópolis/RJ | sp 135 | *Obama riesteri* | -22.500057 | -42.997365 | Brazil |
| Graff (1899) | - | Rio de Janeiro/RJ | sp 136 | *Obama fryi* | -22.975852 | -43.292230 | Brazil |
| Riester (1938) | - | Teresópolis/RJ | sp 136 | *Obama fryi* | -22.500057 | -42.997365 | Brazil |
| Negrete & Brusa (2014) | - | San Antonio/Arg | sp 137 | *Choeradoplana crassiphalla* | -26.132969 | -53.716113 | Argentina |
| **F2694** | External, A1, F and AC | PE Intervales | sp 138 | *Cratera* sp. 1 | -24.269387 | -48.405467 | Brazil |
| Negrete & Brusa (2016) | - | San Antonio/Arg | sp 139 | *Cratera viridimaculata* | -26.132969 | -53.716113 | Argentina |
| Negrete et al (2014) | - | San Antonio/Arg | sp 140 | *Supramontana argentina* | -25.638105 | -54.324086 | Argentina |
| Negrete et al (2014) | - | San Antonio/Arg | sp 140 | *Supramontana argentina* | -26.132969 | -53.716113 | Argentina |
| Negrete & Brusa (2016) | - | San Antonio/Arg | sp 141 | *Pasipha atla* | -26.132969 | -53.716113 | Argentina |
| Negrete & Brusa (2016) | - | San Antonio/Arg | sp 142 | *Pasipha johnsoni* | -26.132969 | -53.716113 | Argentina |
| Negrete & Brusa (2016) | - | San Antonio/Arg | sp 143 | *Pasipha mbya* | -25.717567 | -54.59656 | Argentina |
| Negrete & Brusa (2016) | - | San Antonio/Arg | sp 143 | *Pasipha mbya* | -26.132969 | -53.716113 | Argentina |
| Carbayo & Froehlich (2012) | - | São Paulo/SP | sp 144 | *Choeradoplana banga* | -23.45 | -46.63 | Brazil |
| Carbayo et al (2017) | - | Campos do Jordão/SP | sp 144 | *Choeradoplana banga* | -22.70 | -45.52 | Brazil |
| Carbayo et al (2017) | - | São Francisco de Paula/RS | sp 145 | *Choeradoplana benyai* | -29.42 | -50.39 | Brazil |
| **F3813** | External, A1, F and AC | PE Tabuleiro | sp 145 | *Choeradoplana benyai* | -27.86 | -48.91 | Brazil |
| Carbayo & Froehlich (2012) | - | São José do Barreiro/SP | sp 146 | *Choeradoplana bocaina* | -22.75 | -44.62 | Brazil |
| Carbayo & Froehlich (2012) | - | Ribeirão Grande/SP | sp 147 | *Choeradoplana gladismariae* | -24.26 | -48.40 | Brazil |
| Carbayo et al (2016) | - | Santo André/SP | sp 148 | *Obama otavioi* | -23.77637 | -46.31195 | Brazil |
| Carbayo & Almeida (2015) | - | Santa Maria Madalena/RJ | sp 149 | *Cratera cuarassu* | -21.87 | -41.91 | Brazil |
| Carbayo & Almeida (2015) | - | Teresópolis/RJ | sp 150 | *Cratera anamariae* | -22.500057 | -42.997365 | Brazil |
| Almeida et al (2012) | - | Teresópolis/RJ | sp 151 | *Paraba piriana* | -22.45528 | -42.99750 | Brazil |
| Almeida et al (2012) | - | Paulo Lopes/SC | sp 152 | *Paraba tingauna* | -27.865111 | -48.919389 | Brazil |
| Almeida et al (2012) | - | Paulo Lopes/SC | sp 152 | *Paraba tingauna* | -27.94944 | -48.79556 | Brazil |
| Carbayo et al (2013) | - | São Francisco de Paula/RS | sp 153 | *Cephaloflexa araucariana* | -29.42736 | -50.39838 | Brazil |
| Carbayo et al (2013) | - | Matinhos/PR | sp 154 | *Cratera picuia* | -25.76437 | -48.62266 | Brazil |
| Amaral et al (2014) | - | Salvador do Sul/RS | sp 155 | *Imbira guaiana* | -29.442428 | -51.502151 | Brazil |
| **F7325** | External | PN São Joaquim | sp 155 | *Imbira guaiana* | -28.039273 | -49.614560 | Brazil |
| Leal-Zanchet & Carbayo (2001) | - | São Francisco de Paula/RS | sp 155 | *Imbira guaiana* | -29.41666 | -50.38333 | Brazil |
| Graff (1899) | - | Blumenau/SC | sp 156 | *Choeradoplana spatulata* | -27.04836 | -49.09200 | Brazil |
| Graff (1899) | - | Blumenau/SC | sp 157 | *Rhynchodemus blainvillei* | -27.04836 | -49.09200 | Brazil |
| Graff (1899) | - | Blumenau/SC | sp 158 | *Anisorhynchodemus pellucidus* | -27.04836 | -49.09200 | Brazil |
| Graff (1899) | - | Joinville/SC | sp 159 | *Choeradoplana ehrenreichi* | -26.28 | -48.86 | Brazil |
| Graff (1899) | - | Nova Friburgo/RJ | sp 160 | *Obama argus* | -22.26 | -42.56 | Brazil |
| Carbayo et al (2018) | - | São Francisco de Paula/RS | sp 161 | *Choeradoplana iheringi* | -29.427361 | -50.398389 | Brazil |
| Carbayo et al (2018) | - | Parobé/RS | sp 161 | *Choeradoplana iheringi* | -29.63 | -50.87 | Brazil |
| Graff (1899) | - | Taquara/RS | sp 161 | *Choeradoplana iheringi* | -29.659801 | -50.779542 | Brazil |
| Almeida et al (2018) | - | Teresópolis/RJ | sp 162 | *Choeradoplana pucupucu* | -22.439089 | -42.994351 | Brazil |
| Almeida et al (2018) | - | São José do Barreiro/SP | sp 162 | *Choeradoplana pucupucu* | -22.74 | -44.61 | Brazil |
| Almeida et al (2018) | - | Ribeirão Pires/SP | sp 162 | *Choeradoplana pucupucu* | -23.701329 | -46.39 | Brazil |
| Almeida et al (2018) | - | São Bonifácio/SC | sp 163 | *Choeradoplana abaiba* | -27.84 | -48.92 | Brazil |
| Almeida et al (2018) | - | Paulo Lopes/SC | sp 163 | *Choeradoplana abaiba* | -27.947500 | -48.751111 | Brazil |
| Almeida et al (2018) | - | Paulo Lopes/SC | sp 163 | *Choeradoplana abaiba* | -27.961389 | -48.758333 | Brazil |
| Almeida et al (2018) | - | Santa Maria Madalena/RJ | sp 164 | *Choeradoplana agua* | -21.876667 | -41.919722 | Brazil |
| Álvarez-Presas et al (2015) | - | São Paulo/SP | sp 165 | *Obama anthropophila* | -23.50 | -46.54 | Brazil |
| Álvarez-Presas et al (2015) | - | Blumenau/SC | sp 165 | *Obama anthropophila* | -27.05386 | -49.08606 | Brazil |
| Álvarez-Presas et al (2015) | - | São Bonifácio/SC | sp 165 | *Obama anthropophila* | -27.84 | -48.92 | Brazil |
| Álvarez-Presas et al (2015) | - | São Francisco de Paula/RS | sp 165 | *Obama anthropophila* | -29.42964 | -50.39783 | Brazil |
| Álvarez-Presas et al (2015) | - | Salvador do Sul/RS | sp 165 | *Obama anthropophila* | -29.440556 | -51.507778 | Brazil |
| Álvarez-Presas et al (2015) | - | Ivoti/RS | sp 165 | *Obama anthropophila* | -29.610000 | -51.163611 | Brazil |
| Álvarez-Presas et al (2015) | - | Taquara/RS | sp 165 | *Obama anthropophila* | -29.659801 | -50.779542 | Brazil |
| Álvarez-Presas et al (2015) | - | Portão/RS | sp 165 | *Obama anthropophila* | -29.69 | -51.27 | Brazil |
| Álvarez-Presas et al (2015) | - | Novo Hamburgo/RS | sp 165 | *Obama anthropophila* | -29.72 | -51.12 | Brazil |
| Carbayo et al (2016) | - | Ribeirão Grande/SP | sp 165 | *Obama anthropophila* | -24.26 | -48.40 | Brazil |
| Carbayo et al (2016) | - | Paulo Lopes/SC | sp 165 | *Obama anthropophila* | -27.84278 | -48.92583 | Brazil |
| **F1414** | External, F and AC | PN Itajaí | sp 165 | *Obama anthropophila* | -27.04836 | -49.09206 | Brazil |
| **F2647** | External and AC | PE Intervales | sp 165 | *Obama anthropophila* | -24.269387 | -48.405467 | Brazil |
| **F3346** | External, F and AC | PE Tabuleiro | sp 165 | *Obama anthropophila* | -27.840735 | -48.924866 | Brazil |
| **F3360** | External | FLONA-SFP | sp 165 | *Obama anthropophila* | -29.428944 | -50.392000 | Brazil |
| Carbayo et al (2016) | - | Santa Maria/RS | sp 166 | *Obama decidualis* | -29.74167 | -53.84417 | Brazil |
| Carbayo et al (2016) | - | Paulo Lopes/SC | sp 167 | *Obama nungara* | -27.84278 | -48.92583 | Brazil |
| **F7439** | External | PN São Joaquim | sp 167 | *Obama nungara* | -27.959777 | -49.482612 | Brazil |
| Almeida et al (2018) | - | São Sebastião/SP | sp 168 | *Geoplana boraceia* | -23.75203 | -45.631027 | Brazil |
| Almeida et al (2018) | - | Salesópolis/SP | sp 168 | *Geoplana boraceia* | -23.75203 | -45.631027 | Brazil |
| Almeida et al (2018) | - | Morretes/PR | sp 169 | *Geoplana chita* | -25.469604 | -48.813739 | Brazil |
| Almeida et al (2018) | - | Matinhos/PR | sp 170 | *Geoplana cambara* | -25.71 | -48.62 | Brazil |
| Almeida et al (2018) | - | Matinhos/PR | sp 170 | *Geoplana cambara* | -25.76437 | -48.62266 | Brazil |
| Almeida et al (2018) | - | Cananeia/SP | sp 171 | *Geoplana cananeia* | -25.091235 | -47.928957 | Brazil |
| Almeida et al (2018) | - | Caraguatatuba/SP | sp 172 | *Geoplana caraguatatuba* | -23.594012 | -45.429672 | Brazil |
| Almeida et al (2018) | - | Santana de Parnaiba/SP | sp 173 | *Geoplana ibiuna* | -23.449229 | -47.012175 | Brazil |
| Almeida et al (2018) | - | Ibiúna/SP | sp 173 | *Geoplana ibiuna* | -23.93 | -47.19 | Brazil |
| Almeida et al (2018) | - | Ribeirão Grande/SP | sp 174 | *Geoplana iporanga* | -24.269387 | -48.405467 | Brazil |
| **F3166** | External and all internal | PE Intervales | sp 174 | *Geoplana iporanga* | -24.269387 | -48.405467 | Brazil |
| Almeida et al (2018) | - | Mogi das Cruzes/SP | sp 175 | *Geoplana mogi* | -23.451536 | -46.227993 | Brazil |
| Almeida et al (2018) | - | São Paulo/SP | sp 175 | *Geoplana mogi* | -23.570942 | -46.693776 | Brazil |
| Almeida et al (2018) | - | Santo André/SP | sp 176 | *Geoplana paranapiacaba* | -23.77746 | -46.30145 | Brazil |
| Almeida et al (2018) | - | São Paulo/SP | sp 177 | *Geoplana piratininga* | -23.490918 | -46.516424 | Brazil |
| Darwin (1844) | - | Rio de Janeiro/RJ | sp 178 | *Geoplana vaginuloides* | -22.93707 | -43.46098 | Brazil |
| Riester (1938) | - | Teresópolis/RJ | sp 178 | *Geoplana vaginuloides* | -22.439089 | -42.994351 | Brazil |
| Almeida et al (2018) | - | Nova Iguaçu/RJ | sp 179 | *Geoplana apua* | -22.71 | -43.56 | Brazil |
| Almeida et al (2018) | - | Santa Teresa/ES | sp 180 | *Issoca assanga* | -19.88740 | -40.54319 | Brazil |
| Almeida et al (2018) | - | Santa Maria Madalena/RJ | sp 180 | *Issoca assanga* | -21.87694 | -41.92583 | Brazil |
| **F2751** | External, F and AC | PE Intervales | sp 181 | *Choeradoplana* sp. 8 | -24.269387 | -48.405467 | Brazil |
| **F2638** | External and AC | PE Intervales | sp 182 | *Geoplana* sp. 9 | -24.269387 | -48.405467 | Brazil |
| **F3804** | External and AC | PE Intervales | sp 183 | Geoplaninae 7 | -24.269387 | -48.405467 | Brazil |
| **F3764** | External, A1, F and AC | PE Intervales | sp 184 | *Issoca* sp. 2 | -24.269387 | -48.405467 | Brazil |
| **F3758** | External and AC | PE Intervales | sp 185 | *Luteostriata* sp. 1 | -24.269387 | -48.405467 | Brazil |
| **F1715** | External, F and AC | PN Saint Hilaire | sp 186 | *Obama* sp. 8 | -25.469604 | -48.813739 | Brazil |
| **F3099** | External and AC | PE Intervales | sp 186 | *Obama* sp. 8 | -24.269387 | -48.405467 | Brazil |
| **F3760** | External and AC | PE Intervales | sp 187 | *Obama* sp. 17 | -24.269387 | -48.405467 | Brazil |
| **F3754c** | External and AC | PE Intervales | sp 188 | *Obama* sp. 24 | -24.269387 | -48.405467 | Brazil |
| **F3768** | External and AC | PE Intervales | sp 189 | *Paraba* sp. 3 | -24.269387 | -48.405467 | Brazil |
| **F2692** | External , F and AC | PE Intervales | sp 190 | *Paraba* sp. 6 | -24.269387 | -48.405467 | Brazil |
| **F3754d** | External, F and AC | PE Intervales | sp 191 | *Paraba* sp. 9 | -24.269387 | -48.405467 | Brazil |
| **F3102** | External, F and AC | PE Intervales | sp 192 | *Pasipha* sp. 8 | -24.269387 | -48.405467 | Brazil |
| **F3239** | External, A1, F and AC | PE Tabuleiro | sp 192 | *Pasipha* sp. 8 | -27.840735 | -48.924866 | Brazil |
| **F3265** | External | PE Tabuleiro | sp 192 | *Pasipha* sp. 8 | -27.994964 | -48.759313 | Brazil |
| **F6511** | External | Alfredo Wagner/SC | sp 192 | *Pasipha* sp. 8 | -27.61727 | -49.35075 | Brazil |
| **F7416** | External | PN São Joaquim | sp 192 | *Pasipha* sp. 8 | -28.161997 | -49.627107 | Brazil |
| **F3482** | External and all internal | FLONA-SFP | sp 193 | *Pasipha* sp. 11 | -29.428944 | -50.392000 | Brazil |
| **F3796** | External, F and AC | PE Intervales | sp 193 | *Pasipha* sp. 11 | -24.269387 | -48.405467 | Brazil |
| **F3115** | External and all internal | PE Intervales | sp 194 | *Xerapoa* sp. 1 | -24.269387 | -48.405467 | Brazil |
| **F7438** | External and all internal | PN São Joaquim | sp 194 | *Xerapoa* sp. 1 | -27.960057 | -49.482761 | Brazil |
| **F3090** | External and AC | PE Intervales | sp 195 | *Xerapoa* sp. 2 | -24.269387 | -48.405467 | Brazil |
| **F3378** | External, F and AC | FLONA-SFP | sp 196 | *Xerapoa* sp. 4 | -29.43 | -50.37 | Brazil |
| **F3417** | External and all internal | FLONA-SFP | sp 197 | *Choeradoplana* sp. 1 | -29.436278 | -50.373694 | Brazil |
| **F3418** | External and F | FLONA-SFP | sp 198 | *Imbira* sp. 1 | -29.436278 | -50.373694 | Brazil |
| **F3251** | External | PE Tabuleiro | sp 198 | *Imbira* sp. 1 | -27.94 | -48.79 | Brazil |
| **F3388** | External, F and AC | FLONA-SFP | sp 199 | *Pasipha* sp. 2 | -29.427361 | -50.398389 | Brazil |
| **F3439** | External | FLONA-SFP | sp 200 | Rhynchodeminae sp. 2 | -29.42 | -50.39 | Brazil |
| **F3395** | External, A1, F and AC | FLONA-SFP | sp 201 | *Paraba* sp. 2 | -29.427361 | -50.398389 | Brazil |
| **F3468** | External, F and AC | FLONA-SFP | sp 202 | *Geoplana* sp. 1 | -29.42 | -50.39 | Brazil |
| **F7307** | External, A1 and AC | PN São Joaquim | sp 273 | *Geoplana* sp. 5 | -28.038422 | -49.613814 | Brazil |
| **F3383** | External and F | FLONA-SFP | sp 203 | *Geoplana* sp. 6 | -29.42 | -50.39 | Brazil |
| **F3472** | External, F and AC | FLONA-SFP | sp 204 | *Pasipha* sp. 6 | -29.42 | -50.39 | Brazil |
| **F3483** | External and AC | FLONA-SFP | sp 205 | *Pasipha* sp. 5 | -29.42 | -50.39 | Brazil |
| **F3394** | External, A1, F and AC | FLONA-SFP | sp 206 | *Geoplana* sp. 7 | -29.42 | -50.39 | Brazil |
| Lemos et al., 2014 | - | São Francisco de Paula/RS | sp 207 | *Choeradoplana minima* | -29.42 | -50.39 | Brazil |
| **F0406** | External and AC | FLONA-SFP | sp 208 | *Matuxia tymbyra* | -29.42 | -50.39 | Brazil |
| **F3425** | External, A1, F and AC | FLONA-SFP | sp 209 | *Paraba* sp. 1 | -29.42 | -50.39 | Brazil |
| **F7423** | External, A1 and AC | PN São Joaquim | sp 210 | *Barreirana* sp. 1 | -28.161702 | -49.628062 | Brazil |
| **F7273** | External and AC | PN São Joaquim | sp 211 | *Choeradoplana* sp. 3 | -28.282715 | -49.556282 | Brazil |
| **F7381** | External | PN São Joaquim | sp 211 | *Choeradoplana* sp. 3 | -28.236262 | -49.498931 | Brazil |
| **F7262** | External, F and AC | PN São Joaquim | sp 212 | *Choeradoplana* sp. 5 | -28.162037 | -49.627140 | Brazil |
| **F7292** | External, F and AC | PN São Joaquim | sp 213 | *Choeradoplana* sp. 9 | -28.038422 | -49.613814 | Brazil |
| **F6537** | External and AC | Alfredo Wagner/SC | sp 214 | *Choeradoplana* sp. 7 | -27.61727 | -49.35075 | Brazil |
| **F7419** | External and AC | PN São Joaquim | sp 215 | *Luteostriata* sp. 3 | -28.161771 | -49.627752 | Brazil |
| **F7413** | External and AC | PN São Joaquim | sp 216 | *Notogynaphallia* sp. 3 | -28.161876 | -49.627519 | Brazil |
| **F7241** | External, A1 and AC | PN São Joaquim | sp 217 | *Obama* sp. 9 | -28.140017 | -49.629717 | Brazil |
| **F7240** | External, A1 and AC | PN São Joaquim | sp 218 | *Obama* sp. 10 | -28.140421 | -49.627143 | Brazil |
| **F7318** | External, A1 and AC | PN São Joaquim | sp 219 | *Obama* sp. 11 | -28.038786 | -49.614039 | Brazil |
| **F7389** | External, A1 and AC | PN São Joaquim | sp 220 | *Obama* sp. 13 | -28.235706 | -49.498816 | Brazil |
| **F7378** | External, A1 and AC | PN São Joaquim | sp 221 | *Obama* sp. 16 | -28.235617 | -49.498897 | Brazil |
| **F7294** | External | PN São Joaquim | sp 222 | *Obama* sp. 17 | -28.038956 | -49.614243 | Brazil |
| **F7424** | External, A1, F and AC | PN São Joaquim | sp 223 | *Obama* sp. 20 | -28.161997 | -49.627107 | Brazil |
| **F7352** | External, A1 and AC | PN São Joaquim | sp 224 | *Obama* sp. 22 | -28.038432 | -49.613820 | Brazil |
| **F7362** | External, A1 and AC | PN São Joaquim | sp 225 | *Obama* sp. 21 | -28.038432 | -49.613820 | Brazil |
| **F7385** | External | PN São Joaquim | sp 226 | *Obama* sp. 7 | -28.235617 | -49.498897 | Brazil |
| **F3859** | External, A1, F and AC | PE Tabuleiro | sp 226 | *Obama* sp. 7 | -27.840735 | -48.924866 | Brazil |
| **F7373** | External | PN São Joaquim | sp 227 | *Paraba* sp. 4 | -28.162100 | -49.626466 | Brazil |
| **F7272** | External, A1, F and AC | PN São Joaquim | sp 228 | *Paraba* sp. 8 | -28.275805 | -49.549801 | Brazil |
| **F7315** | External, A1 and AC | PN São Joaquim | sp 229 | *Pasipha* sp. 1 | -28.038422 | -49.613814 | Brazil |
| **F7418** | External, A1, F and AC | PN São Joaquim | sp 230 | *Pasipha* sp. 3 | -28.161958 | -49.627265 | Brazil |
| **F7343** | External | PN São Joaquim | sp 231 | *Pasipha* sp. 4 | -28.040082 | -49.615061 | Brazil |
| **F7416** | External | PN São Joaquim | sp 232 | *Pasipha* sp. 9 | -28.161997 | -49.627107 | Brazil |
| **F7476** | External | PN São Joaquim | sp 233 | *Pasipha* sp. 12 | -27.960810 | -49.483000 | Brazil |
| **F7248** | External, A1, F and AC | PN São Joaquim | sp 234 | Geoplaninae 9 | -28.14187 | -49.63534 | Brazil |
| **F7268** | External and AC | PN São Joaquim | sp 235 | Geoplaninae 2 | -28.157942 | -49.638496 | Brazil |
| **F7486** | External, F and AC | PN São Joaquim | sp 236 | Geoplaninae 10 | -27.961773 | -49.483299 | Brazil |
| **F7363** | External, A1, F and AC | PN São Joaquim | sp 237 | Geoplaninae 12 | -28.038610 | -49.613914 | Brazil |
| **F7338** | External, A1 and AC | PN São Joaquim | sp 238 | Geoplaninae 13 | -28.039749 | -49.614906 | Brazil |
| **F7322** | External, F and AC | PN São Joaquim | sp 239 | *Xerapoa* sp. 3 | -28.039273 | -49.614560 | Brazil |
| **F7316** | External, F and AC | PN São Joaquim | sp 240 | *Xerapoa* sp. 5 | -28.040082 | -49.615061 | Brazil |
| **F3352** | External, A1, f and AC | PE Tabuleiro | sp 241 | Geoplaninae 1 | -27.86 | -48.91 | Brazil |
| **F3228** | External and all internal | PE Tabuleiro | sp 242 | *Obama* sp. 1 | -27.840735 | -48.924866 | Brazil |
| **F3238** | External and all internal | PE Tabuleiro | sp 243 | *Obama* sp. 2 | -27.84 | -48.92 | Brazil |
| **F3911** | External, A1, F and AC | PE Tabuleiro | sp 244 | *Choeradoplana* sp. 2 | -27.94 | -48.79 | Brazil |
| **F3186** | External and F | PE Tabuleiro | sp 245 | *Choeradoplana* sp. 4 | -27.94 | -48.79 | Brazil |
| **F3226** | External and all internal | PE Tabuleiro | sp 246 | *Choeradoplana tristriata* | -27.94 | -48.79 | Brazil |
| **F3340** | External, F and AC | PE Tabuleiro | sp 247 | *Obama* sp. 3 | -27.840735 | -48.924866 | Brazil |
| **F3918** | External and all internal | PE Tabuleiro | sp 248 | Geoplaninae 3 | -27.94 | -48.79 | Brazil |
| **F3823** | External and all internal | PE Tabuleiro | sp 249 | Geoplaninae 5 | -27.84 | -48.92 | Brazil |
| **F3241** | External, A1, F and AC | PE Tabuleiro | sp 250 | Geoplaninae 8 | -27.94 | -48.79 | Brazil |
| **F3904** | External, A1, F and AC | PE Tabuleiro | sp 251 | *Supramontana* sp. 1 | -27.84 | -48.92 | Brazil |
| **F3289** | External, A1, F and AC | PE Tabuleiro | sp 252 | *Luteostriata* sp. 2 | -27.840735 | -48.924866 | Brazil |
| **F3229** | External, A1 and AC | PE Tabuleiro | sp 253 | *Notogynaphallia* sp. 2 | -27.840735 | -48.924866 | Brazil |
| **F3888** | External, A1, F and AC | PE Tabuleiro | sp 254 | *Notogynaphallia* sp. 4 | -27.840735 | -48.924866 | Brazil |
| **F3280** | External, A1, F and AC | PE Tabuleiro | sp 255 | *Obama* sp. 6 | -27.840735 | -48.924866 | Brazil |
| **F6530** | External | Alfredo Wagner/SC | sp 255 | *Obama* sp. 6 | -27.61727 | -49.35075 | Brazil |
| **F3183** | External and AC | PE Tabuleiro | sp 256 | *Obama* sp. 19 | -27.94 | -48.79 | Brazil |
| **F3890** | External | PE Tabuleiro | Sp 257 | *Pasipha* sp. 10 | -27.94 | -48.79 | Brazil |
| **F6541** | External | Alfredo Wagner/SC | Sp 257 | *Pasipha* sp. 10 | -27.61727 | -49.35075 | Brazil |
| **F3185** | Externaland all internal | PE Tabuleiro | Sp 258 | Geoplaninae 11 | -27.94 | -48.79 | Brazil |
| **F3876** | External, A1 and AC | PE Tabuleiro | Sp 259 | Geoplaninae 15 | -27.840735 | -48.924866 | Brazil |
| **F4628** | Externaland AC | PNItajai | Sp 260 | *Cratera* sp. 4 | -27.05386 | -49.08606 | Brazil |
| **F1415** | External, F and AC | PN Itajaí | Sp 261 | Geoplaninae 4 | -27.05306 | -49.08528 | Brazil |
| **F4627** | Externaland AC | PNItajai | Sp 262 | Geoplaninae 14 | -27.05386 | -49.08606 | Brazil |
| **F1726** | External and all internal | Saint-Hilaire | Sp 263 | *Choeradoplana* sp. 6 | -25.469604 | -48.813739 | Brazil |
| **F1707** | External, A1 and F | Saint-Hilaire | Sp 264 | *Geoplana* sp. 3 | -25.469604 | -48.813739 | Brazil |
| **F3586** | External and all internal | Saint-Hilaire | Sp 265 | *Geoplana* sp. 8 | -25.469604 | -48.813739 | Brazil |
| **F1638** | External, A1, F and AC | Saint-Hilaire | Sp 266 | *Obama* sp. 12 | -25.71 | -48.62 | Brazil |
| **F1657** | External, A1, F and AC | Saint-Hilaire | Sp 267 | Geoplaninae 6 | -25.469604 | -48.813739 | Brazil |
| Carbayo et al (2013) | - | São Paulo/SP | Sp 268 | *Geoplana* sp. 2 | -23.453148 | -46.634488 | Brazil |
| CG Froehlich (1956a) | - | Ubatuba/SP | Sp 269 | *Cratera* sp. 6 | -23.423381 | -45.083399 | Brazil |
| Marcus (1951) | - | São Paulo/SP | Sp 270 | *Paraba* sp. 5 | -23.500212 | -46.702310 | Brazil |
