# Supplementary material for: Areas of endemism of land planarians (Platyhelminthes: Tricladida) in the Southern Atlantic Forest
Source: PLoS One. 2020 Jul 20;15(7):e0235949. doi: 10.1371/journal.pone.0235949 (PMC7371199; doi:10.1371/journal.pone.0235949)
Supplement: S2 Table — (DOCX) [file pone.0235949.s010.docx]

**Suppl. Tab. 2**. Records of land planarian species from literature excluded from this study and justification for such decision.

| **Species** | **Locality** | **Reference** | **Why excluded from analyses** |
| --- | --- | --- | --- |
| *Cephaloflexa bergi* | Teresópolis/RJ | Froehlich, 1956a | Missing information |
| *Cephaloflexa bergi* |  | Marcus, 1951 | There are numerous records elsewhere |
| *Cephaloflexa bergi* |  | Riester, 1938 | Missing information |
| *Choeradoplana iheringi* | São Leopoldo/RS | Froehlich, 1959 | See Carbayo et al 2017 |
| *Choeradoplana iheringi* |  | Amaral et al., 2014 | Misidentification |
| *Choeradoplana iheringi* |  | Froehlich, 1955 |  |
| *Choeradoplana iheringi* |  | Marcus, 1951 | See Carbayo et al., 2017 |
| *Choeradoplana iheringi* |  | Baptista et al., 2006 | See Carbayo et al., 2017 |
| *Choeradoplana iheringi* |  | Fick et al., 2006 | Complex case |
| *Choeradoplana iheringi* |  | Leal-Zanchet et al., 2011 | Complex case |
| *Choeradoplana iheringi* |  | Riester, 1938 | Outdated |
| *Choeradoplana langi* | Paraguay, Argentina | du Bois-Reymenond Marcus, 1953 | Doubtful record |
| *Geoplana brasiliensis* |  | Graff, 1899 | Belongs to genus Pseudogeoplana |
| *Geoplana distincta* |  | Graff, 1899 | Belongs to genus Pseudogeoplana |
| *Geoplana elegans* | Rio de Janeiro/RJ | Graff, 1899 | Identification dubious |
| *Geoplana ferussaci* |  | Graff, 1899 | Identification dubious |
| *Geoplana goeldii* |  | Graff, 1899 | Belongs to genus Pseudogeoplana |
| *Geoplana maximiliani* |  | Graff, 1899 | Belongs to genus Pseudogeoplana |
| *Geoplana nephelis* |  | Graff, 1899 | Belongs to genus Pseudogeoplana |
| *Geoplana perspicilata* |  | Graff, 1899 | Belongs to genus Pseudogeoplana |
| *Geoplana rostrata* |  | Graff, 1899 | Belongs to genus Pseudogeoplana |
| *Geoplana schultzei* |  | Graff, 1899 | Notogynaphallia schultzei |
| *Geoplana tristriata* |  | Graff, 1899 | Belongs to genus Pseudogeoplana |
| *Geoplana vaginuloides* |  | Graff, 1899 | Outdated |
| *Geoplana chita* |  | Froehlich, 1956b | See Almeida et al., 2018 |
| *Geoplana goetschi* |  | Froehlich, 1957 | Identification dubious |
| *Geoplana goetschi* | Teresópolis/RJ | Froehlich, 1956a | Identification dubious |
| *Geoplana goetschi* | Ubatuba/SP | Froehlich, 1956a | Identification dubious |
| *Geoplana leucophryna* |  | Marcus, 1951 | Synonym of Obama burmeisteri |
| *Geoplana marginata* | Blumenau/SC | Froehlich, 1959 | Identification dubious |
| *Geoplana marginata* | Curitiba, Caiobá/PR | Froehlich, 1956b | Identification dubious |
| *Geoplana marginata* |  | Froehlich, 1957 | Identification dubious |
| *Geoplana marginata* |  | Marcus, 1951 | Identification dubious |
| *Geoplana nigrofusca* |  | Graff, 1899 | Identification dubious |
| *Geoplana rufiventris* |  | Graff, 1899 | Identification dubious |
| *Geoplana vaginuloides* |  | Froehlich, 1957 | Misidentification |
| *Geoplana vaginuloides* |  | Marcus, 1951 | See Almeida et al., 2018 |
| *Geoplana vaginuloides* |  | Riester, 1938 | Misidentification |
| *Issoca rezendei* | Baia de Pranaguá/PR | Froehlich 1955 | Missing information |
| *Luteostriata muelleri* | Paranapiacaba/SP, Santo André/SP | Froehlich, 1959 | Doubts about conspecificity |
| *Notogynaphallia sexlineata (Riester, 1938)* |  | Riester, 1938 | Internal morphology unknown |
| *Obama burmeisteri* |  | Froehlich, 1955b | Locality not mentioned |
| *Obama carreirei* |  | Amaral et al, 2014 | Complex case |
| *Paraba gaucha* | “According to the collector, a common species at that locality, and also at Pôrto Alegre, capital of the State” | Froehlich, 1959 | Evidence lacking |
| *Pseudogeoplana elegans* | Blumenau/SC | Froehlich, 1959 | Dubious identification |
| *Pseudogeoplana maximiliani* | Blumenau/SC | Froehlich, 1959 | Dubious identification |
| *Pseudogeoplana nephelis* | Blumenau/SC | Froehlich, 1959 | Dubious identification |
| *Pseudogeoplana nephelis* | Blumenau/SC | Froehlich, 1959 | Dubious identification |
| *Pseudogeoplana octostriata* | Blumenau/SC | Froehlich, 1959 | Dubious identification |
| *Pseudogeoplana olivacea* | Blumenau/SC | Froehlich, 1959 | Dubious identification |
| *Pseudogeoplana pallida* | Blumenau/SC | Froehlich, 1959 | Dubious identification |
| *Pseudogeoplana rufiventris* | Blumenau/SC | Froehlich, 1959 | Dubious identification |
| *Pseudogeoplana tristriata* | Blumenau/SC | Froehlich, 1959 | Dubious identification |
