# Supplementary material for: Areas of endemism of land planarians (Platyhelminthes: Tricladida) in the Southern Atlantic Forest
Source: PLoS One. 2020 Jul 20;15(7):e0235949. doi: 10.1371/journal.pone.0235949 (PMC7371199; doi:10.1371/journal.pone.0235949)
Supplement: S3 Table — Abbreviations: CC, Congruence Core; WS, Widespread; MRE, Maximum Region of Endemism; SSC, South Santa Catarina; NSC, North Santa Catarina; Org: Serra dos Órgãos; PNSJ, Parque Nacional de São Joaquim; SFP, São Francisco de Paula; SMSP, Serra do Mar de São Paulo; SSP, Southern São Paulo; PR, Paraná; MIS, Misiones; POA, Porto Alegre; x, other cells. (DOCX) [file pone.0235949.s011.docx]

**Suppl. Tab. 3**. Geographic distribution of the land planarian species and their classification regarding their endemicity level. Abbreviations: CC, Congruence Core; WS, Widespread; MRE, Maximum Region of Endemism; SSC, South Santa Catarina; NSC, North Santa Catarina; Org: Serra dos Orgãos; PNSJ, Parque Nacional de São Joaquim; SFP, São Francisco de Paula; SMSP, Serra do Mar de São Paulo; SSP, Southern São Paulo; PR, Paraná; MIS, Misiones; POA, Porto Alegre; x, other cells.

| **Scientific name** | **AoE** | **Endemicity level** | **Single Record (SR)** |
| --- | --- | --- | --- |
| Geoplaninae 1 | SSC | CC | *+* |
| *Anisorhynchodemus pellucidus* (Graff, 1899) | NSC | CC | + |
| *Barreirana barreirana* (Riester, 1938) | Org/Ubatuba | WS |  |
| *Barreirana zebroides* (Riester, 1938) | Org | CC | + |
| *Barreirana* sp. 1 | PNSJ | CC | + |
| *Obama* sp. 1 | SSC | CC | + |
| *Paraba* sp. 10 | SSC | CC | + |
| *Cephaloflexa araucariana* Carbayo & Leal-Zanchet, 2003 | SFP | CC | + |
| *Cephaloflexa bergi* Graff, 1899 | Org, SMSP, NSC, SSC, SSP, PR, x | WS |  |
| *Cephaloflexa nataliae* (Froehlich, 1959) | NSC | CC | + |
| *Choeradoplana* sp. 1 | SFP | CC | + |
| *Choeradoplana abaiba* Carbayo et al., 2018 | SSC | CC |  |
| *Choeradoplana* sp. 2 | SSC | CC | + |
| *Choeradoplana agua* Carbayo et al., 2018 | SMMadalena | CC | + |
| *Choeradoplana albonigra* (Riester, 1938) | Org, SMMadalena | WS |  |
| *Choeradoplana banga* Carbayo & Froehlich, 2012 | SMSP | MRE |  |
| *Choeradoplana benyai* Lemos & Leal-Zanchet, 2014 | SSC, SFP | WS |  |
| *Choeradoplana bilix* Marcus, 1951 | x |  | + |
| *Choeradoplana bocaina* Carbayo & Froehlich, 2012 | Bocaina | CC | + |
| *Choeradoplana* sp. 3 | PNSJ | CC |  |
| *Choeradoplana catua* Froehlich, 1954 | Org | CC | + |
| *Choeradoplana* sp. 4 | SSC | CC | + |
| *Choeradoplana crassiphalla* Negrete & Brusa, 2012 | MIS | CC | + |
| *Choeradoplana ehrenreichi* Graff, 1899 | x |  | + |
| *Choeradoplana gladismariae* Carbayo & Froehlich, 2012 | SSP | CC | + |
| *Choeradoplana iheringi* Graff, 1899 | SFP, POA | WS |  |
| *Choeradoplana langi* (Dendy, 1894) | NSC | CC |  |
| *Choeradoplana* sp. 5 | PNSJ | CC | + |
| *Choeradoplana* sp. 6 | PR | CC | + |
| *Choeradoplana marthae* Froehlich, 1954 | SMSP | MRE |  |
| *Choeradoplana minima* Lemos & Leal-Zanchet, 2014 | SFP | CC | + |
| *Choeradoplana* sp. 7 | SSC | MRE-CC | + |
| *Choeradoplana pucupucu* Carbayo et al., 2018 | SMSP, Org | WS |  |
| *Choeradoplana* sp. 8 | SSP | CC | + |
| *Choeradoplana spatulata* Graff, 1899 | NSC | CC | + |
| *Choeradoplana* sp. 9 | PNSJ | CC | + |
| *Pseudogeoplana tristriata* (Schultze & Müller, 1857) | SSC | CC | + |
| *Cratera anamariae* Carbayo, 2015 | Org | CC | + |
| *Cratera arucuia* Lago-Barcia & Carbayo, 2018 | SSP | CC | + |
| *Cratera aureomaculata* Rossi & Leal-Zanchet, 2017 | Três Barras | CC | + |
| *Cratera crioula* (Froehlich, 1955) | SMSP | CC | + |
| *Cratera cryptolineata* Rossi & Leal-Zanchet, 2017 | Três Barras | CC | + |
| *Cratera cuarassu* Carbayo & Almeida, 2015 | SMMadalena | CC | + |
| *Cratera hina* (Marcus, 1951) | SMSP | CC | + |
| *Cratera joia* (Froehlich, 1956) | PR | CC | + |
| *Cratera* sp. 1 | SSP | CC | + |
| *Cratera* sp. 2 | PR | CC | + |
| *Cratera* sp. 3 | PR | CC | + |
| *Cratera* sp. 4 | NSC | CC | + |
| *Cratera* sp. 5 | PNSJ | CC | + |
| *Cratera nigrimarginata* Rossi & Leal-Zanchet, 2017 | x |  | + |
| *Cratera* sp. 6 | Ubatuba | CC | + |
| *Cratera ochra* Rossi et al., 2016 | SFP, PNSJ | MRE |  |
| *Cratera picuia* Lago-Barcia & Carbayo, 2018 | Matinhos | CC | + |
| *Cratera pseudovaginuloides* (Riester, 1938) | Org | CC | + |
| *Cratera steffeni* Rossi et al., 2014 | SFP | CC | + |
| *Cratera tamoia* (Froehlich, 1955) | Org | CC |  |
| *Cratera taxiarcha* (Marcus, 1951) | SMSP | CC | + |
| *Cratera viridimaculata* Negrete & Brusa, 2016 | MIS | CC | + |
| *Cratera yara* (Froehlich, 1955) | Org | CC | + |
| Geoplaninae 2 | PNSJ | CC | + |
| *Obama* sp. 3 | SSC | CC | + |
| *Geobia subterranea* (Schultze & Müller, 1857) | ORG, SMSP, SCN, POA, PR, x | WS |  |
| Geoplaninae 16 | SFP | CC | + |
| *Geoplana apua* Almeida & Carbayo, 2018 | Org | MRE? | + |
| *Imbira* sp. 2 | SMSP | CC | + |
| *Geoplana boraceia* Almeida & Carbayo, 2018 | x |  | + |
| *Geoplana cambara* Almeida & Carbayo, 2018 | Matinhos | CC |  |
| *Geoplana cananeia* Almeida & Carbayo, 2018 | x |  | + |
| *Geoplana caraguatatuba* Almeida & Carbayo, 2018 | x |  | + |
| *Geoplana carrierei* sensu Marcus, 1951 | SMSP, POA | WS |  |
| *Geoplana chita* Froehlich, 1956 | PR | CC | + |
| *Geoplana* sp. 1 | PR | CC | + |
| *Geoplana chiuna* Froehlich, 1955 | x |  | + |
| *Geoplana duca* Marcus, 1951 | SMSP | CC | + |
| *Geoplana ferussaci* sensu Riester, 1938 | ORG, SMSP, x | WS |  |
| *Geoplana fragai* Froehlich, 1955 | Org | CC | + |
| *Geoplana goetschi* Riester, 1938 | Org | CC | + |
| *Geoplana ibiuna* Almeida & Carbayo, 2018 | x | MRE |  |
| *Paraba incognita* (Riester, 1938) | Org | CC | + |
| *Geoplana iporanga* Almeida & Carbayo, 2018 | SSP | CC | + |
| *Geoplana jandira* Froehlich, 1955 | Org | CC | + |
| *Geoplana mogi* Almeida & Carbayo, 2018 | SMSP | CC |  |
| Geoplaninae 17 | PNSJ | CC | + |
| *Geoplana nigra* Froehlich, 1959 | POA | CC | + |
| *Geoplana notophthalma* Riester, 1938 | Org | MRE |  |
| Geoplaninae 18 | SFP | CC | + |
| *Geoplana paranapiacaba* Almeida & Carbayo, 2018 | SMSP | CC | + |
| *Geoplana pavani* Marcus, 1951 | SMSP | CC | + |
| *Geoplana picta* Froehlich, 1956 | Ubatuba |  | + |
| *Geoplana piratininga* Almeida & Carbayo, 2018 | SMSP | CC | + |
| Geoplaninae 19 | SFP | CC | + |
| *Geoplana pulchella* (Schultze & Müller, 1857) | NSC, SSC | WS |  |
| *Geoplana quagga* Marcus, 1951 | ORG, SMSP, NSC | WS |  |
| *Geoplana regia* Froehlich, 1954 | x |  | + |
| *Geoplana* sp. 2 | PR | CC | + |
| *Geoplana toriba* Froehlich, 1957 | SSP | MRE | + |
| *Geoplana* sp. 3 | SSP | CC | + |
| *Geoplana vaginuloides* (Darwin, 1844) | Org | CC | + |
| Geoplaninae 3 | SSC | CC | + |
| Geoplaninae 4 | NSC | CC | + |
| Geoplaninae 5 | SSC | CC | + |
| Geoplaninae 6 | PR | CC | + |
| Geoplaninae 7 | SSP | CC | + |
| Geoplaninae 8 | SSC | CC | + |
| *Imbira flavonigra* Amaral & Leal-Zanchet, 2018 | SFP | CC | + |
| *Imbira* sp. 1 | SFP, SSC | WS |  |
| *Imbira guaiana* (Leal-Zanchet & Carbayo, 2001) | SFP, POA | WS |  |
| *Imbira marcusi* Carbayo et al., 2013 | SMSP, SSP, PR, x | WS |  |
| *Imbira negrita* Negrete & Brusa, 2017 | MIS | MRE | + |
| *Issoca assanga* Araujo & Carbayo, 2018 | SMMadalena | MRE |  |
| *Issoca jandaia* (Froehlich, 1954) | SMSP, SSP | WS |  |
| *Issoca piranga* (Froehlich, 1954) | Org | CC | + |
| *Issoca potyra* (Froehlich, 1957) | SSP | MRE |  |
| *Issoca rezendei* (Schirch, 1929) | ORG, SMSP, SCN, PR, x | WS |  |
| *Issoca* sp. 2 | SSP | CC | + |
| *Luteostriata abundans* (Graff, 1899) | POA | CC |  |
| *Luteostriata arturi* (Lemos & Leal-Zanchet, 2008) | SFP | CC |  |
| *Luteostriata caissara* (Froehlich, 1954) | SMSP, Org | WS |  |
| *Luteostriata ceciliae* (Froehlich & Leal-Zanchet, 2003) | SFP | CC |  |
| *Luteostriata* sp. 1 | SSP | CC | + |
| *Luteostriata ernesti* (Leal-Zanchet & Froehlich, 2006) | SMSP, SCS, POA, x | WS |  |
| *Luteostriata* sp. 2 | SSC | CC | + |
| *Luteostriata fita* (Froehlich, 1959) | NSC | CC | + |
| *Luteostriata* sp. 3 | PNSJ | CC | + |
| *Luteostriata graffi* (Leal-Zanchet & Froehlich, 2006) | SFP, POA, Flori | WS |  |
| *Luteostriata* sp. 4 | Matinhos | CC | + |
| *Luteostriata muelleri* (Diesing, 1861) | NSC, SSC | WS |  |
| *Luteostriata pseudoceciliae* (Lemos & Leal-Zanchet, 2008) | SFP | CC | + |
| *Matuxia tymbyra* Rossi & Leal-Zanchet, 2019 | SFP | CC | + |
| *Matuxia matuta* (Froehlich, 1954) | Org, SMMadalena | WS |  |
| *Matuxia tuxaua* (Froehlich, 1954) | SMSP, Org | WS |  |
| *Notogynaphallia atra* (Schultze & Müller, 1857) | SFP, NSC | WS |  |
| *Notogynaphallia mourei* (Froehlich, 1956) | PR | MRE |  |
| *Notogynaphallia* sp. 1 | NSC, POA | WS |  |
| *Notogynaphallia parca* (Froehlich, 1954) | SMSP | CC | + |
| *Notogynaphallia* sp. 2 | SSC | CC | + |
| *Notogynaphallia plumbea* (Froehlich, 1956) | SMSP, SCS, SSP, PR, x | WS |  |
| *Notogynaphallia* sp. 3 | PNSJ | CC | + |
| *Notogynaphallia sexstriata* (Graff, 1899) | ORG, SMSP, SCN, POA, PR, x | WS |  |
| *Notogynaphallia* sp. 4 | SSC | CC | + |
| *Obama* sp. 5 | PR | CC | + |
| *Obama anthropophila* Amaral et al., 2015 | SCN, SCS, SSP, SFP, POA, x | WS |  |
| *Obama apeva* (Froehlich, 1959) | NSC, SSC | WS |  |
| *Obama applanata* (Graff, 1899) | Org | CC | + |
| *Obama argus* (Graff, 1899) | Org | MRE | + |
| *Obama assu* (Froehlich, 1959) | NSC | MRE-CC |  |
| *Obama baptistae* (Leal-Zanchet & Oliveira, 2012) | Smaria | CC | + |
| *Obama braunsi* (Graff, 1899) | SMSP | MRE |  |
| *Obama burmeisteri* (Schultze & Müller, 1857) | ORG, SMSP, SCN, POA, x | WS |  |
| *Obama carbayoi* (Oliveira & Leal-Zanchet, 2012) | Derrubadas | CC | + |
| *Obama carinata* (Riester, 1938) | SMSP, SSP | WS |  |
| *Obama catharina* (Hyman, 1957) | x |  | + |
| *Obama* sp. 6 | SSC | MRE-CC |  |
| *Obama* sp. 7 | SSC | MRE |  |
| *Obama decidualis* Amaral & Leal-Zanchet, 2015 | Smaria | CC | + |
| *Obama dictyonota* (Riester, 1938) | Org | CC | + |
| *Obama divae* (Marcus, 1951) | SMSP, SSP | WS |  |
| *Obama eudoxiae* (Ogren & Kawakatsu, 1990) | Org | CC | + |
| *Obama eudoximariae* (Ogren & Kawakatsu, 1990) | Org | CC | + |
| *Obama evelinae* (Marcus, 1951) | SMSP | MRE |  |
| *Obama ferussaci* sensu Graff, 1899 | PR, SSP | WS |  |
| *Obama* sp. 9 | PNSJ | CC | + |
| *Obama ficki* (Amaral & Leal-Zanchet, 2012) | SFP, POA | WS |  |
| *Obama fryi* (Graff, 1899) | Org | CC |  |
| *Obama glieschi* (Froehlich, 1959) | Derrubadas | MRE | + |
| *Obama itatiayana* (Schirch, 1929) | x |  | + |
| *Obama josefi* (Carbayo & Leal-Zanchet, 2001) | SFP | CC |  |
| *Obama ladislavii* (Graff, 1899) | SCN, SCS, POA, SFP | WS |  |
| *Obama livia* (Froehlich, 1954) | SMSP | CC |  |
| *Obama maculipunctata* (Rossi et al., 2016) | SFP | CC |  |
| *Obama* sp. 10 | PNSJ | CC | + |
| *Obama* sp. 11 | PNSJ | CC | + |
| *Obama marmorata* (Schultze & Müller, 1857) | NSC, SSC | WS |  |
| *Obama metzi* (Graff, 1899) | SMSP | CC |  |
| *Obama* sp. 12 | Matinhos | CC | + |
| *Obama nungara* Carbayo et al., 2016 | SSC | MRE-CC |  |
| *Obama* sp. 13 | PNSJ | CC | + |
| *Obama otavioi* Carbayo, 2016 | SMSP | CC | + |
| *Obama* sp. 14 | PNSJ | CC | + |
| *Obama poca* (Froehlich, 1957) | SMSP | MRE | + |
| *Obama polyophthalma* (Graff, 1899) | SCN, POA, PR | WS |  |
| *Obama* sp. 16 | PSNJ | CC | + |
| *Obama* sp. 17 | SSP | CC | + |
| *Obama riesteri* (Froehlich, 1954) | Org | CC | + |
| *Obama* sp. 18 | PSNJ | CC | + |
| *Obama* sp. 19 | SSC | CC | + |
| *Obama schubarti* (Froehlich, 1957) | SMSP | MRE |  |
| *Obama* sp. 20 | PSNJ | CC | + |
| *Obama* sp. 21 | PSNJ | CC | + |
| *Obama* sp. 2 | PSNJ | CC | + |
| *Obama trigueira* (Froehlich, 1955) | Org | CC |  |
| *Obama* sp. 8 | SSP | CC | + |
| *Paraba* sp. 1 | SFP | CC | + |
| *Paraba caapora* (Froehlich, 1958) | NSC, SSP | WS |  |
| *Paraba cassula* (Froehlich, 1955) | Org | CC | + |
| *Paraba franciscana* ( Leal-Zanchet & Carbayo, 2001) | SFP | CC |  |
| *Paraba gaucha* (Froehlich, 1959) | POA | CC |  |
| *Paraba goettei* (Schirch, 1929) | Org | CC | + |
| *Paraba* sp. 2 | SFP | CC | + |
| *Paraba* sp. 3 | SSP | CC | + |
| *Paraba* sp. 4 | PNSJ | CC | + |
| *Paraba multicolor* (Graff, 1899) | ORG, SMSP, SCS, POA, SSP, x | WS |  |
| *Paraba* sp. 5 | SMSP | CC | + |
| *Paraba phocaica* (Marcus, 1951) | SMSP | CC | + |
| *Paraba piriana* (Almeida & Carbayo, 2012) | Org | CC | + |
| *Paraba preta* (Riester, 1938) | Org | CC | + |
| *Paraba* sp. 6 | SSP | CC | + |
| *Paraba rubidolineata* (Baptista & Leal-Zanchet, 2005) | SFP, SSC | WS |  |
| *Paraba* sp. 7 | Bocaina |  | + |
| *Paraba suva* (Froehlich, 1959) | NSC | CC | + |
| *Paraba tapira* (Froehlich, 1958) | SSP | MRE |  |
| *Paraba* sp. 8 | PNSJ | CC | + |
| *Paraba tingauna* (Kishimoto & Carbayo, 2012) | SSC | CC |  |
| *Paraba* sp. 9 | SSP | CC | + |
| *Pasipha* sp. 1 | PNSJ | CC | + |
| *Pasipha* sp. 2 | SFP | CC | + |
| *Pasipha astraea* (Marcus, 1951) | SMSP | CC | + |
| *Pasipha atla* Negrete & Brusa. 2016 | MIS | CC | + |
| *Pasipha backesi* Leal-Zanchet et al., 2012 | SFP | CC | + |
| *Pasipha biseminalis* (Riester, 1938) | Org | CC | + |
| *Pasipha* sp. 3 | PNSJ | CC | + |
| *Pasipha brevilineata* Leal-Zanchet et al., 2012 | SFP | CC | + |
| *Pasipha* sp. 4 | PNSJ | CC | + |
| *Pasipha caeruleonigra* (Riester, 1938) | Org | CC | + |
| *Pasipha cafusa* (Froehlich, 1956) | Ubatuba | CC | + |
| *Pasipha chimbeva* (Froehlich, 1954) | SMSP | CC | + |
| *Pasipha* sp. 5 | SFP | CC | + |
| *Pasipha hauseri* (Froehlich, 1959) | POA | CC |  |
| *Pasipha johnsoni* Negrete & Brusa, 2016 | MIS | CC | + |
| *Pasipha* sp. 6 | SFP | CC | + |
| *Pasipha mbya* Negrete & Brusa, 2016 | MIS | CC |  |
| *Pasipha mesoxantha* Amaral & Leal-Zanchet, 2016 | Smaria | CC | + |
| *Pasipha* sp. 7 | PR | CC | + |
| *Pasipha oliverioi* (Froehlich, 1959) | Org | CC | + |
| *Pasipha pasipha* (Marcus, 1951) | SMSP | CC | + |
| *Pasipha* sp. 8 | SSC, SSP | WS |  |
| *Pasipha* sp. 9 | NSC, PNSJ | MRE |  |
| *Pasipha pinima* (Froehlich, 1954) | ORG, SMSP, SCS, SSP, x | WS |  |
| *Pasipha* sp. 10 | SSC | MRE-CC |  |
| *Pasipha plana* (Schirch, 1929) | Org | CC | + |
| *Pasipha* sp. 11 | SFP, SSP | WS |  |
| *Pasipha quirogai* Negrete & Brusa, 2017 | MIS | MRE | + |
| *Pasipha rosea* (Froehlich, 1954) | SMSP, PR | WS |  |
| *Pasipha* sp. 12 | Bocaina | CC | + |
| *Pasipha splendida* (Graff, 1899) | Org | CC | + |
| *Pasipha* sp. 13 | PNSJ | CC | + |
| *Pasipha tapetilla* (Marcus, 1951) | NSC, SSC | WS |  |
| *Pasipha turvensis* Amaral & Leal-Zanchet, 2016 | Derrubadas | CC | + |
| *Pasipha velina* (Froehlich, 1959) | NSC | CC |  |
| *Pasipha velutina* (Riester, 1938) | x |  | + |
| Geoplaninae 9 | PNSJ | CC | + |
| Geoplaninae 10 | PNSJ | CC | + |
| Geoplaninae 11 | SSC | CC | + |
| Geoplaninae 12 | PNSJ | CC | + |
| Geoplaninae 13 | PNSJ | CC | + |
| Rhynchodeminae 1 | NSC | CC | + |
| Rhynchodeminae 2 | SFP | CC | + |
| *Rhynchodemus hectori* Graff, 1897 | Org | CC | + |
| *Rhynchodemus scius* Du Bois-Reymond Marcus, 1955 | Ubatuba | CC | + |
| *Supramontana argentina* Negrete et al., 2014 | MIS | CC |  |
| Geoplaninae 20 | SSC | CC | + |
| *Supramontana irritata* Carbayo & Leal-Zanchet, 2003 | SFP | CC | + |
| Geoplaninae 14 | NSC | CC | + |
| Geoplaninae 15 | SSC | CC | + |
| *Xerapoa hystrix* Froehlich, 1954 | PR, SMSP | WS |  |
| *Xerapoa* sp. 1 | SSP, SSC | WS |  |
| *Xerapoa* sp. 2 | SSP | CC | + |
| *Xerapoa pseudorhynchodemus* (Riester, 1938) | ORG, SMSP, SCN, SCS, PR, x | WS |  |
| *Xerapoa* sp. 3 | PNSJ | CC | + |
| *Xerapoa* sp. 4 | SFP | CC | + |
| *Xerapoa trina* (Marcus, 1951) | SMSP, Org | WS |  |
| *Xerapoa una* Froehlich, 1954 | NSC | CC | + |
| *Xerapoa* sp. 5 | PNSJ | CC | + |
