# Supplementary material for: Areas of endemism of land planarians (Platyhelminthes: Tricladida) in the Southern Atlantic Forest
Source: PLoS One. 2020 Jul 20;15(7):e0235949. doi: 10.1371/journal.pone.0235949 (PMC7371199; doi:10.1371/journal.pone.0235949)
Supplement: S1 Material — (DOCX) [file pone.0235949.s012.docx]

Amaro, RC et al. 2012. Mol. Phyl. Evol. 62.

Amorim, DS. 2009. In: Pape, R et al. eds. Leiden: Brill, 71-97.

Amorim, DS et al. 2009. Syst. Entom. 34.

Amorim, DS, Pires, MRS. 1996. In: Bicudo, CEM, Menezes, NA, eds. São Paulo: CNPq, 183-219.

Batalha-Filho, H et al. 2010. Apidol 41.

Bragagnolo, C et al. 2015. Invert. Syst. 29.

Brunes, TO et al. 2010. Mol. Phyl. Evol. 57.

Cabanne, GS et al. 2014. Biol. J. Linn. Soc. 113.

Carnaval, AC et al. 2009. Science 323.

Costa, L. P. et al. 2000. Biotropica 32.

D’Horta, FM et al. 2011. Mol. Ecol. 20.

DaSilva, MB et al. 2015a. Cladistics 31.

DaSilva, MB et al. 2015b. In: Carvalho, CJB, Almeida, EAB, eds. 2nd edn. São Paulo: Editora Roca, 227-244.

Grazziotin, FG et al. 2006. Mol. Ecol. 15.

Martins, FM et al. 2009. BMC Evol. Bio. 9.

Müller, P. 1973. Biogeographica 2.

Pellegrino, KCM et al. 2005. Biol. J. Linn. Soc. 85.

Prance, GT. 1982. Columbia University Press, New York.

Resende, H.C. et al. 2010. Biol. J. Linn. Soc. 101.

Sigrist, MS, Carvalho, CJB 2009. Biota Neotr. 9.

Silva, JMC et al. 2004. Glob. Eco. Biogeogr. 13.

Silva, SM, Moraes-Barros, N et al. 2012. Biol. J. Linn. Soc. 107.

Thomé, MTC et al. 2010. Mol. Phyl. Evol. 55.

Valdez, L, D’Elía, G. 2013. J. Mammal. 94.
