# Supplementary material for: Areas of endemism of land planarians (Platyhelminthes: Tricladida) in the Southern Atlantic Forest
Source: PLoS One. 2020 Jul 20;15(7):e0235949. doi: 10.1371/journal.pone.0235949 (PMC7371199; doi:10.1371/journal.pone.0235949)
Supplement: Attachment — Submitted filename: PONE-D-19-32242_reviewer.pdf [file pone.0235949.s014.pdf]

PLOS ONE

Numerous areas of endemism of land planarians (Platyhelminthes: Tricladida) in the Southern Atlantic Forest as inferred from combined methods

--Manuscript Draft--

**Manuscript Number:**	PONE-D-19-32242
**Article Type:**	Research Article
**Full Title:**	Numerous areas of endemism of land planarians (Platyhelminthes: Tricladida) in the Southern Atlantic Forest as inferred from combined methods
**Short Title:**	Areas of endemism of land planarians (Platyhelminthes: Tricladida) in the Southern Atlantic Forest.
**Corresponding Author:**	Domingo Lago-Barcia<br>Universidade de Sao Paulo Instituto de Biociencias<br>São Paulo, SP BRAZIL
**Keywords:**	Geoplaninae; Terrestrial flatworms; Brazil; rain forest; NDM; EA; GIE; biogeography
**Abstract:**	Areas of endemism are the main study units in analytical biogeographic methods, and are often defined as an area with two or more endemic species living in them, presenting substantial congruence among their range limits. We explored the distribution of land planarians (Geoplanidae, Platyhelminthes) across the southern region of the Brazilian Atlantic forest (from the State of Rio de Janeiro, to the State of Rio Grande do Sul) utilizing DaSilva's et al. (2015) protocol. We used three methods, Endemicity Analysis (EA), Geographical Interpolation of Endemism (GIE), and a manual search. We identified nine AoEs of terrestrial flatworms in the Southern Atlantic forest through EA and GIE. Alongside, the manual search identified eight additional AoEs. Performance of the methodologies is discussed. AoEs of land planarians can be explained through vicariance events combined with their physiological and ecological own limitations. These AoEs are congruent with fine-scale approaches such as that of harvestmen. Most land planarians have revealed to present a very small distributional range evidencing their potential as a good model for fine-scale studies of AoEs.
**Order of Authors:**	Domingo Lago-Barcia
	Mauricio Bernardino DaSilva
	Luis Americo Conti
	Fernando Carbayo
**Additional Information:**	
**Question**	**Response**
**Financial Disclosure**<br><br>Enter a financial disclosure statement that describes the sources of funding for the work included in this submission. Review the submission guidelines for detailed	The present work was financed with support from Fundação de Amparo à Pesquisa do Estado de São Paulo (FAPESP) (Proc. 2016/18295-5) (http://www.fapesp.br/) and a Graduate Fellowship from Conselho Nacional de Desenvolvimento Científico e Tecnológico (CNPq) (http://www.cnpq.br/). The funders had no role in study design, data collection and analysis, decision to publish, or

Powered by Editorial Manager® and ProduXion Manager® from Aries Systems Corporation

requirements. View published research articles from *PLOS ONE* for specific examples.<br><br>This statement is required for submission and **will appear in the published article** if the submission is accepted. Please make sure it is accurate.<br><br>**Unfunded studies**<br>Enter: *The author(s) received no specific funding for this work.*<br><br>**Funded studies**<br>Enter a statement with the following details:<br>• Initials of the authors who received each award<br>• Grant numbers awarded to each author<br>• The full name of each funder<br>• URL of each funder website<br>• Did the sponsors or funders play any role in the study design, data collection and analysis, decision to publish, or preparation of the manuscript?<br>• **NO** - Include this sentence at the end of your statement: *The funders had no role in study design, data collection and analysis, decision to publish, or preparation of the manuscript.*<br>• **YES** - Specify the role(s) played.<br><br>* typeset	preparation of the manuscript.
**Competing Interests**<br><br>Use the instructions below to enter a competing interest statement for this submission. On behalf of all authors, disclose any competing interests that could be perceived to bias this work—acknowledging all financial support and any other relevant financial or non-financial competing interests.<br><br>This statement **will appear in the published article** if the submission is accepted. Please make sure it is accurate. View published research articles from *PLOS ONE* for specific examples.	The authors have declared that no competing interests exist.

**NO authors have competing interests**<br><br>Enter: *The authors have declared that no competing interests exist.*<br><br>**Authors with competing interests**<br><br>Enter competing interest details beginning with this statement:<br><br>*I have read the journal's policy and the authors of this manuscript have the following competing interests: [insert competing interests here]*<br><br>* typeset	
**Ethics Statement**<br><br>Enter an ethics statement for this submission. This statement is required if the study involved:<br><br>• Human participants<br>• Human specimens or tissue<br>• Vertebrate animals or cephalopods<br>• Vertebrate embryos or tissues<br>• Field research<br><br>Write "N/A" if the submission does not require an ethics statement.<br><br>General guidance is provided below. Consult the submission guidelines for detailed instructions. **Make sure that all information entered here is included in the Methods section of the manuscript.**	ICMBio 57798-1, ICMBio 11748-4 (Instituto Chico Mendes de Conservação da Biodiversidade (ICMBio)); EBBAut.020/2013 (Museu de Zoologia da Universidade de São Paulo (MZUSP)); 42.520/2007 (Instituto do Meio Ambiente de Santa Catarina (IMA) (former FATMA)).

Powered by Editorial Manager® and ProduXion Manager® from Aries Systems Corporation

**Format for specific study types**<br><br>**Human Subject Research (involving human participants and/or tissue)**<br>• Give the name of the institutional review board or ethics committee that approved the study<br>• Include the approval number and/or a statement indicating approval of this research<br>• Indicate the form of consent obtained (written/oral) or the reason that consent was not obtained (e.g. the data were analyzed anonymously)<br><br>**Animal Research (involving vertebrate animals, embryos or tissues)**<br>• Provide the name of the Institutional Animal Care and Use Committee (IACUC) or other relevant ethics board that reviewed the study protocol, and indicate whether they approved this research or granted a formal waiver of ethical approval<br>• Include an approval number if one was obtained<br>• If the study involved *non-human primates*, add *additional details* about animal welfare and steps taken to ameliorate suffering<br>• If anesthesia, euthanasia, or any kind of animal sacrifice is part of the study, include briefly which substances and/or methods were applied<br><br>**Field Research**<br><br>Include the following details if this study involves the collection of plant, animal, or other materials from a natural setting:<br>• Field permit number<br>• Name of the institution or relevant body that granted permission	
**Data Availability**<br><br>Authors are required to make all data underlying the findings described fully available, without restriction, and from the time of publication. PLOS allows rare exceptions to address legal and ethical concerns. See the PLOS Data Policy and FAQ for detailed information.	Yes - all data are fully available without restriction

A Data Availability Statement describing where the data can be found is required at submission. Your answers to this question constitute the Data Availability Statement and **will be published in the article**, if accepted.<br><br>**Important:** Stating 'data available on request from the author' is not sufficient. If your data are only available upon request, select 'No' for the first question and explain your exceptional situation in the text box.<br><br>Do the authors confirm that all data underlying the findings described in their manuscript are fully available without restriction?	
**Describe where the data may be found in full sentences. If you are copying our sample text, replace any instances of XXX with the appropriate details.**<br><br>• If the data are **held or will be held in a public repository**, include URLs, accession numbers or DOIs. If this information will only be available after acceptance, indicate this by ticking the box below. For example: *All XXX files are available from the XXX database (accession number(s) XXX, XXX.).*<br>• If the data are all contained **within the manuscript and/or Supporting Information files**, enter the following: *All relevant data are within the manuscript and its Supporting Information files.*<br>• If neither of these applies but you are able to provide **details of access elsewhere**, with or without limitations, please do so. For example:<br><br>*Data cannot be shared publicly because of [XXX]. Data are available from the XXX Institutional Data Access / Ethics Committee (contact via XXX) for researchers who meet the criteria for access to confidential data.*<br><br>*The data underlying the results presented in the study are available from (include the name of the third party*	All relevant data are within the manuscript and its Supporting Information files.

and contact information or URL).<br>• This text is appropriate if the data are owned by a third party and authors do not have permission to share the data.<br><br>* typeset	
Additional data availability information:	

Powered by Editorial Manager® and ProduXion Manager® from Aries Systems Corporation

Manuscript

Click here to access/download;Manuscript;Lago-Barcia et al_ENDEMISM_PlosONE.docx

Numerous areas of endemism of land planarians (Platyhelminthes: Tricladida) in the Southern Atlantic Forest as inferred from combined methods

Domingo Lago-Barcia[1, 2] *, Mauricio Bernardino DaSilva[3], Luis Americo Conti[4] and Fernando Carbayo[1, 2]

1. Laboratório de Ecologia e Evolução, Escola de Artes, Ciências e Humanidades (EACH), Universidade de São Paulo (USP), Av. Arlindo Bettio, 1000, São Paulo, SP, 03828-000, Brazil.

2. Departamento de Zoologia, Instituto de Biociências, Universidade de São Paulo (USP), Rua do Matão, Tv. 14, 321, São Paulo, SP, 05508-090, Brazil.

3. Departamento de Sistemática e Ecologia, CCEN, Universidade Federal da Paraíba, Cidade Universitária, Conj. Pres. Castelo Branco III, João Pessoa, PB, 58033-455, Brazil.

4. Escola de Artes, Ciências e Humanidades (EACH), Universidade de São Paulo (USP), Av. Arlindo Bettio, 1000, São Paulo, SP, 03828-000, Brazil.

** Corresponding Author:*

E-mail: domingo.lagobarcia@gmail.com

Abstract

Areas of endemism are the main study units in analytical biogeographic methods, and are often defined as an area with two or more endemic species living in them, presenting substantial congruence among their range limits. We explored the distribution of land planarians (Geoplanidae, Platyhelminthes) across the southern region of the Brazilian Atlantic forest (from the State of Rio de Janeiro, to the State of Rio Grande do Sul) utilizing DaSilva's *et al.* (2015) protocol. We used three methods, Endemicity Analysis (EA), Geographical Interpolation of Endemism (GIE), and a manual search. We identified nine AoEs of terrestrial flatworms in the Southern Atlantic forest through EA and GIE. Alongside, the manual search identified eight additional AoEs. Performance of the methodologies is discussed. AoEs of land planarians can be explained through vicariance events combined with their physiological and ecological own limitations. These AoEs are congruent with fine-scale approaches such as that of harvestmen. Most land planarians have revealed to present a very small distributional range evidencing their potential as a good model for fine-scale studies of AoEs.

Keywords: Geoplaninae, terrestrial flatworms, Brazil, rain forest, NDM, EA, GIE, biogeography.

Introduction

Historical biogeography has evolved substantially since its beginning three centuries ago [1]. One of its main objectives is to detect areas of endemism (AoEs) [2]. Because AoEs are the main study units in analytical biogeographic methods [3, 4], they can serve to design biogeographic regionalization schemes [5], to infer historical relationships between them [6] to study organism-climate dynamics [7], and as a criterion to identify areas for conservation [8, 9, 10, 11]. Different methodologies have been proposed to achieve said objectives [12-21] with different animal taxons [3, 22-26].

AoEs are often defined as an area with two or more endemic species living in them, presenting substantial congruence among their range limits [27]. Those basic criteria are based on the concept of a common biogeographical origin and/or isolation of those species ranges [3, 28].

The Atlantic Forest (AF) is one of the largest forests in the Americas, extending over approximately 150 million hectares [29]. Longitudinal, latitudinal and altitudinal ranges have modeled different climatic and environmental conditions producing distinct environs in it [30, 31]. These different conditions have created a complex biome that is characterized by high levels of endemism (averaging nearly 50% overall, and as high as 95% in some groups; [32]).

Since the proposition of the first biogeographic regionalization of the world, by Wallace [33], many studies identifying AoEs have been carried out, including those in the AF in the 20th century. Through the years, the AoEs proposed for the AF vary in size and number depending on the taxonomic group being studied. Müller [22] divided the AF in 3 AoEs using amphibians, reptiles, birds and mammals. He named them Pernambuco, Bahia and Paulista. The author considered AoEs as those areas where endemic species distributions overlapped. Kinzey [23] identified the same AoEs as Müller, though smaller in size, using primate distributions. Cracraft [3] suggested two AoEs using bird distributions; one of these areas extends from Pernambuco to Santa Catarina, and the other covers the so called “Paranaense Forest”, as named by Morrone [28]. Amorim & Pires [34] detected two big AoEs, using diptera and primate distributions. These two big areas are located in the borderline between Espírito Santo and Rio de Janeiro states and were subdivided into smaller areas, namely the northern area, in turn with four subareas, and the southern area, with two. Costa *et al.*, [35] also discovered two big AoEs using mammals distribution. Differently from Amorim & Pires [34] and Müller [22], they positioned the limit between these two areas 300 km further south. Though concordant with Müller [22], the northern area was subdivided into the same four subareas. Silva & Casteleti [24] detected five AoEs of butterflies, birds and primates. Those areas were named Brejos Nordestinos, Pernambuco, Diamantina, Bahia and Serra do Mar. Brejos Nordestinos

are higher altitude forest spots~~,~~ surrounded by Caatinga ~~in Ceara~~, Pernambuco, Paraiba, and Piaui ~~states~~; Pernambuco is situated in the coastal region of occidental Northeast Brazil; Diamantina is located in the interior of the state of Bahia; Bahia extends from the state of Sergipe to the state of Espirito Santo; Serra do Mar comprises the mountain~~ous~~ areas from Rio de Janeiro to Rio Grande do Sul~~ states~~. The five AoE of Silva & Castelei [24] were delimited adjacent to three mixed transitional areas named São Francisco, Florestas do Interior and Florestas de Araucária. Silva *et al.* [36] proposed four AoEs using bird ranges: Pernambuco (as defined in previous studies); Bahia Central (~~correspondent with~~ Diamantina in Silva & Casteleti [24]); Bahia (homonym of Bahia AoE in Silva & Casteleti [24]); and Serra do Mar, the latter located on both sides of the Rio Doce, and extending from Espírito Santo to ~~the north of~~ Santa Catarina~~ states~~. Prado *et al.* [26] detected a single AoE of rodents in the AF region, the so-called Eastern South America, and subdivided it into four subregions. Each subregion would coincide with Pernambuco, Bahia, Diamantina and Serra do Mar AoEs of Silva & Casteleti [24], respectively. Pinto-da-Rocha *et al.* [37], DaSilva & Pinto-da-Rocha [38] and DaSilva *et al.* [25] detected 12 AoEs using harvestmen distributions. Only two out of the 12 ~~were coincident with~~ previously proposed AoEs, namely Pernambuco and Bahia. The remaining ten AoEs are small areas placed within AoEs mentioned in the literature. Hoffmeister *et al.* [19] detected 29 groups of AoEs. Some of these groups were congruent with previously delimited areas discovered by different authors. Based on orchid bee distributions, Garrafoni *et al.* [39] found at least five main AoEs, which are congruent with those from Silva & Casteleti [24], varying only in size.

AoEs are hypothesized areas that can vary depending on the data use [13, 27, 40-42]. By contributing with additional taxa, new distributional data and new historical reconstructions of unexplored taxonomic groups, progress can be achieved in AoE knowledge [28] and new AoE hypotheses can be generated. Terrestrial flatworms or land planarians (Platyhelminthes, Geoplanidae) are one of those unexplored taxonomic groups. No biogeographic studies have been

previously done using these animals. Terrestrial flatworms are very sensitive to modifications in the conditions of their environment [43], are highly endemic due to their low vagility and dependence on humidity [44, 45] and are endemic to relatively small areas [46]. Furthermore, recent taxonomic revisions revealed that land planarians have smaller distributions than what was previously thought, due to the discovery of cryptic species under the same name [47-49]. For these reasons, land planarians are considered an optimal taxonomic group to carry out biogeographic studies. This paper is aimed at contributing to the debate of discovery and test of congruence of areas of endemism in the Atlantic forest. For this purpose, we explored the distribution of land planarians across the AF through three methods, Endemicity Analysis (EA), Geographical Interpolation of Endemism (GIE), and a manual search.

Materials and methods

Study area comprises the areas covered with Atlantic forest between, and including the State of Espírito Santo (ES, the northernmost) and Rio Grande do Sul (RS, the southernmost). We compiled distributional data belonging to 270 species of geoplanids (Tab. 1; S1 Table). For these species, 403 records were obtained from the literature and 166 from our own data, summing up a total of 570 records in our dataset (S1 Table; S1 Material). Species with doubtful records or identification, from literature or our own data, were not considered (S2. Table). One record is here understood as an observation of a species (either from literature or from our own data) in certain place, independently from the number of times this species was found.

Tab. 1: Number of flatworm species restricted to Congruence Core (CC), including those with single record (CC SR sp.), Maximum Region of Endemism (MRE) and widespread, and results of numerical analyses (cell-sizes of NDM analyses and if it was found by GIE) of each ~~AE~~.

~~AE~~	CC	CC SR sp.	MRE	Endemic	Widespread	NDM	GIE
ORG	30	26	2	32	18	0.25, 0.5	Yes
SMSP	18	15	6	24	14	0.25, 0.3, 0.5	Yes
SSP	15	15	3	18	13	-	Yes
PR	10	10	1	11	9	-	Yes
NSC	12	10	0	12	18	0.1, 0.25	Merged with SSC
SSC	23	18	0	23	16	0.1, 0.25, 0.3	Merged with NSC
SFP	25	20	0	25	13	0.1, 0.25, 0.3	Merged with POA
POA	4	1	0	4	12	0.5	Merged with SFP
MIS	6	4	0	6	0	1.0	Yes
Total	143	119	12	155	120		

For our own data, we intensely sampled land planarians in six areas (Fig. 1) (Parque Estadual de Intervales, Parque Nacional de Saint-Hilaire/Lange, Pq. Nac. da Serra do Itajaí, Pq. Est. da Serra do

Tabuleiro, Pq. Nac. de São Joaquim and Floresta Nacional de São Francisco de Paula; ~200 person-hour of sampling), and sporadically in other localities between 2009 and 2018. Each of these intensely sampled areas ~~are~~ at a distance of ~120 km in a straight line from the next one. As a result, we collected 1621 specimens. These specimens were diagnosed by either only their external aspect (for known and characteristic species regarding size, shape and color of the body) or through external aspect and morphological details of the cephalic region, the pharynx and the copulatory apparatus examined on histological glass slides produced following Carbayo & Almeida [50] (S1 Table). 156 out of the 1621 specimens could not be identified either because of their poor state of conservation or due to their immature developmental status and were ~~no~~ further considered in this study. The remaining 1465 specimens belong to 94 known and 98 undescribed species, totalizing 192 species (S1 Table). We used GPS device Garmin E-Trex for recording the geographic coordinates with a maximum error of ~50 m of most of the specimens we collected. We used Googlemaps for recovering geographic coordinates of locations mentioned in the literature and a few of our own specimens which could not be georeferenced.

Fig. 1. Localities intensively sampled (~200 person-hour of sampling) in the Atlantic Forest.

To discover AoEs, the dataset (S1 Material) was analyzed using three methods: (i) the Endemicity Analysis (EA), which uses a heuristic algorithm of the software NDM/VNDM (version 3.1, developed by Goloboff [51], Szumik *et al.* [14] and complemented by Szumik & Goloboff [15]), (ii) the Geographical Interpolation of Endemism (GIE) based on a Kernel interpolation [18] and (iii) a manual search for locations with at least two endemic species. Resulted patterns were evaluated by combined criteria by DaSilva *et al.* [25], whose protocol was applied for a final delimitation.

The criteria used in method (i), NDM, were: (a) an area consisting of two or more grid cells presenting two or more endemic species (any species whose range is restricted to these or adjacent grid cells; see below), and (b) an endemicity score above two in each grid cell. We adjusted the

points of grid origin to -56.0 (X), -19.1 (Y), and subsequently run 100 replicas with each of five different grid cell sizes (0.1º, 0.25º, 0.3º, 0.5º, and 1º) to examine the effect of cell size on inferred patterns of endemism. We largely follow the protocol proposed by DaSilva *et al.*, [25] for several reasons discussed further below (see section *Discussion: 1. Methodological observations*). We computed consensus areas of endemism beginning, with 0.1º grid size cells, through a strict consensus or tight consensus rule [52, 53] using 5% similarity in species distribution between different sets of cells. Afterwards, we added the results obtained from analysis run with progressively larger grid cells (0.25º, 0.3º, 0.5º, and 1º) – sets ~~resulted~~ from larger grid cells are either the same as the smaller ones (but with coarser limits), the sums of two or more smaller ones or new areas [25]. Our complete dataset presents a number of gaps between sampled areas (Ex. S2-S9 Figure). Some of these gaps represent unsampled areas (empty cells in NDM/VNDM, S2-S9 Figure) and others represent real absences of certain species (brown cells in NDM/VNDM, S2-S9 Figure).

The second approach (ii), GIE, is based on a kernel interpolation of the species distribution, indicating level of endemism based on the area of influence of the species and their distribution overlap [18]. The spatial modeling was performed with the GIE toolbox provided by [18] in Esri ArcGis software 10.6 (http://www.esri.com). The interpolation is based on the definition of areas of influence around species centroid points. Said centroid points are calculated as arithmetic means of each species sampling coordinates [54]. Five categories of influence were determined based on the maximum distance between the centroid and the furthest point of occurrence for each species. Each category defines the radius of the area of influence around each species' centroid. The "influence" of each area decreases outwards, away from the centroid, according to a Gaussian function. The overlap between the areas of influence of all species was estimated by the kernel algorithm and the results were expressed in consensus normalized raster grids, representing relative levels of endemism. Although the establishment of the limits of classes and weight that can be given to each

class is in certain way arbitrary, the representation of areas of endemism as natural units with fuzzy boundaries is more compatible with the theoretical models of distribution of AoEs (see [13], [4]). In this study we performed a variation of said arbitrary units (categorization schemes) to analyze the influence they may have on AoE identification through GIE. The first analysis was obtained by analyzing the data, normalized (i.e. each of the five categories in the analysis had the same influence). The second analysis was done using five categories, with the predefined weight given by the GIE tool set [18]. In this analysis, species with smallest ranges weighted more (single record species (SR) not included in the consensus), progressively and exponentially losing weight in the successive categories. Species with a single record, and therefore present in a single locality, were named as 'Single Record' (abbreviated, SR).

A manual search method (iii) was carried out for single grid cells or single localities bearing two or more endemic species outside the AoE previously detected by methods (i) or (ii).

Species ranges that delimit or influence AoEs in the numerical analyses (i. e., NDM and GIE) were used to delimit the Congruence Cores (CC, [25]) of AoEs. Apart from species exclusively present within a CC, there may also occur species that do not contribute to the identification of a CC in the analyses. These species occur either in more than one CC, thus are defined and classified as widespread (WS), or in one CC and outside of it, but never within the limits of another CC. Latter situation is the basis for delimiting a 'Maximum Region of Endemism' (MRE) [25]. Finally, all species included in the study were classified within one of the four levels of endemicity (CC, MRE, WS, and SR, modified from [25]. Tab. 1). AoEs are thus delimited with a CC and an MRE, when applied in addition to the delimitations directly achieved by the numerical methods (NDM and GIE).

Results

Endemicity Analysis with NDM/VNDM - method (i)

The analysis employing the 0.1º cell size grid, produced four sets of preliminary AoEs. The consensus analysis of these sets generated the following three consensus-sets. The northernmost consensus set, named North Santa Catarina (NSC-NDM) (S2 Figure) is located in the north-east region of the state of Santa Catarina (Fig. 2A). It is defined by three species [*Choeradoplana langi* (Graff, 1894), *Obama assu* (Froehlich, 1959), *Pasipha velina* (Froehlich, 1959)] (latter defined using 0.25º-cells as well). Consensus-set of South Santa Catarina (SSC-NDM) (S3 Figure) is located in the south-east region of the state of Santa Catarina (Fig. 2A); it is defined by five species [*Choeradoplana abaiba* Carbayo *et al.*, 2017, *Obama nungara* Carbayo *et al.*, 2016, *Obama sp. 6, Paraba tingauna* (Kishimoto & Carbayo, 2012, *Pasipha sp. 10*), (last three species define with 0.25º-cells as well)]. The southernmost consensus-set identified is the São Francisco de Paula (SFP-NDM) (S4 Figure), located in the Floresta Nacional de São Francisco de Paula, in the state of Rio Grande do Sul (Fig. 2A). It is defined by five species [*Luteostriata arturi* (Lemos & Leal-Zanchet, 2008), *Luteostriata ceciliae* (Froehlich & Leal-Zanchet, 2003), *Obama josefi* (Carbayo & Leal-Zanchet, 2001), *Obama maculipunctata* Rossi *et al.*, 2016, *Paraba franciscana* (Leal-Zanchet & Carbayo, 2001)].

Fig. 2. NDM results obtained with A) 0.1º cell size grids. B) 0.25º cell size grids. C) 0.5º cell size grids. D) 1º cell size grids.

The analysis employing 0.25º cell size grid generated nine sets, and the consensus analysis generated six sets, namely the above-mentioned three sets using 0.1º-cells (obviously differing in the size, but defined by the same species each, respectively) plus two new sets. One of these new sets is the consensus-set of the Serra dos Órgãos (ORG-NDM) (S5 Figure), located in the Serra dos Órgãos, in the state of Rio de Janeiro (Fig. 2B). This set is defined by three species [*Cratera tamoia* (Froehlich, 1955), *Geoplana vaginuloides* (Darwin, 1844), *Obama fryi* (Graff, 1899) (the latter

species define with 0.5°-cells as well)]. The other consensus-set is the Serra do Mar de São Paulo (SMSP-NDM) (S6 Figure), located in the state of São Paulo (Fig. 2B). This set is defined by nine species [*Geoplana duca* Marcus, 1951, *Geoplana mogi* Almeida & Carbayo, 2019, *Obama livia* (Froehlich, 1954), *Obama metzi* (Graff, 1899) (define with 0.3°-cells as well), *Choeradoplana banga* Carbayo & Froehlich, 2012, *Choeradoplana marthae* Froehlich, 1955, *Obama braunsi* (Graff, 1899), *Obama evelinae* (Marcus, 1951) and *Obama schubarti* (Froehlich, 1957) (last five species define using 0.5º-cells as well)]. The remaining sets produced would be a disjunct AoE, which overlaps with other sets previously generated (SSC-NDM and SFP-NDM) (S7 Figure) and therefore presents WS species; thus, it cannot be considered an AoE.

Analysis run using 0.3º cell size grids generated sets of preliminary AoE all overlapping with sets generated with smaller cell size grids.

The analysis employing 0.5º cell size grid generated 19 sets, and the consensus analysis generated five sets four of which overlap sets previously generated by the other sized grid analyses plus only one new, named Porto Alegre (POA-NDM) (S8 Figure). This consensus-set is located in the metropolitan area of Porto Alegre, in the state of Rio Grande do Sul (Fig. 2C), and is defined by three species [*Luteostriata abundans* (Graff, 1899), *Paraba gaucha* (Froehlich, 1959) and *Pasipha hauseri* (Froehlich, 1959)].

The last analysis was run with 1º cell size grid. It generated thirty three sets from which the consensus analysis resulted in five sets. Four of these sets overlap existing sets previously generated and one set is new. This new consensus-set is named Misiones (MIS-NDM) (S9 Figure) and is located in the province of Misiones, Argentina (Fig. 2D). This set is defined by two species (*Pasipha mbya* Negrete & Brusa, 2016 and *Supramontana argentina* Negrete *et al.*, 2012).

In short, the seven AoEs identified through NDM, named after the geographical location of their consensus-sets, are as follows: Serra dos Órgãos (ORG-NDM), Serra do Mar de São Paulo (SMSP-

NDM), Northern Santa Catarina (NSC-NDM), Southern Santa Catarina (SSC-NDM), São Francisco de Paula (SFP-NDM), Porto Alegre (POA-NDM) and Misiones (MIS-NDM).

Geographical Interpolation of Endemism - method (ii)

Final products of this method are expressed in density surface grids. As expected, the first analysis (with normalized categories) generated an AoE as large as our sampling area housing less inclusive minor AoEs (Fig. 3).

Fig. 3. GIE results obtained with five normalized categories (each category in the analysis has the same influence) over a topographic map.

The second analysis (with five categories, default weights and species with SR excluded) (Fig. 4) showed major congruence with the results obtained through NDM/VNDM. This second analysis produced seven areas.

Fig. 4. GIE results obtained with five categories, with the predefined weight given by the GIE tool set (Oliveira *et al.*, 2015) over a topographic map. In this analysis, species with ~~smallest~~ ranges weighted more (single record species (SR) not included in the consensus), progressively and exponentially losing weight in the successive categories.

We therefore identify seven areas through GIE analysis. The northernmost area, named Serra dos Órgãos (ORG-GIE), is located in the Serra dos Órgãos, in the state of Rio de Janeiro. Said area is defined by some undetermined level of influence from 30 species (Tab. 1). Moving south, the next area identified is Serra do Mar de São Paulo (SMSP-GIE). It is located in the southeastern region of the state of São Paulo, and is defined by some level of influence from 28 species (Tab. 1). The next area identified by GIE analysis is Southern São Paulo (SSP-GIE), located further south in the state of São Paulo. It is influenced by 19 species (Tab. 1). The fourth area is Paraná (PR-GIE), located around Curitiba municipality, in the state of Paraná. Said area is influenced by 13 species (Tab. 1).

Continuing south, Santa Catarina (SC-GIE) is found occupying most of the eastern region of the state of Santa Catarina. This area is influenced by 77 species (Tab. 1). The area of São Francisco de Paula and Porto Alegre (SFP&POA-GIE), occupies a considerable region that extends from almost the southernmost region of the state of Santa Catarina to Porto Alegre's metropolitan area. It is defined by some influence of 34 species (Tab. 1). The last area identified is Misiones (MIS-GIE). Located in the province of Misiones in Argentina, this area is defined by some level of influence from six species (Tab. 1).

Summing up, the seven AoEs defined through GIE analysis are Serra dos Órgãos (ORG-GIE), Serra do Mar de São Paulo (SMSP-GIE), Southern São Paulo (SSP-GIE), Paraná (PR-GIE) Santa Catarina (SC-GIE), São Francisco de Paula and metropolitan area of Porto Alegre (SFP&POA-GIE), and Misiones (MIS-GIE). They were named based on their geographical location (Fig. 4).

Manual search - method (iii)

In addition to the numerical methods (NDM and GIE) above, eight additional localities were found to bear two or more endemic species – those recorded once for such localities (Fig. 5). From North to South, these localities are Santa Maria Madalena-RJ (two SR species), São José do Barreiro-SP (three SR species), Ubatuba-SP (four SR species), Matinhos-PR (four SR species), Três Barras-SC (two SR species), PN São Joaquim-SC (31 SR species), Derrubadas-SC (two SR species) and Santa Maria-RS (two SR species).

Fig. 5. Single localities bearing two or more endemic species identified through a Manual Search.

Summarized Results (Fig. 6)

Fig. 6. Topographical map showing AoEs identified in this study through the different three methodologies used.

NDM identified seven AoEs (ORG-NDM, SMSP-NDM, NSC-NDM, SSC-NDM, SFP-NDM,

POA-NDM and MIS-NDM) (Fig. 2). GIE identified seven AoEs (ORG-GIE, SMSP-GIE, SSP-GIE, PR-GIE, SC-GIE, SFP&POA-GIE and MIS-GIE) (Fig. 4). These areas can be summarized in nine AoEs of terrestrial flatworms in the Southern Atlantic forest. Three of them are found by both, NDM and GIE. Another four areas found by NDM are also found by GIE, but in GIE they are merged into two AoEs (the sums of NSC-NDM + SSC-NDM and POA-NDM + MIS-NDM). The last two AoEs were found only by GIE (PR-GIE and SSP-GIE). Alongside, the manual search identified eight additional AoEs based on single localities (Fig. 5).

CCs of these areas were delimited by the ranges of species that contributed to the score of NDM sets, drawing a line around those species ranges (Fig. 6. S3 Table). The CCs of the two AoEs found only by GIE (PR-GIE and SSP-GIE) were delimited by the single localities where endemic species have congruence (in both cases, only one species have more than one record) (Fig. 6). GIE does not indicate objectively the endemic species supporting an AoE (Escalante, 2016), as a high number of them influenced in each spot of the map (see above).

Eleven species do not occur in any CC so they are not classified within any of the levels of endemicity (CC, MRE, WS).

The results of both numerical analyses, the manual search and DaSilva´s protocol were summarized on a map of AoEs with CCs and MREs (Fig. 7). ORG was found by NDM and GIE and has a CC defined by three species (those that score in NDM). *Geoplana notophthalma* Riester, 1938 occurs in this CC but has a record in Minas Gerais state which consequently defines a MRE of ORG. SMSP was found by NDM and GIE and has a CC defined by four species (those that score in NDM with 0.25º and 0.3º cells). Five species occur in the CC and widens it to a MRE (*Choeradoplana marthae, Choeradoplana banga, Obama braunsi, Obama evelinae, Obama schubarti*). Those five species delimited a set in NDM with 0.5º cells, but actually do not have congruent ranges. This result is caused by an artifact of cell size and position, so we decided to consider them as delimiting

a MRE. PR and SSP were found only by GIE and are defined by a CC with a single locality each. SSP widens to MRE as defined by *Issoca potyra* Froehlich, 1958 and *Paraba tapira* (Froehlich, 1958) and PR widens to a MRE defined by *Notogynaphallia mourei* (Froehlich, 1956).

Fig. 7. Delimitation of AoEs of land planarians, on a topographical map of the Atlantic Forest region.

Discussion

1. Methodological observations

As seen in previous studies, cell size and grid origin have impacts on the results obtained from an NDM analysis. ~~Large cell size grids~~ generate less detailed hypotheses of species composition and area coverage of the AoEs [14, 55, 56], generating a higher number of AoEs, as can be seen in the present study. Usage of ~~large cell sizes~~ affects results, such as the collapse of two AoEs into a single one [25] or the inclusion of important barriers within a single AoE [57]. On the other hand, small grids introduce false absence of species, make distributions discontinuous, and present smaller number of species per cell, making it more difficult to detect ~~AoEs, and therefore defining less~~ of them [57]. Influences derived from cell size in revealing AoEs are alleviated by DaSilva's *et al.* [25] protocol. If only one ~~cell size~~ grid had been used or tested with our data, some AoEs would have been missed through NDM analysis.

On the other hand, GIE analysis includes another "scaling effect" similar to the ~~cell size~~ grid dilemma mentioned before. There are two arbitrarily chosen units in GIE analysis. One unit is the number of categories of influence to be used in the analysis. The other is the values limiting the influence of each category (the relative weight each class will have in the analysis). Tests varying the number of categories have shown that modifying this unit~~,~~ will not affect significantly the

results obtained via GIE analysis [18], although a high number of categories will definitely affect the analysis, identifying AoEs with higher discontinuity and also showing “micro areas” which sometimes are composed by a single species, overestimating the amount of AoEs detected (following Platnick’s [27] definition of an AoE, a single endemic species should not be able to define AoEs) (S1 Figure). Therefore the chosen categorization scheme should be tested and evaluated for every dataset analysed in order to evade defining highly discontinuous AoEs or overidentifying the number of AoEs.

The dataset analyzed with normalized weighted categories (all weights are the same for every category) (Fig. 2) the result obtained is a single AoE comprising the whole study area with minor areas defined inside. This analysis is unable to identify AoEs (AoEs identified in this study by other GIE categorization schemes and NDM) such as ORG, POA or MIS. The reason for this is the fact that the influence value of species with very restricted distributions is the same as the influence value of widespread species. When trying to identify AoEs, species with restricted ranges are much more indicative than widespread species. Therefore, GIE analysis should be carried out with weighted categories.

The categorization scheme chosen for GIE in this study consisted of five weighted categories (default weights in the ArcGIS toolbox). SR species were not included in the consensus of this analysis to evade the identification of areas with one SR species and to try and minimize the effect of poorly sampled species on AoE identification (see [58]). As NDM does not take in account SR species, this analysis shows high congruence with NDM results.

GIE analysis overcomes several problems generated from the use of grid cells [18]. It allows the use of distributional data with gaps and offers the possibility of identifying AoEs with fuzzy edges which corresponds more with the spatial and temporary reality of AoEs [4, 18]. In contrast, it does not show objectively the limits of an AoE, because of this fuzzy edges.

GIE fails to provide a set of species diagnosing the ~~AoEs identified~~ [59] apart from the "scaling effect" derived from the arbitrary units chosen for the categorization scheme. The identification of an AoE should not be possible if the list of species diagnosing that area cannot be obtained. An approximate list of species influencing a specific AoE can be obtained with GIE, but those species cannot be classified as diagnostic species because numerically it is not possible to know the influence of each species in each ~~AoE identified~~.

No method, neither NDM or GIE, is able to perform a complete analysis to identify AoEs~~,~~ due to the different flaws each methodology presents [25, 57, 60]. ~~Analysis~~ perform differently, offering the possibility to detect AoEs based on different methodological approaches. Therefore the most efficient way to work when trying to detect AoEs is through the integration of different methodologies as seen in previous studies [18, 20, 25, 39]. By working with different numerical algorithms, functions and formulas and different theoretical and methodological approaches, we are able to analyze our databases with a broader spectrum and correlate our practical results to the reality of endemic species distribution and congregation~~,~~ with a higher confidence.

DaSilva's protocol facilitates the integration of different methodologies when identifying AoEs. The use of a Congruence Core (CC) to delimit the main area of endemic species congruence permits the inclusion of results obtained through other methodologies efficiently and avoids the artifacts caused by grid cells, fuzzy edges or ~~any else~~. Defining Maximum Region of Endemism (MRE) also sets the concept that an AoE does not occupy~~, uniquely,~~ the congruent space between endemic species, but the outside areas which might represent the dynamism of said endemic species as well [25, 61]. DaSilva's protocol also maximizes EA's performance by including several steps that reduce the grid-size effect and by discarding a high number of resulted sets caused by several combinations of overlapping ranges and consequent redundancy between them. DaSilva's protocol is focused on grid-based methods, but can be an effective tool to integrate results obtained from other methodologies that obviate grids as well.

Species with a single record (SR) were included in this study, even though they are often not considered in endemism analyses. NDM only considers a species if it occurs in two or more cells, thus a cell with many endemic species is not detected by the method as an AoE. GIE overcomes this problem, but is unable to differentiate localities with just one species record from localities with two or more species records, as mentioned above. Even though some methods have no use for SR species, we believe ~~said~~ species represent important biological information for planarian distribution and diversity~~,~~ and therefore should be included.

2. Inferring AoEs based on land planarian distributions

Our dataset revealed nine AoEs in total, plus eight localities with two or more SR species. Two AoEs (SSP and PR) could not be detected by our EA. These AoEs (SSP and PR) present an extremely high number of endemic species (19 and 18 species, respectively). These two AoEs were not detected due to the fact that most species endemic to them are known from one locality~~, thus not being~~ considered by NDM, because the method only considers sets with two cells or more [25, 57].

As mentioned before, some species distributions do not completely overlap the CCs. Those species present in only one CC and outside of it will define a MRE. They can be interpreted as an extension from a CC due to ontological causes~~,~~ or~~,~~ as the source of an error such as ~~under sampled~~ regions. Most of the identified AoEs do not have a MRE because planarian records are very concentrated in specific localities and regions, and those localities and regions were each delimited as an AoE~~s~~ (see discussion below). Only four AoEs delimited MREs: ORG, SMSP, SSP and PR. Some of the species defining MREs create doubtful AoEs. Such is the case of *Geoplana notophthalma* which defines a distant portion of ORG's MRE. ~~Said~~ distant portion of MRE is a CC expanded northwards to the Nova Lima municipality in the state of Minas Gerais. This MRE is a peculiar configuration for an AoE as AoEs are defined by the congruence of only species with small distributions [62]. Widespread species cannot define an AoE, ~~though,~~ *G. notophthalma* cannot be considered a

widespread species as it is not found in any other AoE, apart from ORG. Thus, *G. notophthalma* is found in the geographic region of Serra dos Órgãos and in Minas Gerais, and must expand ORG's AoE's borders through a MRE so giving rise to a disjunct AoE. Froehlich [63] suggested this species to be synonym of *O. applanata* (Graff, 1899), but the situation would not change since their distribution is the same.

SMSP has a MRE extending to the south and interior of the state, presenting many species ranges not congruent among them. This AoE has a more complete MRE because it was delimited in a region with more sampling history and knowledge of planarian fauna and therefore, geographically more covered up. PR is composed of 10 species in a single locality and a single species occurring inside and outside said locality, defining the MRE; and SSP bares 15 species in a single locality and two species occurring inside and in two other localities - they compose the MRE.

Each of the six intensively sampled areas is at a distance of 120 km from the nearest intensively sampled area and, interestingly, each one was revealed as an AoE. This situation rises a question: if we had sampled between these intensively sampled areas would we have discovered additional AoEs? In other words, geographic distribution of most land planarians might occur at a scale smaller than areas with 120 km in length. General assumptions of endemism have been proposed and explained as a consequence of the ecological and physiological limitations of these organisms [43, 46, 64]. If this is true, further sampling of these organisms is promising in revealing new AoEs within the study area even so because most land planarians have revealed to present a very small distributional range and each of the six areas intensely sampled turned out to be an AoE.

3. Causal factors of areas of endemism

In general, Serra do Mar, the mountains spreading almost along the whole geographic range herein analyzed, is considered a single area of endemism for forest-dependent vertebrate and plant taxa [22, 24, 65, 66]. However, some studies have shown a more endemic pattern of distribution for

species or populations in the southeastern-south coastal mountains and adjacent lowlands of Atlantic Forest, as seen with the area of endemism of harvestmen [25] and spiders [18], specific distributional congruence of vertebrates [67], ecological regionalization of frogs [68], and phylogeographic structure of species of frogs [69-72], bees [73], vipers [74], birds [75, 76], mammals [77, 78] and harvestmen [79, 80]. The main divergence is found to the south of ~~São Paulo state~~ or close to Ribeira do Iguape river valley [6, 67, 71, 73, 74, 76], but with very different times of divergence between those taxa (~ 0.39–4.9 m.y.a.) and weak inference of causal processes.

The planarian species show very restricted ranges only compared to harvestmen species [25], or maybe to some frog or cricket taxa [81, 82]. They seem to be restricted to more humid portions of the forest on mountain slopes or adjacent regions, where the core of their areas of endemism is located, with few exceptions. Main valleys or sedimentary basins seem to be the barriers for those taxa, as described for Paraíba do Sul river and Ribeira do Iguape river [64, 67, 71, 73, 74, 76, 79, 80].

The Atlantic Forest is not physiognomically homogeneous. It presents humid forests concentrating on slopes or medium altitudes of mountains. The valleys and coastal plains are composed by a marine influenced vegetation, and interior lowlands of the south-southeastern region ~~composed~~ by seasonal semideciduous forests. The endemic patterns found herein and for harvestmen show a restriction in more humid forests. This could be directly influenced by past events of forest reduction and posterior concentration in those regions, or refuges. A great body of literature from different sources and proxies continues to furnish evidence for reduction of forests in cycles during the Neogene (e.g. [70, 75, 83-85]). The high endemicity found for planarians seems to be influenced by those reductions, since PR x SC x SFP x POA and ORG x SMSP x SSP do not have clear geographic barriers. In this view, in the regions of those AoE cores, the forest should have been more humid than in lowland areas and consequently should have been maintained with few alterations during climatic fluctuations [25, 83, 86, 87].

The same should have happened to sedimentary basins wherein the great rivers flow. Those basins were originated by tectonism related with uprising of mountain ranges of Serra do Mar and Serra da Mantiqueira and subsidence of valleys since at least the Miocene [88-90]. In fact, the southeastern region of the Atlantic Forest is considered as the main rift system of eastern South America [89]. Some of those valleys started to be rain shadows as they are hidden from orographic rains and coastal humidity behind the Serra do Mar mountains (e.g. Paraíba do Sul river, [91]). Rivers originated as they flowed to the subsidence regions and most of their heads were captured from a western direction, eastward to the coast [92]. Sedimentation of those regions was affected by neotectonic reactivation [90] and rising of the amount of flowing water in more humid periods, even forming paleolakes [89] or by marine transgressions into coastal valleys [93-95]. Then, many associated events feedbacked sedimentary basins as barriers for forest-dependent taxa, avoiding the development of humid forests for long periods. They resulted in many taxa splitting in these lowlands and valley regions but in different times between each other. DaSilva *et al.* [6] proposed a model of reiterative barriers with spatial congruence in multiple times to explain those patterns.

Álvarez-Presas *et al.* [64] have shown a phylogeographic structure for *Cephaloflexa bergi* planarian populations, congruent to those main barriers, Paraíba do Sul river and Ribeira do Iguape river valleys, and a split in northern São Paulo/south Rio de Janeiro states. There is not a clear geographic barrier associated to the latter, but is congruent to ORG X SMSP AoEs, cited above, the same for harvestmen AoEs [25], and phylogeographic structure for *Promitobates* harvestmen [79]. The dating estimates for those main splits of *Cephaloflexa* planarian is about 8 m.y.a., in the Miocene period, near to the *Promitobates* harvestmen splits estimated in about 11 m.y.a. It is important to note that possible reductions of forest caused by climatic desiccation probably occurred previously to the renowned and more recorded Pliocene/Pleistocene fluctuations [96-98]. In spite of lack of phylogeographic structure of some populations of *Cephaloflexa*, Alvares-Prezas et al. [64] assumed that populations remained separated by those main barriers and there were few

more recent dispersions.

POA and MIS areas have a different character related to their physical conditions. It is assumed that those forests have seasonal deciduous physiognomies [99] in contrast to the humid evergreen forests of the other areas of endemism. These endemisms could be associated to those species ecological requirements or isolation from the ~~around~~ open vegetations. However, MIS ~~seem~~ to be related to a separation at Paraná river or its tributary valleys, since they flow on an important sedimentary basin, as discussed above.

Based on this discussion, the hypotheses for the causes of the presented endemic patterns, inferred as speciation processes between endemic species living in those areas of endemism, should be tested or deepened by a phylogenetic framework with temporal estimation of each cladogenesis. The corroboration of the phylogeographic structure and the main populational divergence shown by Ávarez-Presas *et al.* [64] and the areas of endemism of hundreds of planarians species contribute to the understanding of the biogeographical evolution of those animals and consequently, of the humid Atlantic Forest.

Conclusions

In this paper, we corroborate DaSilva's *et al.*, (2015) protocol as an effective tool to aid in the discovery of AoEs. ~~Said~~ protocol was used with two numerical methodologies, namely an EA (grid-based NDM) and a GIE (centroid-based). The protocol exploits the advantages of each numerical ~~methodologies~~ (NDM allows the identification of species defining an AoE and permits a more accurate delimitation of an AoE; GIE even detects AoEs ~~constituted by~~ a single record), whereas the manual search of species allowed us to detect a number of AoEs previously not detected, and species occurring within a single AoE and outside of it, delimiting an MRE.

The AoEs of land planarians discovered here are congruent with fine-scale approaches such as that

addressed by DaSilva *et al.* (2015) for harvestmen. Land planarians and harvestmen are hygrophilous and forest-dependent organisms and present sensitivity to environmental changes. Presumably, due to these common physiological and ecological constraints, harvestmen and land planarians may predict, more robustly, a common history of other restricted taxa of the Atlantic Forest. Furthermore, new samplings in unexplored localities in the study area might indeed uncover new endemisms and additional AoEs. Hence, these organisms might be a good model for fine-scale studies of AoEs.

List of Abbreviations

AF: Atlantic Forest

AoE: Area of endemism

ES: State of Espírito Santo

RS: State of Rio Grande do Sul

RJ: State of Rio de Janeiro

SP: State of São Paulo

PR: State of Paraná

SC: State of Santa Catarina

Pq. Nac.: Parque Nacional (National Park)

Pq. Est.: Parque Estadual (State Park)

EA: Endemicity Analysis (same as NDM)

NDM: Endemicity Analysis (same as EA)

NDM/VNDM: Visualization NDM (software displaying NDM Analysis)

GIE: Geographic Interpolation of Endemism

SR species: Single Record species

CC: Congruence Core

MRE: Maximum Region of Endemism

WS: Widespread

NSC-NDM: Area of endemism of North Santa Catarina (in EA/NDM)

SSC-NDM: Area of endemism of South Santa Catarina (in EA/NDM)

SFP-NDM: Area of endemism of São Francisco de Paula (in EA/NDM)

ORG-NDM: Area of endemism of the Serra dos Órgãos (in EA/NDM)

SMSP-NDM: Area of endemism of the Serra do Mar de São Paulo (in EA/NDM)

POA-NDM: Area of endemism of Porto Alegre (in EA/NDM)

MIS-NDM: Area of endemism of Misiones (in EA/NDM)

ORG-GIE: Area of endemism of the Serra dos Órgãos (in GIE Analysis)

SMSP-GIE: Area of endemism of Serra do Mar de São Paulo (in GIE Analysis)

SSP-GIE: Area of endemism of Southern São Paulo (in GIE Analysis)

PR-GIE: Area of endemism of Paraná (in GIE Analysis)

SC-GIE: Area of endemism of Santa Catarina (in GIE Analysis)

SFP&POA-GIE: Area of endemism of São Francisco de Paula and Porto Alegre (in GIE Analysis)

MIS-GIE: Area of endemism of Misiones (in GIE Analysis)

ORG: Area of endemism of the Serra dos Órgãos

SMSP: Area of endemism of Serra do Mar de São Paulo

SSP: Area of endemism of Southern São Paulo

PR: Area of endemism of Paraná

NSC: Area of endemism of North Santa Catarina

SSC: Area of endemism of South Santa Catarina

SFP: Area of endemism of São Francisco de Paula

POA: Area of endemism of Porto Alegre

MIS: Area of endemism of Misiones

Acknowledgements

We thank Instituto Chico Mendes de Conservação da Biodiversidade (ICMBio), Instituto Florestal do Governo do Estado de São Paulo (IF), Museu de Zoologia da Universidade de São Paulo (MZUSP), Fundação do Meio Ambiente do Governo Estado de Santa Catarina (FATMA) for sampling licence in Parque Nacional da Serra dos Órgãos, Parque Nacional Saint Hilaire/Lange, Parque Nacional da Serra de Itajaí, Floresta Nacional de São Francisco de Paula (ICMBio); Parque Estadual Intervales (IF), Parque Estadual da Serra do Tabuleiro (FATMA). We are indebted to Ana Laura Almeida, Claudia Tangerino Olivares, Júlio Pedroni, Débora Redivo, Karine Gobetti, Leonardo Zerbone, Marília Jucá, Marta Álvarez Presas, and Welton Araujo for sampling help; Ítalo Silva de Oliveira Sousa, Geison Castro da Silveira, and Lucas Beltrami for the histological work; and K. Gobetti for the help with histological slides of E. M. Froehlich collection.

References

1. Morrone JJ, Crisci JV. Historical biogeography: introduction to methods. Annu. Rev. Ecol. Syst. 1995;26: 373–401.
2. Henderson IM. Biogeography without area? Austral Syst Bot. 1991;4: 59.
3. Cracraft J. Historical biogeography and patterns of differentiation within the South American avifauna: areas of endemism. Ornithol. Monogr. 1985;36: 49–84.
4. Crother BI, Murray CM. Ontology of areas of endemism. J Biogeogr. 2011;38(6): 1009-1015.

5. Morrone JJ. Biogeographical regionalisation of the Neotropical Region. Zootaxa. 2014;3782(1): 001–110.
6. DaSilva MB, Pinto-da-Rocha R, and Morrone JJ. Historical relationships of areas of endemism of the Brazilian Atlantic rain forest: a cladistic biogeographic analysis of harvestman taxa (Arachnida: Opiliones). Curr Zool. 2016;63(5): 525-535.
7. Gámez N, Nihei SS, Scheinvar E, Morrone JJ. A temporally dynamic approach for cladistic biogeography and the processes underlying the biogeographic patterns of North American deserts. J Zoolog Syst Evol Res. 2014. https://doi.org/10.1111/jzs.12142.
8. Vane-Wright RI, Humphries CJ, Williams PH. 1991. What to protect? Systematics and the agony of choice. Biol Conserv. 1991;55: 235–254. https://doi.org/10.1016/0006-3207(91)90030-d
9. Rodrigues AS, Orestes Cerdeira J, Gaston KJ. Flexibility, efficiency, and accountability: Adapting reserve selection algorithms to more complex conservation problems. Ecography. 2000;23: 565–574. https://doi.org/10.1111/j.1600-0587.2000.tb00175.x
10. Díaz Gómez JM. Estimating ancestral ranges: Testing methods with a clade of Neotropical lizards (Iguania: Liolaemidae). PloS One. 2011;6: e26412. https://doi.org/10.1371/journal.pone.0026412
11. Martínez-Hernández F, Mendoza-Fernández AJ, Pérez-García FJ, Martínez-Nieto MI, Garrido-Becerra JA, Salmerón-Sánchez E, Merlo ME, Gil C, Mota JF. (2015) Areas of endemism as a conservation criterion for Iberian gypsophilous flora: a multi-scale test using the NDM/VNDM program. Plant Biosyst. 2015;149(3): 483-493. https://doi.org/10.1080/11263504.2015.1040481
12. Rosen BR. From fossils to earth history: Applied historical biogeography. . In: Myers A, Giller P, editors. Analytical biogeography: An integrated approach to the study of animal and plant distribution. Chapman and Hall, London; 1988. p. 437-481.

13. Morrone JJ. On the identification of Areas of Endemism. Syst. Biol. 1994;43: 438–441.
14. Szumik C, Cuezzo F, Goloboff PA, Chalup A. An optimality criterion to determine areas of endemism. Syst Biol. 2002;51: 806–816.
15. Szumik C, Goloboff PA. Areas of endemism: an improved optimality criterion. Syst Biol. 2004;53: 968–977.
16. Porzecanski AL, Cracraft J. Cladistic analysis of distributions and endemism (CADE): Using raw distributions of birds to unravel the biogeography of the South American arid lands. J Biogeogr. 2005;32: 261–275. http://dx.doi.org/10.1111/j.1365-2699.2004.01138.x
17. Dos Santos DA, Fernández HR, Cuezzo MG, Domínguez E. Sympatry inference and network analysis in biogeography. Syst. Biol. 2008;57: 432–448.
18. Oliveira U, Brescovit AD, Santos AJ. Delimiting Areas of Endemism through Kernel Interpolation. PLoS ONE. 2015;10(1): e0116673.
19. Hoffmeister CH, Ferrari A. Areas of endemism of arthropods in the Atlantic Forest (Brazil): an approach based on a metaconsensus criterion using endemicity analysis. Biol J Linn Soc. 2016;119(1): 126–144. https://doi.org/10.1111/bij.12802
20. Morales-Guerrero A, Miranda TP, Marques AC. Comparison between Parsimony Analysis of Endemicity (PAE), Endemicity Analysis (EA), and an alternative coding of Three-Distribution Statements based on hypothetical distributions. Syst Biodivers. 2017;15(5): 391-398. https://doy.org/10.1080/14772000.2016.1257519
21. Salinas NR, Wheeler WC. Statistical Modeling of Distribution Patterns: A Markov Random Field Implementation and Its Application on Areas of Endemism. Syst. Biol. 2019;0(0): 1–15. https://doi.org/10.1093/sysbio/syz033
22. Müller P. The dispersal centres of terrestrial vertebrates in the Neotropical realm: a study in the evolution of the Neotropical biota and its native landscape. Biogeographica 2. 1973; 1–250.

23. Kinzey WG. 1982. Distribution of primates and forest refuges. In: Prance GT, Editor. Biological diversification in the tropics. Columbia University Press, New York, New York; 1982. p. 455-482.
24. Silva JMC, Casteleti CHM. Estado da biodiversidade da Mata Atlântica brasileira. In: Galindo–Leal C, Câmara IG, editors. Mata Atlântica: Biodiversidade, Ameaças e Perspectivas. 2005.
25. DaSilva MB, Pinto-da-Rocha R, DeSouza AM. A protocol for the delimitation of areas of endemism and the historical regionalization of the Brazilian Atlantic Rain Forest using harvestmen distribution data. Cladistics. 2015;31: 692-705.
26. Prado JR, Brennand PGG, Godoy LP, Libardi GS, Edson, Abreu-Jr F, Roth PRO, Chiquito EA, Percequillo AR. Species richness and areas o endemism of oryzomyine rodents (Cricetidae, Sigmodontinae) in South America: an NDM/VNDM approach. J Biogeogr. 2015;42: 540–551.
27. Platnick NI. On areas of endemism. Aust. Syst. Bot. 1991;4: 11-12.
28. Morrone JJ. Homology, biogeography and areas of endemism. Divers. Distrib. 2001;7: 297–300.
29. Morellato LPC, Haddad CFB. Introduction: The Brazilian Atlantic Forest. Biotropica. 2000;32(4b): 786-792.
30. Leitão-Filho HF, Morellato LPC. Semideciduous forests of Southeastern Brasil – Serra do Japi. In: Davis SD, Heywood VH, Herrera-MacBryde O, Villa-Lobos J, Hamilton AC, editors. Centres for plant diversity: a guide and strategy for their conservation. IUCN, WWF, Washington; 1997. p. 381-384.
31. Câmara IG. Brief history of conservation in the Atlantic Forest. In: Galindo-Leal C, Câmara IG, editors. The Atlantic Forest of South America: Biodiversity Status, Threats, and Outlook. CABS and Island Press, Washington; 2003. p. 31–42.

32. Brown K, Jil S, Brown GG. Habitat alteration and species loss in Brazilian forests. In: Whitmore TC, Sayer JA, editors. Tropical deforestation and species extinction. Chapman and Hall, London, England; 1992. p. 119-142.
33. Wallace AR. On the monkeys of the Amazon. Proc. Zool. Soc. Lond. 1852;20: 107– 110.
34. Amorim DS, Pires MRS. Neotropical biogeography and a method for a maximum biodiversity estimation. In: Bicudo CEM, Menezes NA, editors. Biodiversity in Brazil. A First Approach. Conselho Nacional de Desenvolvimento Cientifico e Tecnologico, São Paulo; 1996. p. 183-219.
35. Costa LP, Fonseca GAB, Fonseca MT. Biogeography of South America forest mammals: endemism and diversity in the Atlantic Forest. Biotropica. 2000;32: 872-881.
36. Silva JMC, Sousa MC, Castelletti CHM. Areas of endemism for passerine birds in the Atlantic forest, South America. Glob Ecol Biogeogr. 2004;13: 85-92.
37. Pinto-da-Rocha R, DaSilva MB, Bragagnolo C. Faunistic similarity and historical biogeography of the harvestmen of southern and southeastern Atlantic Rain Forest of Brazil. J. Arachnol. 2005;33: 290–299.
38. DaSilva MB, Pinto-da-Rocha R. A historia da Mata Atlantica atraves da distribuicão dos opiliões (Arachnida). In: Carvalho CJB, Almeida EAB, editors. Biogeografia da America do Sul: Padrões e Processos. Editora Roca, São Paulo; 2011. p. 221–238.
39. Garrafoni ARS, Moura FR, Lourenco AP. Areas of endemism in the Atlantic Forest: quantitative biogeography insights from orchid bees (Apidae: Euglossini). Apidologie. 2017;48: 513. https://doi.org/10.1007/s13592-017-0494-6
40. Espinosa D, Aguilar C, Escalante T. Endemismo, áreas de endemismo y regionalización biogeográfica. In: Llorente J, Morrone JJ, editors. Introducción a la biogeografía en Latinoamérica: teorías, conceptos, métodos y aplicaciones. México D.F: Las Prensas de Ciencias; 2001. p. 3137.

41. Linder P. On areas of endemism, with an example from the African Restionaceae. Syst. Biol. 2001;50: 892–912.

42. Carine MA, Humphries CJ, Guma IR, Reyes-Betancort JA, Guerra AS. Areas and algorithms: evaluating numerical approaches for the delimitation of areas of endemism in the Canary Islands archipelago. J Biogeogr. 2009;36: 593–611.

43. Carbayo F, Leal-Zanchet AM, Vieira EM. Terrestrial flatworm (Platyhelminthes: Tricladida: Terricola) diversity vs. man-induced disturbance in a subtropical rainforest from Southern Brazil. Biodivers Conserv. 2002;11: 1091-1104.

44. Sluys R. Platyhelminthes as paleogeographical indicators. Hydrobiologia. 1995;305: 49-53.

45. Winsor L, Johns PM, Yeates GW. Introduction, ecological and systematic background to the Terricola (Tricladida). Pedobiologia. 1998;42: 389-404.

46. Sluys R. Global diversity of land planarians (Platyhelminthes, Tricladida, Terricola): a new indicator-taxon in biodiversity and conservation studies. Biodivers Conserv. 1999;8: 1663–1681.

47. Lemos VS, Cauduro GP, Valiati VH, Leal-Zanchet AM. Phylogenetic relationships within the flatworm genus Choeradoplana Graff (Platyhelminthes : Tricladida) inferred from molecular data with the description of two new sympatric species from Araucaria moist forests. Invertebr Syst. 2014;28: 605–627. http://dx.doi.org/10.1071/IS14003

48. Almeida AL, Marques FPL, Carbayo F. ‘Endless forms most beautiful’: taxonomic revision of the planarian Geoplana vaginuloides (Darwin, 1844) and discovery of numerous congeners (Platyhelminthes: Tricladida). Zool J Linnean Soc. 2018;20: 1–65.

49. Lago-Barcia D, Carbayo F. Taxonomic revision of four species of the Neotropical land planarian genera Cratera and Geoplana (Platyhelminthes, Tricladida) with a description of two new species and an emendation of Cratera. Zootaxa. 2018;4500(4): 517–542

50. Carbayo F, Almeida AL. Anatomical deviation of male organs of land planarians from Rio de Janeiro, Brazil, with description of two new species of Cratera (Platyhelminthes, Tricladida). Zootaxa. 2015;3931(1): 027–040. http://dx.doi.org/10.11646/zootaxa.3931.1.2
51. Goloboff P. NDM and VNDM: Programs for the identification of areas of endemism, vers. 1.6. 2002.
52. Szumik C, Casagranda D, Roig-Junent S. Manual de NDM/VNDM: programas para la identificación de áreas de endemismo. Inst. Arg. Est. Filogenet. 2006;5(3):1–26.
53. Aagesen L, Szumik C, Goloboff P. Consensus in the search for areas of endemism. J Biogeogr. 2013;40: 2011–2016.
54. Franklin J. Mapping species distributions: spatial inference and prediction. Cambridge University Press. 2010.
55. Szumik C, Aagesen L, Casagranda D, Arzamendia V, Baldo D, Claps LE, Cuezzo F, Gomez JMD, Giacomo AD, Giraudo A, Goloboff P, Gramajo C, Kopuchian C, Kretzchmar S, Lizarralde M, Molina A, Mollerach M, Navarro F, Nomdedeu S, Panizza A, Pereyra VV, Sandoval M, Scrocchi G, Zuloaga FO. Detecting areas of endemism with a taxonomically diverse data set: plants, mammals, reptiles, amphibians, birds and insects from Argentina. Cladistics. 2012;1: 1–13.
56. Aagesen L, Szumik C, Zuloaga FO, Morrone O. Biogeography of the South America highlands—recognizing the Altoandina, Puna, and Prepuna through the study of Poaceae. Cladistics. 2009;25: 295–310.
57. Moline PM, Linder HP. Input data, analytical methods and biogeography of Elegia (Restionaceae). J. Biogeogr. 2006;33: 47– 62.
58. Casagranda MD, Goloboff PA. On stability measures and effects of data structure in the recognition of areas of endemism. Biol. J. Linn. Soc. 2019;20: 1–13.

59. Escalante T. A natural regionalization of the world based on primary biogeographic homology of terrestrial mammals. Biol. J. Linn. Soc. 2016;120(2): 349–362. https://doi.org/10.1111/bij.12898.
60. Nihei SS. Misconceptions about parsimony analysis of endemicity. J Biogeogr. 2006;33: 2099–2106.
61. Noguera-urbano EA. Areas of endemism: travelling through space and the unexplored dimension. Syst. Biol. 2016;14(2): 131-139. https://doy.org/10.1080/14772000.2015.1135196
62. Deo AJ, DeSalle R. Nested areas of endemism analysis. J. Biogeogr. 2006;33: 1511–1526.
63. Froehlich CG. (1956). Tricladida Terricola das regi~oes de Teresopolis e Ubatuba. Pap Avulsos Zool. 1956;12: 313–344.
64. Álvarez-Presas M, Sánchez-Gracia A, Carbayo F, Rozas J, Riutort M. Insights into the origin and distribution of biodiversity in the Brazilian Atlantic forest hot spot: a statistical phylogeographic study using a low-dispersal organism. Heredity. 2014;112: 656–665. https://doi.org/10.1038/hdy.2014.3
65. Prance GT. Forest refuges: evidence from woody angiosperms. In: Prance GT, editor. Biological Diversification in the Tropics. Columbia University Press, New York, NY; 1982. p. 137–158.
66. Soderstrom TR, Judziewicz EJ, Clark LG. Distribution patterns of Neotropical bamboos. In: Vanzolini PE, Heyer WR, editors. Proceedings of a Workshop on Neotropical Distribution Patterns. Academia Brasileira de Ciências, Rio de Janeiro; 1988. p. 121–157.
67. Silva SM, Moraes-Barros N, Ribas CC, Ferrand N, Morgante JS. Divide to conquer: a complex pattern of biodiversity depicted by vertebrate components in the Brazilian Atlantic Forest. Biol. J. Linn. Soc. 2012;107: 39–55.

68. Vasconcelos TS, Prado VHM, Silva FR, Haddad CFB. Biogeographic Distribution Patterns and Their Correlates in the Diverse Frog Fauna of the Atlantic Forest Hotspot. PLoS ONE. 2014;9(8): e104130.

69. Fitzpatrick SW, Brasileiro CA, Haddad CFB, Zamudio KR. Geographical variation in genetic structure of an Atlantic Coastal Forest frog reveals regional differences in habitat stability. Mol Ecol. 2009;18: 2877-2896.

70. Carnaval AC, Hickerson MJ, Haddad CFB, Rodrigues MT, Moritz C. Stability predicts genetic diversity in the Brazilian Atlantic Forest Hotspot. Science. 2009;323: 785-789.

71. Brunes TO, Sequeira F, Haddad CFB, Alexandrino J. Gene and species trees of a Neotropical group of treefrogs: genetic diversification in the Brazilian Atlantic Forest and the origin of a polyploid species. Mol. Phylogen. Evol. 2010;57: 1120-1133

72. Amaro RC, Rodrigues MT, Yonenaga-Yassuda Y, Carnaval AC. Demographic processes in the montane Atlantic rainforest: molecular and cytogenetic evidence from the endemic frog *Proceratophrys boiei*. Mol. Phylogenetics Evol. 2012;62: 880-888.

73. Batalha-Filho H, Waldschmidt AM, Campos LAO, Tavares MG, Fernandes-Salomão TM. Phylogeography and historical demography of the Neotropical stingless bee *elipona quadrifasciata* (Hymenoptera, Apidae): incongruence between morphology and mitochondrial DNA. Apidologie. 2010;41: 534-547.

74. Grazziotin FG, Monzel M, Echeverrigaray S, Bonatto SL. Phylogeography of the B*othrops jararaca* complex (Serpentes: Viperidae): past fragmentation and island colonization in the Brazilian Atlantic Forest. Mol Ecol. 2006;15: 3969-3982.

75. D'Horta FM, Cabanne GS, Meyer D, Miyaki CY. The genetic effects of Late Quaternary climatic changes over a tropical latitudinal gradient: diversification of an Atlantic Forest passerine. Mol Ecol. 2011;20: 1923-1935.

76. Cavarzere V, Silveira LF, Vasconcelos MF, Grantsau R, Straube FC. Taxonomy and biogeography of Stephanoxis Simon, 1897 (Aves: Trochilidae). Pap Avulsos Zool. 2014;54: 69-79.
77. Mustrangi MA, Patton JL. Phylogeography and systematics of the Slender Mouse Opossum Marmosops (Marsupialia: Didelphidae). University of California Publications in Zoology. 1997;130.
78. Martins FM, Templeton AR, Pavan ACO, Kohlbach BC, Morgante JS. 2009. Phylogeography of the common vampire bat (*Desmodus rotundus*): Marked population structure, Neotropical Pleistocene vicariance and incongruence between nuclear and mtDNA markers. BMC Evol. Biol. 2009;9: 294.
79. Bragagnolo C, Pinto-da-Rocha R, Antunes M, Clouse RM. Phylogenetics and phylogeography of a long-legged harvestman (Arachnida: Opiliones) in the Brazilian Atlantic Rain Forest reveals poor dispersal, low diversity, and extensive mitochondrial introgression. Invertebr Syst. 2015;29: 386–404.
80. Peres EA, DaSilva MB, Antunes Jr M, Pinto-Da-Rocha R. A short-range endemic species from south-eastern Atlantic Rain Forest shows deep signature of historical events: phylogeography of harvestmen *Acutisoma longipes* (Arachnida: Opiliones). Syst. Biol. 2017. https://doi.org/10.1080/14772000.2017.1361479
81. Heyer WR, Maxson LR. Relationships, zoogeography, and speciation mechanisms of frogs of the genus Cycloramphus (Amphibia, Leptodactylidae). Arquiv. Zool. 1983;30(5): 341-373.
82. Mello FAG. Sistemática, distribuição e diferenciação de grilos do gênero *Aracamby* Mello, 1992 nas matas costeiras do sudeste do Brasil e sistema insular adjacente: aspectos morfológicos, cromossômicos e comportamentais (Orthoptera: Grylloidea: Phalangopsidae). PhD thesis, Instituto de Biociências, Universidade de São Paulo, Brazil. 1994.

83. Haffer J. Biogeography of Neotropical birds. In: Whitmore TC, Prance GT, editors. Biogeography and Quaternary history in tropical America. Oxford: Clarendon Press; 1987. p. 105-150.
84. Ledru MP, Rousseau DD, Cruz Jr FW, Riccomini C, Karmann I, Martin, L. Paleoclimate changes during the last 100,000 yr from a record in the Brazilian Atlantic rainforest region and interhemispheric comparison. Quarten Res. 2005;64: 444-450.
85. Saia SEMG, Pessenda LCR, Gouveia SEM, Aravena R, Bendassolli JA. Last glacial maximum (LGM) vegetation changes in the Atlantic Forest, southeastern Brazil. Quatern. Int. 2008;184: 195-201.
86. Jackson JF. Differention in the genera Enyalius and Strobilurus (Iguanidae): implications for Pleistocene climatic changes in eastern Brazil. Arq Zool Est São Paulo. 1978;30: 1-79.
87. Carnaval AC, Waltari E, Rodrigues MT, Rosauer D, VanDerWal J, Damasceno R, Prates I, Strangas M, Spanos Z, Rivera D, Pie MR, Frirkowski CR, Bornschein MR, Ribeiro LF, Moritz C. Prediction of phylogeographic endemism in an environmentally complex biome. Proc R Soc B. 2014;281: 1-8.
88. Almeida FFM, Carneiro CDR. Origem e evolução da Serra do Mar. Rev Bras Geoc. 1998;28(2): 135-150.
89. Riccomini C, Santanna L, Ferrari A. Evolução geológica do Rift Continental do Sudeste do Brasil. In: Mantesso Neto V, In: Bartorelli A,, In: Carneiro C,, In: Brito Neves B, eds. Geologia do Continente Sul-Americano: Evolução da Obra de Fernando Flávio Marques de Almeida. São Paulo: Beca; 2004. p. 383–405.
90. Saadi A, Bezerra FHR, Costa RD, Igreja HLS, Franzinelli E. 2005. Neotectônica da plataforma brasileira. In: Souza CRG, Suguio K, Oliveira AMS, Oliveira PE, editors. Quaternário do Brasil. Associação Brasileira de Estudos do Quaternário/Holos Editora, Ribeirão Preto; 2005. p. 211-234.

91. Behling H, Lichte M. Evidence of dry and cold climatic conditions at glacial times in tropical southeastern Brazil. Quat Res. 1997;48: 348–358

92. Ribeiro AC. Tectonic history and the biogeography of the freshwater fishes from the coastal drainages of eastern Brazil: an example of faunal evolution associated with a divergent continental margin. Neotrop. Ichthyol. 2006;4: 225–246.

93. Martin L, Suguio K, Flexro JM. As flutuações de nível do mar durante o Quaternário Superior e a evolução geológica de "deltas" brasileiros. Boletim IG-USP publicação especial. 1993;15: 1-186.

94. Suguio K, Angulo RJ, Carvalho AM, Corrêa ICS, Tomazelli LJ, Willwock JÁ, Vital H. Paleoníveis do mar e paleolinhas de costa. Quaternário do Brasil. In: Souza CRG, Suguio K, Oliveira MAS, Oliveira PE, editors. Quaternário do Brasil. Ribeirão Preto: Associação Brasileira de Estudos do Quaternário/Holos Editora; 2005. p. 114-129.

95. Rossetti DF, Góes AM. Marine influence in the Barreiras Formation, State of Alagoas, northeastern Brazil. An Acad Bras Ciênc. 2009;81: 741-755.

96. Cerling TE, Harris JM, MacFadden BJ, Leakey MG, Quadek J, Eisenmann V, Ehleringer JR. Global vegetation change through the Miocene/Pliocene boundary. Nature. 1997;389: 153-158.

97. Ravelo AC, Andreasen DH, Lyle M, Lyle AO, Wara MW. Regional climate shifts caused by gradual global cooling in the Pliocene epoch. Nature. 2004;429:263-267.

98. Holbourn A, Kuhnt W, Lyle M, Schneider L, Romero O, Andersen N. Middle Miocene climate cooling linked to intensification of eastern equatorial Pacific upwelling. Geology. 2014;42: 19-22.

99. IBGE 2012. Mapa da Área de Aplicação da Lei n° 11.428 de 2006 (1 : 5,000,000), 2nd edn. Rio de Janeiro: Fundação Instituto de Geografia e Estatística (IBGE).

Supplementary Files

Supplementary Figures

S1 Figure. Test for different number of categories used in GIE analysis with A) 3 categories, and B) 7 categories.

S2 Figure. Consensus-set NSC-NDM.

S3 Figure. Consensus-set SSC-NDM.

S4 Figure. Consensus-set SFP-NDM.

S5 Figure. Consensus-set ORG-NDM.

S6 Figure. Consensus-set SMSP-NDM

S7 Figure. Disjunct consensus-set which overlaps with other sets previously generated (SSC-NDM and SFP-NDM).

S8 Figure. Consensus-set POA-NDM.

S9 Figure. Consensus-set MIS-NDM.

Supplementary Tables

S1 Table. Geographic coordinates of the species used in the study. Data comes from literature (reference given) and our own unpublished records (a voucher specimen is mentioned as well as the way the specimen was identified). 'Name of sp. in NDM' reffers to the name of the species in the file S1 Material. Abbreviations: A1, anterior-most extremity of body; AC, copulatory apparatus; F, pharynx; Morph., morphology.

S2 Table. Records of land planarian species from literature excluded from this study and justification for such decision.

S3 Table. Geographic distribution of the land planarian species and their classification regarding their endemicity level. Abbreviations: CC, Congruence Core; WS, Widespread; MRE, Maximum Region of Endemism; SSC, South Santa Catarina; NSC, North Santa Catarina; Org: Serra dos Órgãos; PNSJ, Parque Nacional de São Joaquim; SFP, São Francisco de Paula; SMSP, Serra do Mar de São Paulo; SSP, Southern São Paulo; PR, Paraná; MIS, Misiones; POA, Porto Alegre; x, other cells.

Supplementary Material

S1 Material. Input file of NDM/VNDM ('xyd' file format).

Figure

Click here to access/download;Figure;Fig. 1.tif

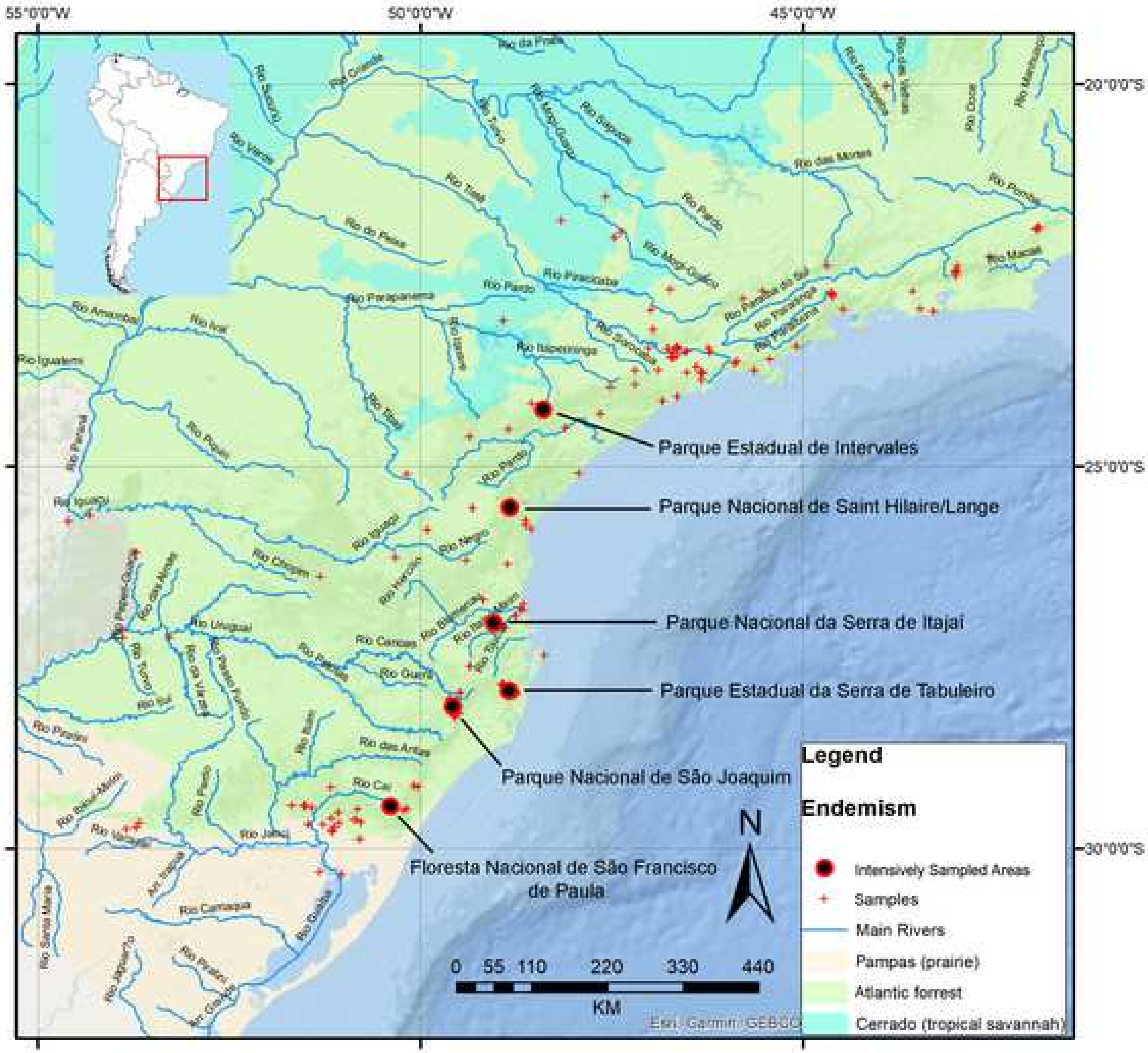

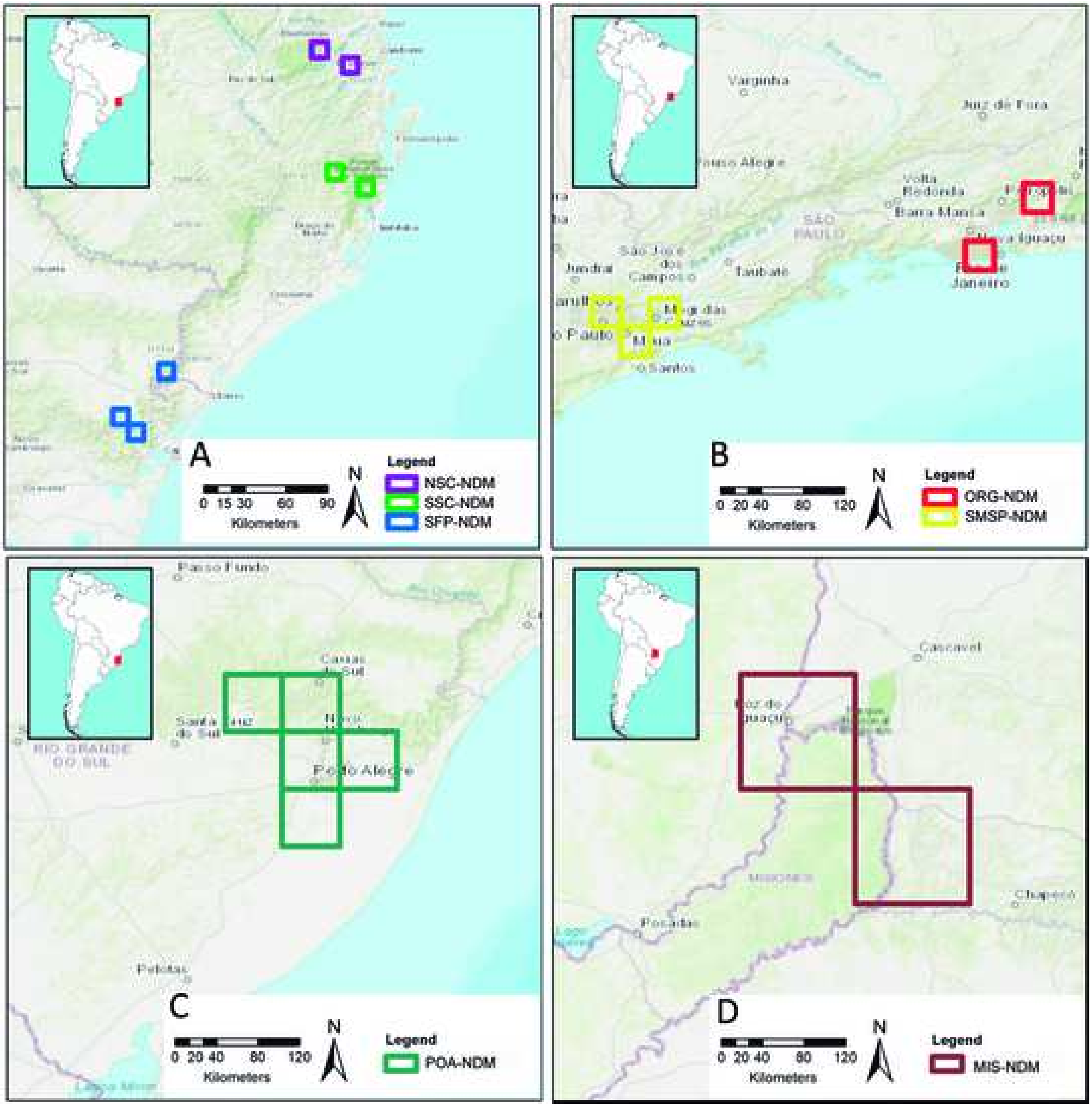
A
0 15 30 60 90
Kilometers
N
Legend
NSC-NDM
SSC-NDM
SFP-NDM
B
Varginha
Juiz de Fora
Volta Redonda
Barra Mansa
SÃO PAULO
Taubate
Jundiaí
São José dos Campos
Santos
Janeiro
0 20 40 80 120
Kilometers
N
Legend
ORG-NDM
SMSP-NDM
C
Passo Fundo
Caxias do Sul
RIO GRANDE DO SUL
Pelotas
0 20 40 80 120
Kilometers
N
Legend
POA-NDM
D
Cascavel
MISIONES
Posadas
Chapecó
0 20 40 80 120
Kilometers
N
Legend
MIS-NDM

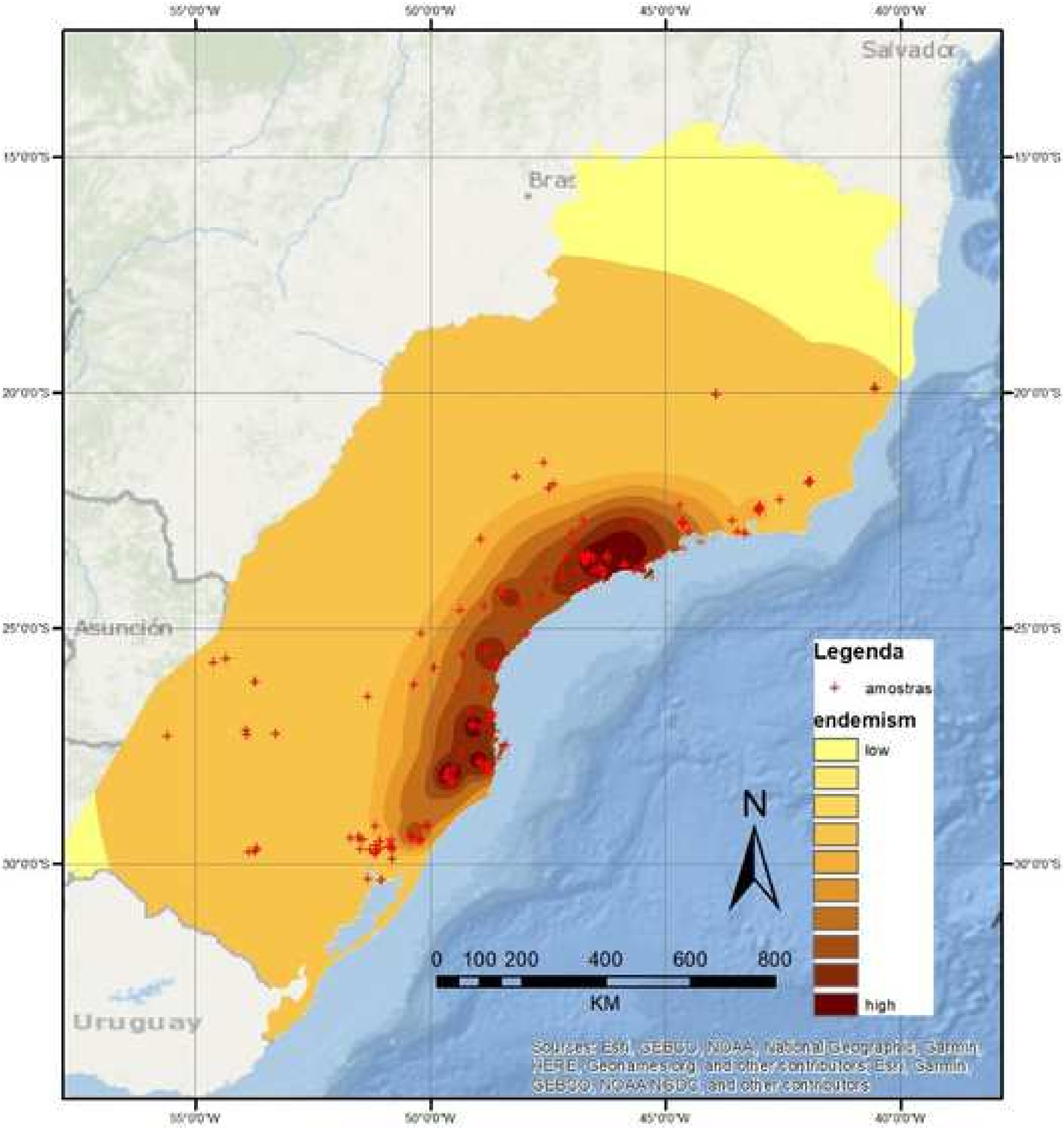

Salvador
Bras
Asunción
Uruguay
Legenda
amostras
endemism
low
high
N
0 100 200 400 600 800
KM
Sources: Esri, GEBCO, NOAA, National Geographic, Garmin, HERE, Geonames.org, and other contributors Esri, Garmin, GEBCO, NOAA NGDC, and other contributors

Figure

Click here to access/download;Figure;Fig. 4.tif

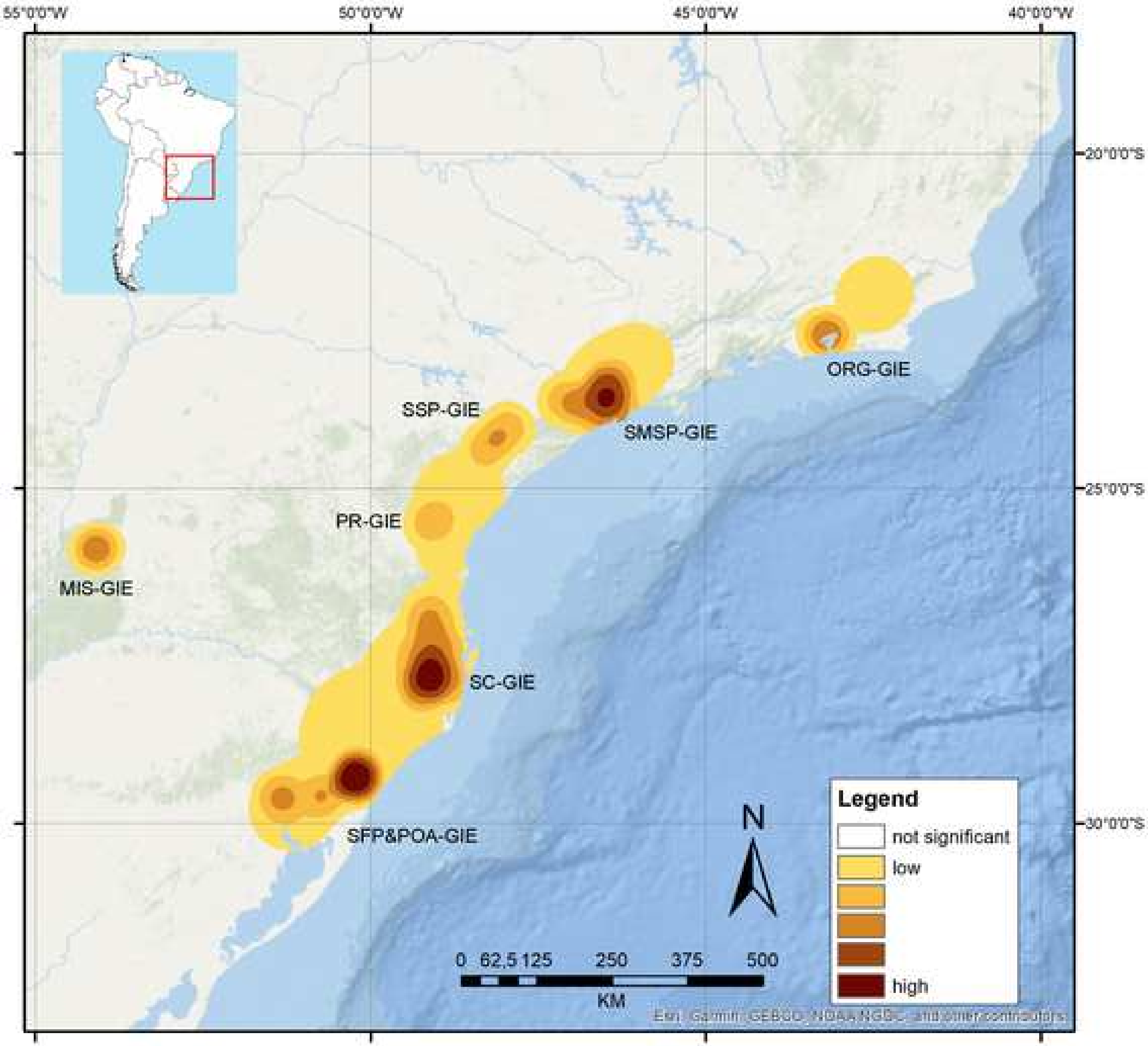

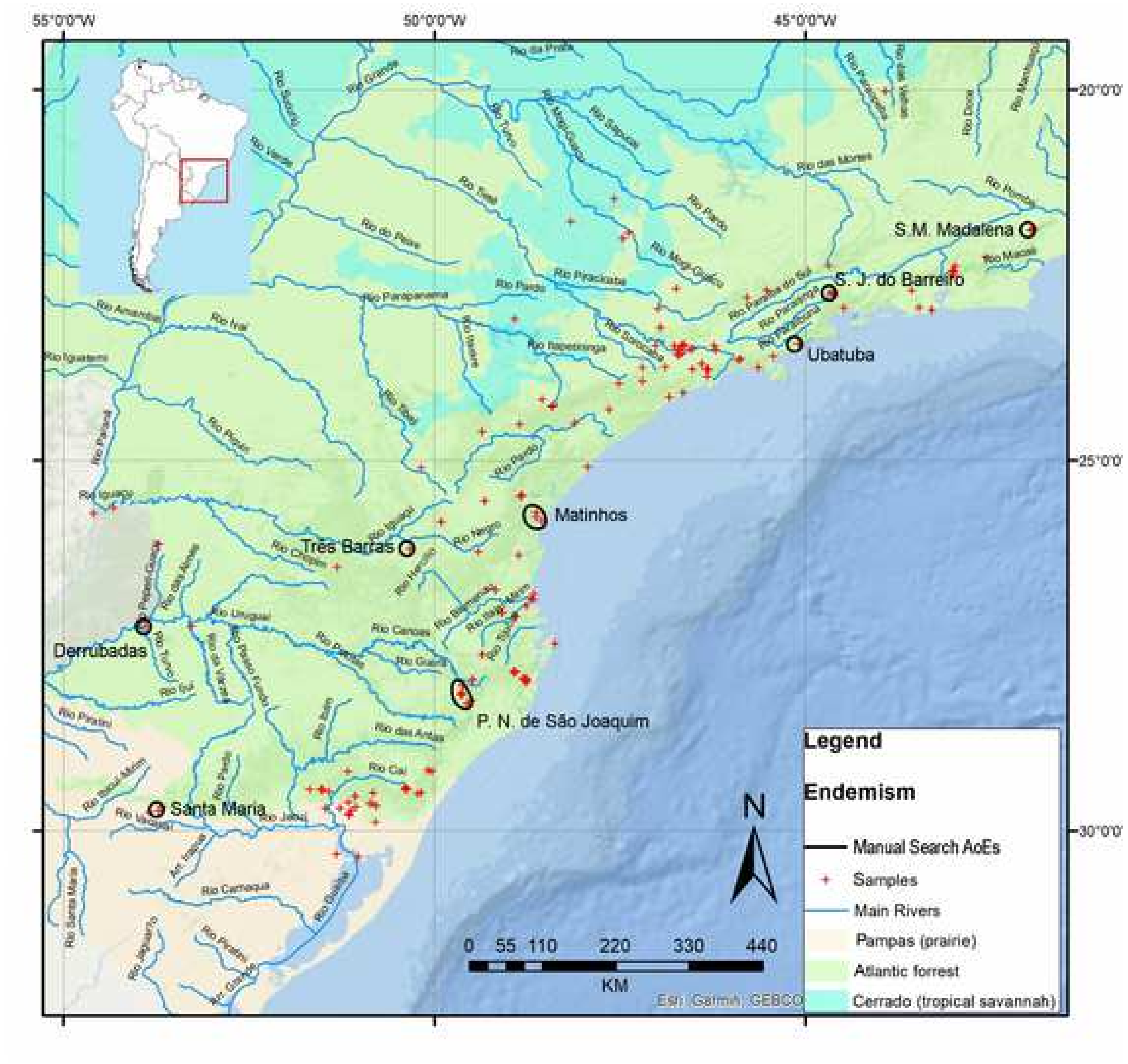
55°0'0"W
50°0'0"W
45°0'0"W
20°0'0'
25°0'0'
30°0'0'
S.M. Madalena
S. J. do Barreiro
Ubatuba
Matinhos
Tres Barras
Derrubadas
P. N. de São Joaquim
Santa Maria
N
0 55 110 220 330 440
KM
Esri, Garmin, GEBCO
Legend
Endemism
Manual Search AoEs
Samples
Main Rivers
Pampas (prairie)
Atlantic forrest
Cerrado (tropical savannah)

Figure Click here to access/download;Figure;Fig. 6.tif

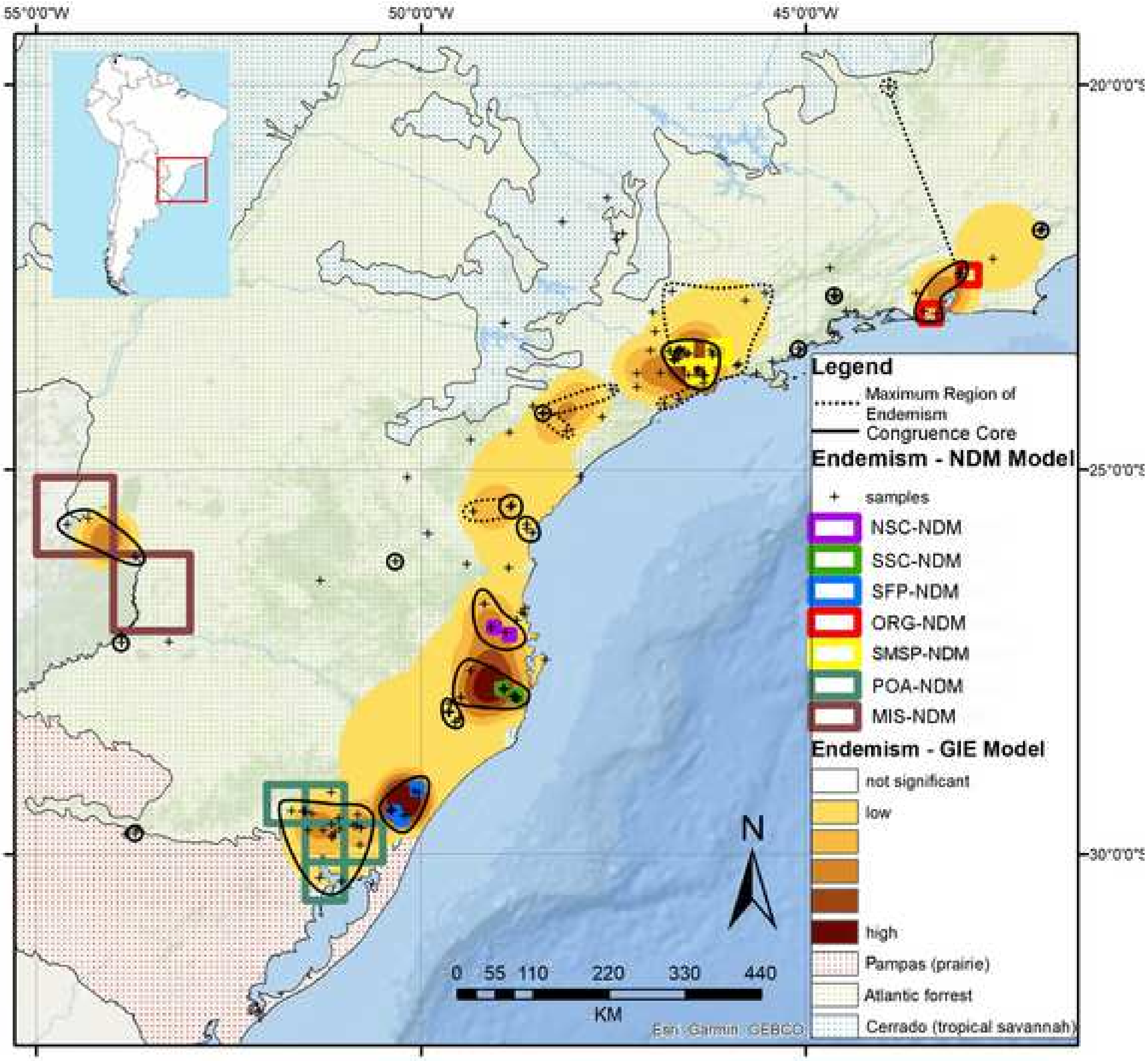

Figure

Click here to access/download;Figure;Fig. 7.tif

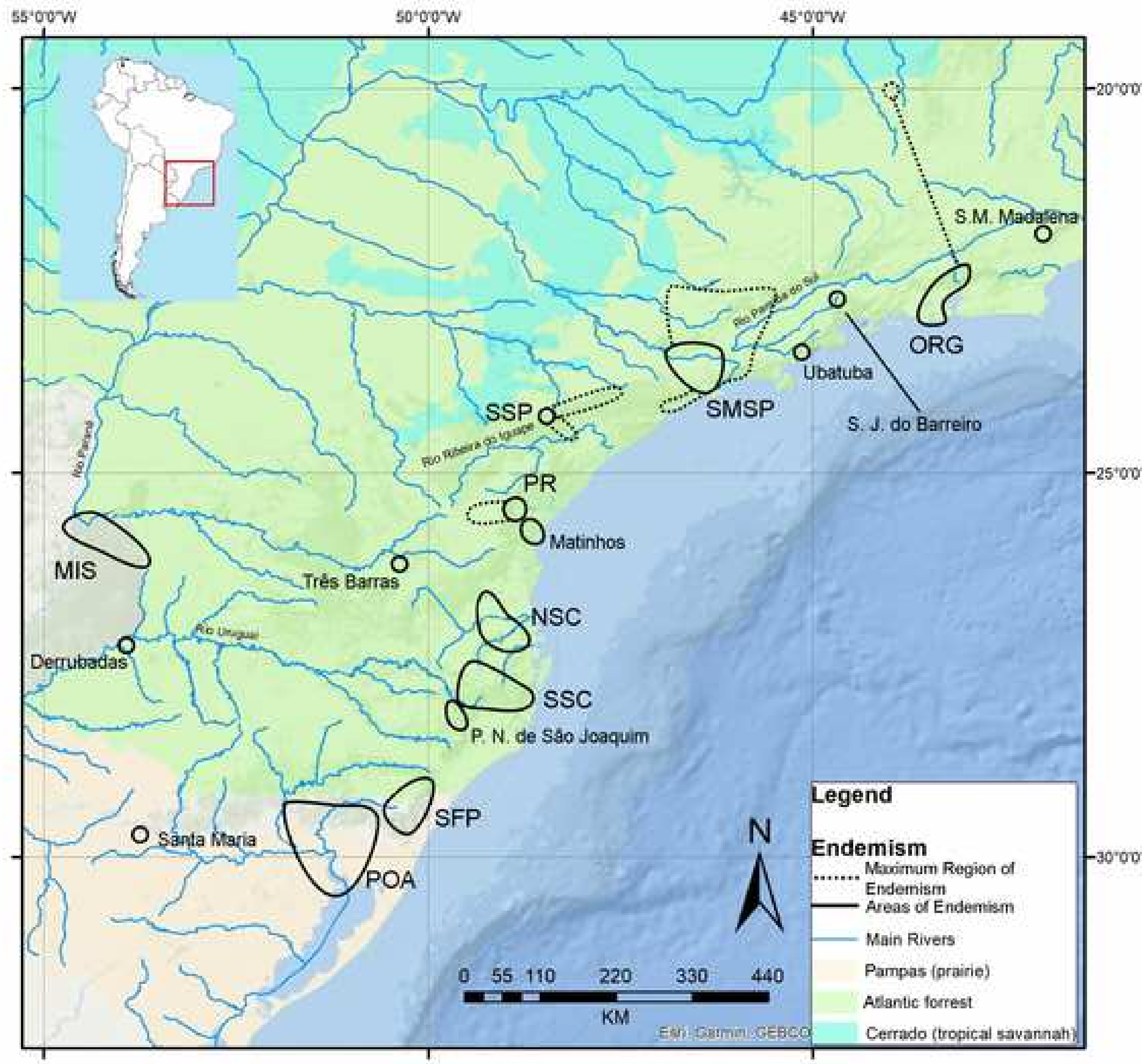

Supporting Figure

Click here to access/download
Supporting Information
S1 Figure.jpg

Supporting Figure

Click here to access/download
Supporting Information
S2 Figure.jpg

Supporting Figure

Click here to access/download
Supporting Information
S3 Figure.jpg

Supporting Figure

Click here to access/download
Supporting Information
S4 Figure.jpg

Supporting Figure

Click here to access/download
Supporting Information
S5 Figure.jpg

Supporting Figure

Click here to access/download
Supporting Information
S6 Figure.jpg

Supporting Figure

Click here to access/download
Supporting Information
S7 Figure.jpg

Supporting Figure

Click here to access/download
Supporting Information
S8 Figure.jpg

Supporting Figure

Click here to access/download
Supporting Information
S9 Figure.jpg

Click here to access/download
Supporting Information
S1 Material.xyd

Supporting Table

Click here to access/download
Supporting Information
S1 Table.docx

Supporting Table

Click here to access/download
Supporting Information
S2 Table.docx

Supporting Table

Click here to access/download
Supporting Information
S3 Table.docx
